# Phylogenetic relationships of *Pseudo-nitzschia subpacifica* (Bacillariophyceae) from the Mexican Pacific, and its production of domoic acid in culture

**DOI:** 10.1371/journal.pone.0231902

**Published:** 2020-04-24

**Authors:** Sonia Isabel Quijano-Scheggia, Aramis Olivos-Ortiz, Ernesto Garcia-Mendoza, Yaireb Sánchez-Bravo, Ramon Sosa-Avalos, Nathalli Salas Marias, Hong Chang Lim

**Affiliations:** 1 Centro Universitario de Investigaciones Oceanológicas, Universidad de Colima, Manzanillo, México; 2 Departamento de Oceanografía Biológica, Centro de Investigación Científica y de Educación Superior de Ensenada, Ensenada, Mexico; 3 Regal City College, Kuching, Sarawak, Malaysia; IRIG-CEA Grenoble, FRANCE

## Abstract

*Pseudo-nitzschia* is a cosmopolitan genus, some species of which can produce domoic acid (DA), a neurotoxin responsible for the Amnesic Shellfish Poisoning (ASP). In this study, we identified *P*. *subpacifica* for the first time in Todos Santos Bay and Manzanillo Bay, in the Mexican Pacific using SEM and molecular methods. Isolates from Todos Santos Bay were cultivated under conditions of phosphate sufficiency and deficiency at 16°C and 22°C to evaluate the production of DA. This toxin was detected in the particulate (DAp) and dissolved (DAd) fractions of the cultures during the exponential and stationary phases of growth of the cultures. The highest DA concentration was detected during the exponential phase grown in cells maintained in P-deficient medium at 16°C (1.14 ± 0.08 ng mL^-1^ DAd and 4.71 ± 1.11 × 10^−5^ ng cell^-1^ of DAp). In P-sufficient cultures DA was higher in cells maintained at 16°C (0.25 ± 0.05 ng mL^-1^ DAd and 9.41 ± 1.23 × 10^−7^ ng cell^-1^ of DAp) than in cells cultured at 22°C. Therefore, we confirm that *P*. *subpacifica* can produce DA, especially under P-limited conditions that could be associated with extraordinary oceanographic events such as the 2013–2016 "Blob" in the northeastern Pacific Ocean. This event altered local oceanographic conditions and possibly generated the presence of potential harmful species in areas with economic importance on the Mexican Pacific coast.

## Introduction

From 2013–2016 a confluence of abnormally warm water masses known as the “Blob” extended along the Northeast Pacific Ocean, between Alaska and Baja California [1], resulting from anomalies in atmospheric pressure that retained heat and increased sea surface temperature up to 4°C [2, 3]. These anomalous oceanographic conditions favored the development of one of the most intense and widespread Harmful Algae Bloom (HAB) of DA producing species in North Pacific coasts; specifically, *Pseudo-nitzschia australis* dominated this bloom [4]. The presence of the “Blob” also affected the phytoplankton composition in the northwestern region of the Mexican Pacific, resulting in significantly decreased abundances of diatoms and dinoflagellates, followed by the appearance of raphidophytes in 2016 with a negative impact in the local aquaculture industry [5]. Once this event ended in 2017, diatoms were again an important part of the phytoplankton community, along with the appearance of the genus *Pseudo-nitzschia* [5, 6].

In phytoplankton, diatoms–Class Bacillariophyceae–are one of the most species-rich classes, characterized by having a siliceous cell wall (frustule). *Pseudo-nitzschia* is one of the most common diatom genus, belonging to the Bacillariaceae family. It is a cosmopolitan genus that comprises at least 52 species [7–13]. It has been documented that many species are capable of producing the neurotoxin domoic acid (DA) under stressed conditions [11]. This capability has been confirmed in only 26 species [11, 12, 14]. Laboratory studies have shown that abiotic (P limitation, Si limitation, Nitrogen chemical species, temperature, etc.) and biotic factors (copepodamides, bacteria) enhance DA production of various *Pseudo-nitzschia* species [11, 15–18]. Blooms of DA-producing species can have widespread economic effects, resulting in the prohibition of molluscan shellfish harvesting and public health problems. The consumption of DA-contaminated shellfish can cause amnesic shellfish poisoning (ASP) in humans. Ecological problems related to poisoning and mortality of birds and marine mammals also appear when DA is present in the environment [11]. Therefore, it is important to identify and differentiate cryptic and pseudo-cryptic species in the genus *Pseudo-nitzschia* and recognize those that have the potential to produce DA [11, 16, 17].

So far, molecular analyses have proved to be a reliable approach to unambiguously identify *Pseudo-nitzschia* species. Of the tested molecular markers, the second internal transcribed spacer region (ITS2) of nuclear encoded ribosomal DNA appears to reliably differentiate between cryptic and pseudo-cryptic species. The secondary structure information in the ITS2 transcript have been used as characters for species delimitation, and appeared to correspond to patterns of sexual incompatibility and reproductive isolation [7, 19–23].

*Pseudo-nitzschia* is usually a common component within the phytoplankton community of the Mexican Pacific coast, where 14 species and two varieties have previously been reported [24–31]. However, most of the identifications of *Pseudo-nitzschia* species from the Mexican Pacific coast are based on morphological characters only, which leave doubts as to its correct identification. We isolated a strain of *Pseudo-nitzschia subpacifica* from samples collected during regular surveys to monitor HAB events in Todos Santos Bay and Mazanillo Bay on the Mexican Pacific coast. *Pseudo-nitzschia subpacifica* was originally described in Hasle [32] as *Nitzschia subpacifica*. It has been documented in Angola, Australia [33], Korea [34], China [35], Greece [36], Hong Kong [23], Peru [37] and Scotland coasts, [38] but has not been reported in the Mexican Pacific. DA production has been documented in only one strain isolated from Gulf of Maine [39]. Here we document the morphology, phylogenetic relationships and production of DA of *Pseudo-nitzschia subpacifica* strains isolated from Todos Santos Bay on the Mexican Pacific coast under laboratory conditions at sufficient and deficient availability of P at two temperatures (16°C and 21°C).

## Results

### Morphology

**Fig 1:**
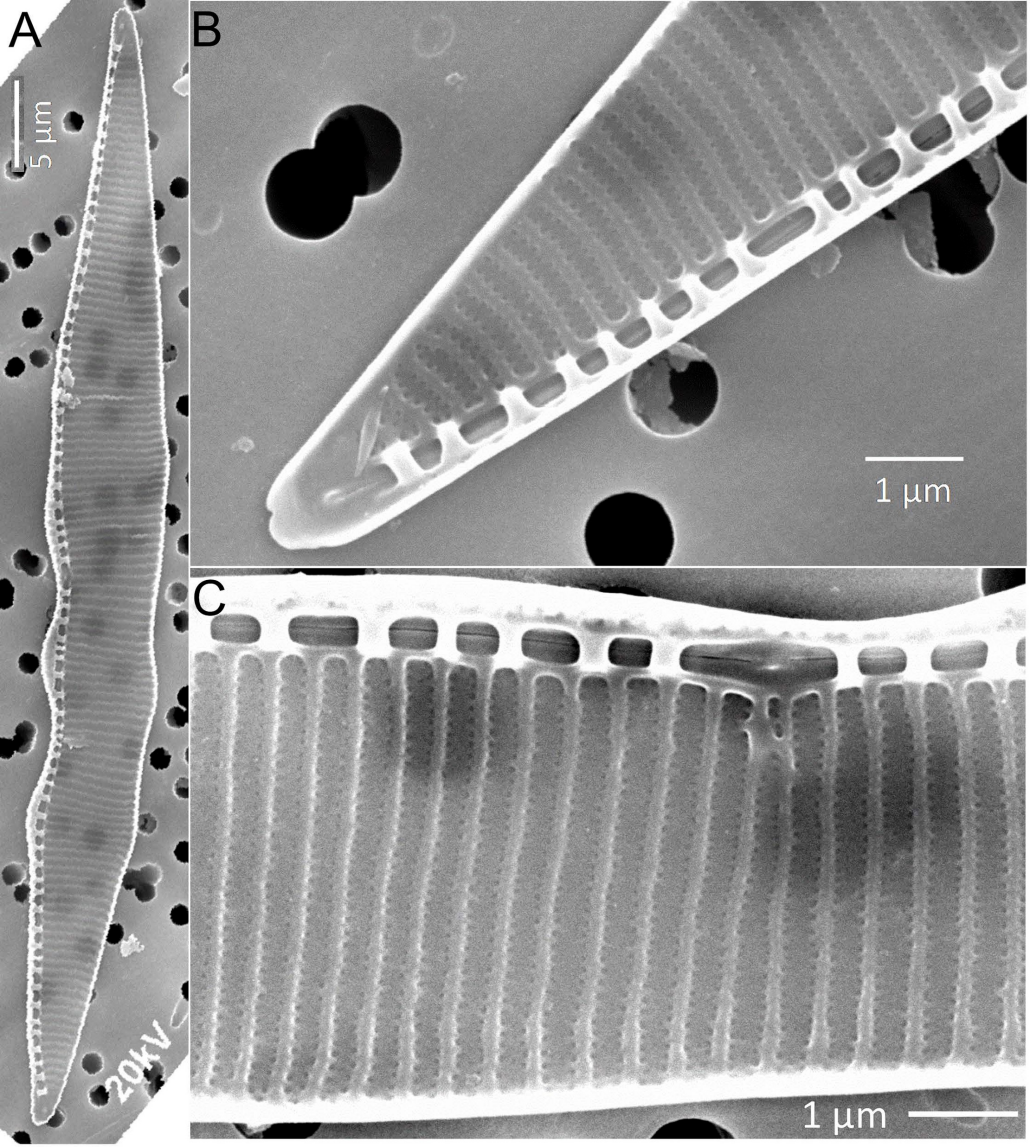
*Pseudo-nitzschia subpacifica* SEM micrographs of strain Ps 290. (A): Valve view of the whole valve showing central nodule, fibulae and striae. (B): Apex of the valve. (C): Details of fibulae and striae, showing two rows of poroids on each stria.

Morphological character of the strains isolated from Todos Santos Bay and Manzanillo Bay agree with the range of morphometric characters described in the references for *P*. *subpacifica* as described below (Table 1). Overlapping cells in colonies, cells were symmetrical and lanceolate in shape in valve view, tapering from the middle toward the tips. (Table 1, Fig 1A). The apical and transapical axes were 37–46 μm and 5.0–6.5 μm, respectively. A central larger interspace was present (Fig 1A). There were 16–18 regularly spaced fibulae and 26–28 striae in 10 μm (Table 1, Fig 1B and 1C).

**Table 1:** Morphometric measurements of *Pseudo-nitzschia subpacifica* in the present study, compare to previous reports. Each species listed possesses a central nodule.

Apical axis (μm)	Transapical axis (μm)	Fibulae in 10 μm	Striae in 10 μm	Rows of poroids in striae	Poroids in 1 μm	References
***P*. *subpacifica***						
37–46	5–6.5	16–18	26–28	2	8–10	Present study
33–70	5–7	15–20	28–32	2	9–10	[32, 40] original description as *Nitzschia subpacifica*
37–58	4.1–5.5	16–20	28–32	2	7–10	[41]
36–68	3.8–5.8	17–21	29–33	2	7–9	[39]
49–62	5–6.2	15–20	27–30	2	8–10	[42]
43–59	3.6–4.6	17–19	28–33	2	9–10	[35]
	3.9–4.3	17–21	29–32	2	7–10	[43]
47–79	4–6.3	14–20	28–30	2	8–10	[44]
	3.5–4.9	15–19	25–31	2	8–9	[45]
42–52	4.7–5.3	17	30	2	8–9	[37]
***P*. *bipertita***						
65–106.6	2.6–4.2	14–20	23–29	(1)2	5–8	[46]
***P*. *heimii***						
50–78	5–6	14–18	26–28	2	7–8	[40]
47–77	4–5	12–19	23–30	2	7–8	[47]
50–120	4–6	11–18	19–28	2	7–8	[48]
48–83	3.5–4.2	13–17	24–27	2	7–8	[49]
81–92	5.2–5.4	17	29	2	8	[39]

### Phylogenetic analyses

**Fig 2:**
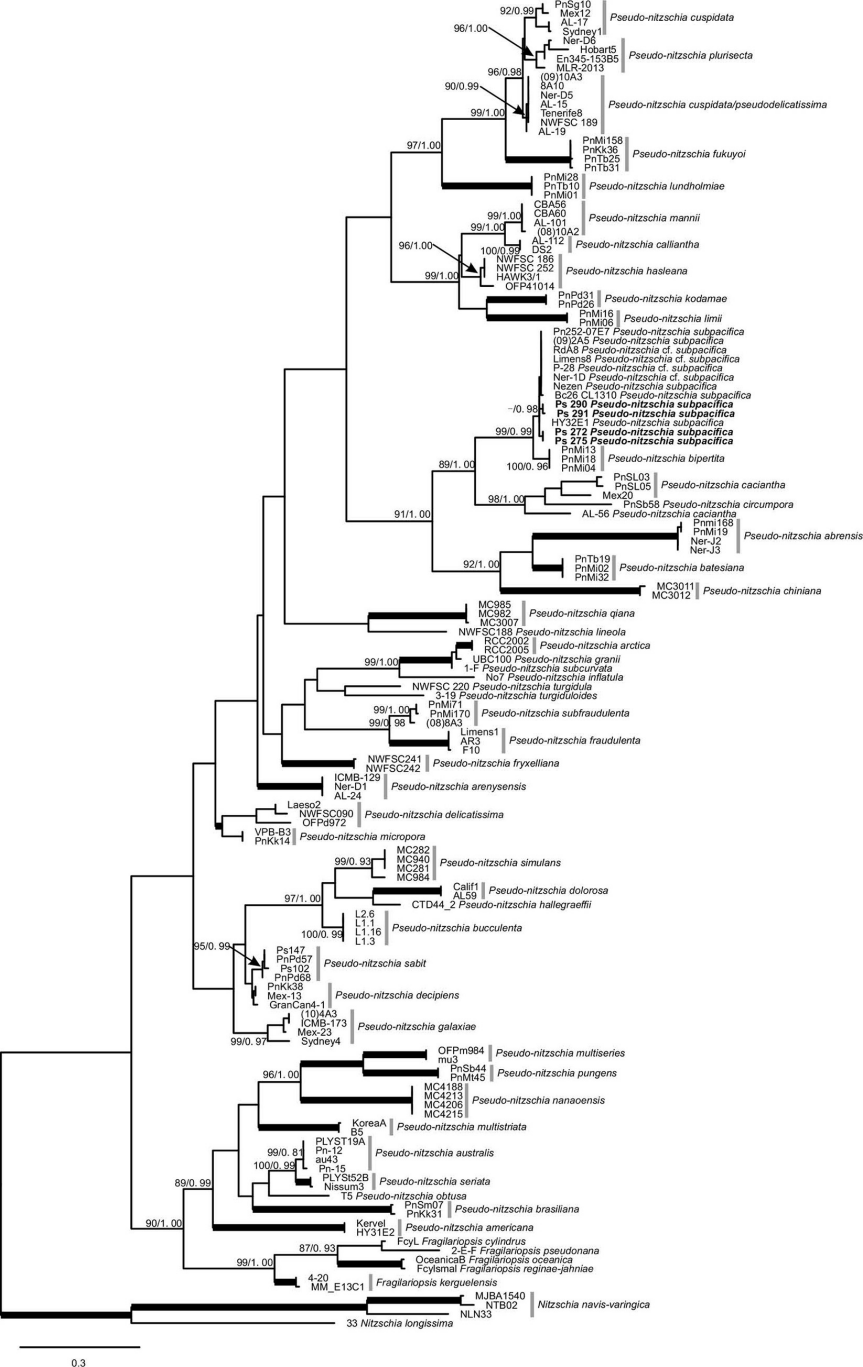
Tree topology inferred from ML based on the *Pseudo-nitzschia* ITS2 rDNA. The nodal supports are bootstrap values of Maximum Likelihood (ML), and Posterior Probability of Bayesian inference (BI). Values of 100% are marked with thick lines. “-” indicate bootstrap support of < 85%.

The ITS2 sequence-secondary structure alignment included 142 sequences with 662 characters. The best evolutionary models calculated for BI analysis was TVM+I+G from Akaike information criterion (AIC). The ML (Maximum Likelihood) and BI (Posterior Probability of Bayesian inference) phylogenetic trees were mostly congruent, except that the *Fragilariopsis* clade formed the basal to the whole *Pseudo-nitzschia* clade in ML tree (Fig 2) and formed a clade with *Pseudo-nitzschia* spp in BI tree (tree not shown). The topologies revealed that the Mexican Pacific strains (Ps 272 and Ps 275, Manzanillo Bay and Ps 290, Ps 291, Todos Santos Bay) belong to the *P*. *subpacifica* and formed a monophyletic clade with another 4 *P*. cf. *subpacifica* strains with ≥ 90 posterior probability (P.P.) in the analyses (ML: -, BI: 0.98) (Fig 2). While *Pseudo-nitzschia bipertita* being the closest sister clade to *P*. *subpacifica* showed a strong bootstrap support (ML: 99 and BI: 0.99).

The pairwise uncorrected *p*-distances for the ITS2 of *P*. *subpacifica/P*. cf. *subpacifica* strains isolated from the Mexican Pacific coast were compared to their closest relatives with interspecific divergence of 0.0033–0.040 for *P*. *bipertita*, 0.144–0.206 for *P*. *caciantha*, 0.193–0.204 for *P*. *circumpora*, 0.260–0.274 for *P*. *abrensis*, and 0.230–0.238 for *P*. *batesiana*.

### Secondary structure

**Fig 3:**
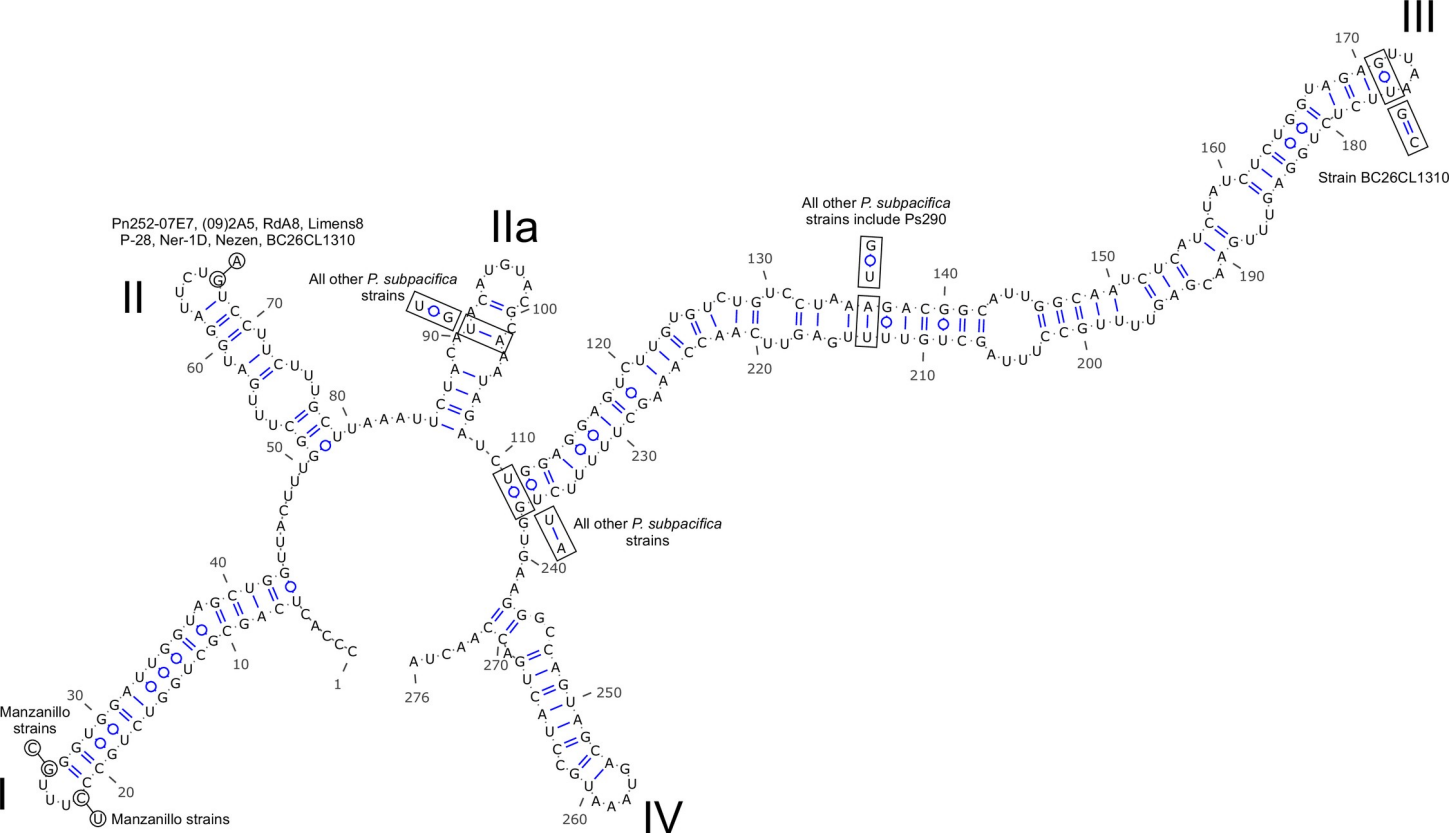
Secondary structure of *Pseudo-nitzschia subpacifica* strain Ps291 (Todos Santos Bay) ITS2, with comparisons to other *P*. *subpacifica* strains.

A four-helix structure (I–IV) with an additional helix, IIa, was identified, similar to previous findings [7, 8, 50]. One SNP was found at the base of helix II when compare the Todos Santos Bay strains (Ps290 and Ps291) with *Pseudo-nitzschia subpacifica* strains Pn252-07E7, (09)2A5, Nezen, Limens8, P-28, Ner-1D, BC26CL1310 and RdA8, listed in S1 Table. Another two SNPs were found at the base of helix I in Todos Santos Bay strains (Ps290 and Ps291) when compared with the Manzanillo strains (Ps272 and Ps275) (Fig 3). Additionally, in Todos Santos Bay strains (Ps290 and Ps291), one hemi-compensatory base change (HCBC) was found in helix IIa (U-A:U-G), two HCBCs in helix III (U-G:U-A, A-U:G-U) when compared with all the other *P*. *subpacifica* strains. One additional HCBC was found in basal segment of helix III in the Todos Santos Bay strains (Ps290 and Ps291) when compared with strain BC26CL1310 (Fig 3).

### Production of domoic acid under laboratory conditions

Table 2 shows nutrient concentration ratios in the cultures of Todos Santos Bay strains Ps 291 at different temperatures (16°C and 21°C) during the different days of the experiment. At the beginning of the experiment, for both temperatures the DIN:P ratio was close to 16 for P-sufficient conditions and 49 for P-deficient conditions, ensuring P-limitation of all cultures. As the experiment progressed, the variation in nutrient availability in DIN:P ratio continued for the conditions of sufficient/deficient P, with significant differences (P < 0.05) between treatments. The growth rate (μ) at 16°C was μ = 0.23 ± 0.18 in P-deficient medium and μ = 0.55 ± 0.1 in P-sufficient medium. In contrast, the growth rate at 21°C was μ = 0.23 ± 0.07 in P-deficient medium and μ = 0.3 ± 0.18 in P-sufficient medium.

**Table 2:** Concentration of particulate (DAp) and dissolved domoic acid (DAd) in *Pseudo-nitzschia subpacifica* in triplicate cultures grown at two temperatures in P-sufficient and P-deficient medium of strain Ps 291. The mean value and the standard deviation (SD) are shown.

Day culture	Mean DIN μM	Mean PO_4_^-3^ μM	Mean N:P	Dissolved DA ng mL^-1^		Particulate DA ng Cell^-1^		Total DA (DAp + DAd) fg cell^-1^	
**16°C P sufficient**									
1	2.53	0.16	16.20	ND	ND				ND
4	2.36	0.15	16.17	ND	ND				ND
7	2.27	0.13	17.17	0.25 (0.05)	9.41x10^-7^ (2.15x10^-7^)				2.25
11	2.20	0.13	17.17	0.23 (0.03)	1.11x10^-7^ (6.74x10^-8^)				0.26
			**16°C P deficient**						
1	2.55	0.05	49.06	ND	ND				ND
4	2.48	0.04	59.10	ND	ND				ND
7	2.36	0.03	69.53	1.14 (0.08)	4.71x10^-5^ (1.11x10^-5^)				137.10
11	2.25	0.03	77.41	1.07 (0.01)	9.57x10^-6^ (1.07x10^-5^)				38.17
			**22°C P sufficient**						
1	2.53	0.16	16.20	ND	ND				ND
4	2.48	0.15	16.76	ND	ND				ND
7	2.32	0.14	16.72	0.23 (0.014	1.97x10^-5^ (1.23x10^-5^)				63.40
11	2.29	0.13	17.88	0.19 (0.023)	1.27x10^-5^ (4.12x10^-6^)				33.90
			**22°C P deficient**						
1	2.55	0.05	49.06	ND	ND				ND
4	2.35	0.05	49.04	ND	ND				ND
7	2.10	0.04	55.32	0.88 (0.204)	1.37x10^-5^ (1.86x10^-5^)				32.60
11	1.93	0.03	56.65	0.79 (0.197)	6.20x10^-6^ (7.30x10^-6^)				18.80

ND No data

The highest concentration of DAd (1.14 ng mL^-1^) and DAp (4.71 × 10^−5^ ng cell^-1^) was recorded at 16°C under P-deficient conditions in the exponential phase of growth. At the stationary phase, the DAd concentrations decreased to 1.07 ng mL^-1^ and DAp to 9.57 × 10^−6^ ng cell^-1^, presenting significant statistical differences between DAd and DAp production (p = 0.00125). In relation to total domoic acid (DAt = DAd + DAp), we detected 137.10 fg cell^-1^ at the exponential phase and 38.17 fg cell^-1^ at the stationary phase which represents a decrease of the DAt of 72.16% between phases. DAt was higher in the exponential phase under the different conditions with significant differences with respect to the stationary phase (P < 0.05) (Table 2).

## Discussion

*Pseudo-nitzschia* is a cosmopolitan genus [51]. The present study is the first description of this species in the Mexican Pacific coast, and its identification is supported by morphological and molecular analyses (ITS2). The presence of *Pseudo-nitzschia subpacifica* could be related to the high temperatures caused by the “Blob” in 2016 that affected the taxonomic composition of the phytoplankton in this region, and when it ended in 2017 the abundance of the genus *Pseudo-nitzschia* increased, generating favorable oceanographic conditions so that after the "Blob" further growth of *P*. *subpacifica* would occur.

Morphologically, *P*. *subpacifica* closely resembles *P*. *heimii* since valve width, rows of poroids in 1 μm, and the presence of a central nodule are similar. However, both species can be readily distinguished by comparing the density of fibulae and striae and the number of poroids in 1 μm. *P*. *subpacifica* has a higher overall density (14–21, 25–33 and 7–10, respectively) of striae, compared to *P*. *heimii* (11–18, 19–30 and 7–8, respectively). *P*. *subpacifica* can also be misidentified as *P*. *bipertita*. The key characteristic to differentiate both species is that *P*. *bipertita* has 2 rows of poroids, sometimes 1 (poroids divided into 2–3 sectors), and a lower poroid density in 1 μm (5–8) [46]. *P*. *subpacifica* has only 2 rows of simple poroids and a higher overall poroid density in 1 μm (7–10) (Table 1).

Phylogenetic analyses confirmed that the strains isolated from Todos Santos Bay and Manzanillo Bay (Pacific Mexico) are *P*. *subpacifica* with a support (BI: 0.98.). Strains Limens, RdA8, P-28 and Ner-1D, formerly described as *P*. cf. *subpacifica* [45, 52] form a strongly supported clade (BI, P.P. 0.98) with other known *P*. *subpacifica* strains. In contrast, *P*. *subpacifica* shows an interspecific divergence of 3.3–4.0% with *P*. *bipertita*, the closest sister taxon. Sexual compatibility and ITS2 secondary structure were widely implemented in the description of new *Pseudo-nitzschia* species [53, 54], in our case with 4 HCBCs within the *P*. *subpacifica* clades, there is a possibility of the presence of cryptic/pseudo-cryptic species in within the *P*. *subpacifica* strains/sequences analyzed (Fig 3). *Pseudo-nitzschia mannii* differed from *P*. *calliantha* by having no CBCs, but 4 HCBCs, which is supported by mating incompatibility experiment [55]. Hence, more strains of *P*. *subpacifica* should be tested for their morphology, genetics and mating compatibility to rule out the presence of cryptic species.

In this work, we report that *Pseudo-nitzschia subpacifica* isolated from the northwest Pacific coast of Mexico produced DA under culture conditions. Previously, in this species isolated from the Gulf of Maine in October of 2007, but maintained under different culture conditions; f/2 medium, at 10°C and under low light levels, it reported DAd production (0.06–1.1 ng mL-^1^) [39], similar to the concentrations detected in this study (0.19 to 1.14 ng mL-1 of DAd). Our results are comparable to those reported by Amato [56], where *P*. *multistriata* cultures under phosphate limitation (3.0 μM) and low light treatment were induced to the production of intracellular domoic acid (<6.0 fg cell^-1^ after 7 days of incubation). Laboratory studies have shown that abiotic conditions like salinity or N:P ratio affect the growth and DA production of various *Pseudo-nitzschia* species [11, 16, 17, 57]. Also, model simulations demonstrated that the excess light, combined with P-deficiency, can promote DA production [11, 16, 17]. Given non-limiting concentrations of N, the lack of P is one factor that likely triggers the production of DA, a secondary metabolite [16].

It is interesting to note that *P*. *subpacifica* strains from Mexico produced DAd and DAp at 22°C in the P-sufficient medium, whereas DA concentration was the highest at 16°C in P-deficient medium. Apparently, a variation in temperature, with P-deficient medium inducing DA production, was also observed for *P*. *australis* [58, 59]. It has also been reported that some species of *Pseudo-nitzschia* produce DA under limitation by other nutrients (N and Si) during the exponential phase of growth [11, 17]. These studies show that abiotic factors influence the toxicity of a culture as a response to external stressors. It is necessary to explore the change in the gene expression under stress conditions and the possible upregulation of genes linked to the activation of the DA biosynthesis. In culture under phosphate deficiency conditions of *Pseudo-nitzschia multistriata* and *Pseudo-nitzschia multistriata australis*, DabACD-dependent biosynthetic pathway to isodomoic acid is established, encoded by the *dab* genes [60]. Identifying these genes provides an additional tool to the currently two established method to determine DA in algal blooms; mass spectrometry and enzyme-linked immunosorbent assay.

DAd constituted an important fraction of DAt detected in the cultures (until 72.16%). Other studies have also reported that the major fraction of DA found in cultures of other species is present in the dissolved form: Pan, Parsons [61] for *P*. *pseudodelicatissima*; Santiago-Morales and García-Mendoza [29] for *P*. *australis*, and Godinho, Silva [62] for *P*. *multiseries*. It is not clear, if the DA produced by the cells is released during cell lysis or if it is excreted actively into the medium. This must be investigated, as well as the possible ecological role of DAd in the environment.

Finally, although identification of the genus *Pseudo-nitzschia* is difficult because it involves electron microscopy and molecular methods that are time consuming and have high costs, it is important to identify the *Pseudo-nitzschia* species present in each region, to characterize potential DA production and to identify the oceanographic processes associated with the presence of potentially harmful species. This can help mitigate regional damage to the economy and public health by HABs, including along the Mexican Pacific coast.

## Materials and methods

**Fig 4:**
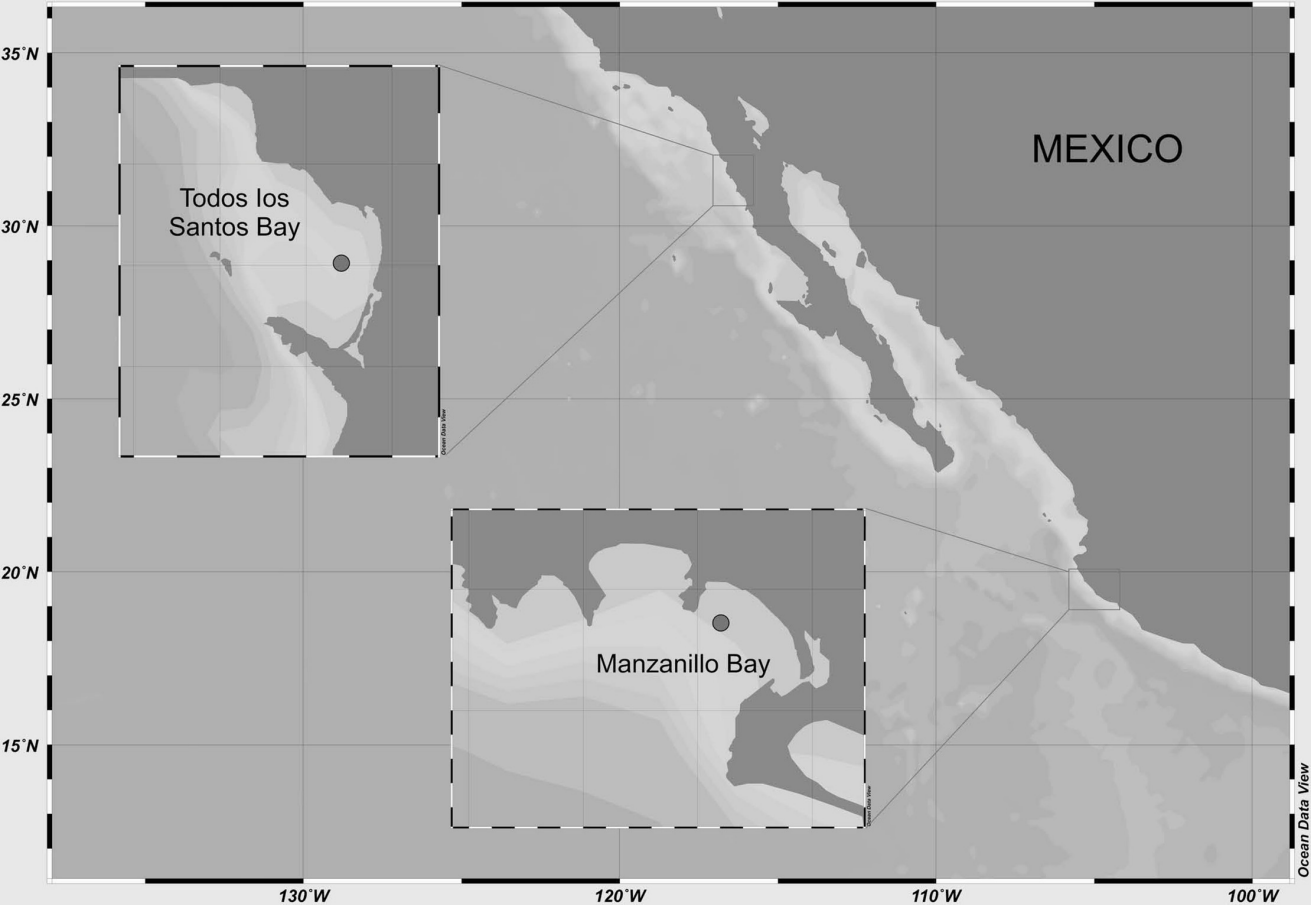
Map of the Mexican Pacific coast showing Todos Santos and Manzanillo Bays.

No specific permissions were required from the Semarnat authority (Ministry of Environment and Natural Resources) for the samples of phytoplankton in Todos Santos Bay, Baja California, and from Manzanillo Bay, Colima, located on the Mexican Pacific coast. The field studies did not involve endangered or protected species. Water samples were collected in Todos Santos Bay, Baja California, and from Manzanillo Bay, Colima, located on the Mexican Pacific coast (Fig 4). According to local monitoring programs, both locations were under the influence of the “Blob” in March of 2017. Live samples were examined under a Motic AE31 inverted microscope (Ted Pella, Inc., Redding, California, USA). Chains of *Pseudo-nitzschia* spp. were isolated with a glass Pasteur micropipette. Cultures were established in L1 medium [63], at a salinity of 30. The cultures were maintained (in triplicate) at 21°C, with a 12:12 h light:dark (L:D) photoperiod. Illumination was provided by fluorescent tubes (Phillips F96T12/TL865/EW, 60W, USA) at an irradiance of 100 μmol quanta m^-2^ s^-1^. Cells were harvested during the exponential and stationary phase of growth and were frozen for morphological and molecular analysis. A Sedgewick-Rafter chamber and a Motic AE31 inverted microscope was used for determination of cell abundance [64].

### Morphological analysis

Organic material was removed from the samples with sulfuric acid and potassium permanganate, followed by the addition of oxalic acid [65]. The remaining material was mounted on a polycarbonate filter, attached to stubs by colloidal silver, sputter-coated with gold, and then observed at 20–30 kV in a JEOL JSM-390 LV scanning electron microscope (SEM). *Pseudo-nitzschia* cells were examined for the following morphometric character: width and length of the valve, and density of striae, fibulae and poroids.

### Molecular analysis: DNA extraction, PCR and sequencing

*Pseudo-nitzschia* cells were harvested during the exponential phase of growth, concentrated by centrifugation (10,000 rpm for 10 min) and frozen at −20°C. The DNA was extracted as described by [66]. The Internal Transcribed Spacer (ITS1-5.8S-ITS2) region of the rDNA was amplified and sequenced using ITS1 and ITS4 universal primers [50, 67] under the following PCR conditions: initial denaturation at 94°C for 2 min, followed by 35 cycles, each cycle comprising 94°C for 35 s, 60°C for 35 s, and 72°C for 1 min, followed by a final extension step at 72°C for 5 min (ESCO Swift MiniPro Thermal Cycler. Model SWT-MIP-0.2–1). The amplifications were performed with the kit GoTaq DNA Polymerase, 50 μL reaction using DNA template from 40–100 ng. The PCR products were visualized in 1% agarose gel with ethidium bromide staining. The purified PCR products were Sanger-sequenced with BigDye terminator v3.1 sequencing kit and a 3730xl automated sequencer (Applied Biosystems, Foster City, CA USA). Nucleotide sequences were determined on both strands of PCR amplification products at the Macrogen sequencing facility (Macrogen Inc., Seoul, Korea) using an ABI Prism 3730xl Analyzer. The obtained sequences were deposited in GenBank.

### Alignment and phylogenetic analysis

Only ITS2 of the ITS region was used in this study. This ITS rDNA sequence was aligned with a total of 141 *Pseudo-nitzschia* sequences (S1 and S2 Tables) retrieved from the NCBI nucleotide database by 4SALE [68] as detailed in Lim, Tan [8]. The final data set of ITS2 comprised 141 taxa with 1 outgroup. The ITS2 analyses were rooted with a sequence of *Nitzschia longissima*. ML analyses were carried out with Phangorn in which the model parameters was directly estimated from the aligned data set [8, 69]. Bootstrap support values were obtained based on 500 bootstrap replicates. The BI (Bayesian) analyses of ITS2 were carried out using MrBayes v3.2.6 [70] following the parameters outlined in Lim et al. [8], with 2,650,000 Markov chain Monte Carlo generations, sample frequency was 100, the number of burn-in generations was 6625, and the best model obtained as detailed in Lim et al. [8].

### Genetic distance

The pairwise genetic distances, based on *p*-distance, were estimated using MEGA 7 [71].

### Secondary structure of RNA

The secondary structure of ITS2 for the *Pseudo-nitzschia* strains obtained in the present study (Ps272 and Ps275 from Manzanillo Bay, and Ps290 and Ps291 from Todos Santos Bay) was predicted using ITS2 Database III [72–76]. Secondary ITS2 transcript structures were illustrated by using VARNA [77]. The ITS2 of all the sequences of each *P*. *subpacifica* strain (our four, and strains Nezen and RdA8) were compared using the 4SALE program [78] to identify compensatory base changes (CBCs) and hemi-compensatory base changes (HCBCs). The helices were named according to Mai and Coleman [79] and Amato [80].

### Domoic acid production under laboratory conditions

Production of DA was evaluated only in *P*. *subpacifica* isolated from Todos Santos Bay (Ps 290, Ps 291). The cells were maintained (in triplicate) in 500 mL Erlenmeyer flasks with L1 culture medium with sufficient or deficient P. The growth and production of DA under both conditions were evaluated at 16°C and 22°C. Triplicate cultures were maintained at each condition with a 12:12 h light:dark (L:D) photoperiod at 100 μmol quanta m^-2^ s^-1^.

Samples to quantify the presence of DA in particulate matter (DAp) and in the medium (DAd) were taken during the middle of the exponential and stationary stages of growth of the cultures. Cells were collected by low-vacuum filtration of 100 mL of culture onto membrane filters (Millipore; 0.45 μm pore size, 47 mm diameter. The filtrate was recovered to analyze the DAd fraction.

The concentration of phosphate (PO_4_^3-^) and dissolved inorganic nitrogen (DIN: NO_3_^-^ + NO_2_^-^ + NH_4_^+^) was determined using a Scalar SanPlus segmented flow auto analyzer according to colorimetric techniques [81, 82]. The stoichiometric relationship [83] was used to determine the possibility of P limitation in our cultures, whereby the availability of N with respect to P has a DIN:P ratio of >22. No tests were performed with variations in the Si concentration due to technical limitations in obtaining Si-free culture media.

Domoic acid (DA) concentration was determined by HPLC-MS/MS. Toxin extraction and analysis were as in Mafra *et al*. [84] with some modifications. Extraction of DAp was done by mechanical disruption (Mini-BeadBeater^TM^, Biospec Inc. USA) of the filters collected form the culture with 0.5 mm diameter zirconia/glass beads in 1 mL of 50% (v:v) water:MeOH. Samples were clarified by two centrifugation steps (13000 rpm for 8 min, 13000 rpm for 5 min, 4°C). DAd was quantified in the culture medium that passed thought the filter. TFA was added to each sample prior to analysis at final concentration of 0.15%. DAp and DAd concentrations were quantified using an Agilent 1290 Infinity II HPLC coupled with an Agilent 6470 triple quadrupole mass spectrometer equipped with electrospray ionization (ESI). A ZORBAX Eclipse Plus C18 RRHD column (2.1 x 100 mm 1.8 μm) at 45°C was used with a linear gradient of elution from 10% to 35% acetonitrile in 0.2% formic acid in 8 min. The flow rate was 0.25 mL min^-1^ and 20 μL of the sample was injected into the system. The detection of DA was achieved using positive ionization, sheath gas (= N_2_) at flow of 11 L^-1^ min. The transitions monitored in multiple reaction monitoring (MRM) mode were m/z 312.2→266.1 and m/z 312.2→161. Certified reference material CRM-DA-g (103.3 μg mL^-1^) was obtained from the National Research Council (NRC) of Canada to create a six-point calibration curve from 0.1 to 50.0 ng mL^-1^. The detection limit was considered as the lower calibration point (0.1 ng mL^-1^), which was more than 3 times the signal to noise ratio.

Because triplicate experiments were performed to determine DA production under sufficient and limited P conditions, the T-student test was used to determine if there were significant differences between treatments at p <0.05 using the Statistica program [85].

## Supporting information

S1 Data (PDF)

S1 Table List of ITS rDNA sequences of *Pseudo-nitzschia* used in phylogenetic studies. (DOCX)

S2 Table (DOCX)
