# Supplementary material for: Phylogenetic relationships of Pseudo-nitzschia subpacifica (Bacillariophyceae) from the Mexican Pacific, and its production of domoic acid in culture
Source: PLoS One. 2020 Apr 24;15(4):e0231902. doi: 10.1371/journal.pone.0231902 (PMC7182257; doi:10.1371/journal.pone.0231902)
Supplement: S1 Data — (PDF) [file pone.0231902.s001.pdf]

OCEAN DATA VIEW LICENSE AGREEMENT

By downloading and / or using the Ocean Data View Software (ODV), you agree to be bound by the following legal agreement between you and the Alfred Wegener Institute, Helmholtz Centre for Polar and Marine Research (AWI). If you do not agree to the terms of this Agreement, do not download and / or use the Software.

1. SCIENTIFIC USE AND TEACHING

Ocean Data View is allowed to be used free of charge for non-commercial, non-military research and teaching purposes only. If you use the software for your scientific work, please reference Ocean Data View in your publications as follows: Schlitzer, R., Ocean Data View, https://odv.awi.de, 2018.

2. COMMERCIAL USE

For the use of Ocean Data View or any of its components for commercial applications and products, a special, written software license is needed. Please contact the address below for further information.

3. MILITARY USE

AWI focusses its activities on civil purposes. Licenses for the Ocean Data View Software for military purposes will only be granted after case-by-case assessments of the requests. Please contact the address below for further information.

4. REDISTRIBUTION

Redistribution of the Ocean Data View software on CD-ROM, DVD, or other electronic media or the Internet is not permitted without the prior written consent of the AWI. Please contact the address below for further information.

5. WARRANTY DISCLAIMER

THE ODV SOFTWARE IS PROVIDED "AS IS" WITHOUT WARRANTY OF ANY KIND, EITHER EXPRESSED OR IMPLIED, INCLUDING, BUT NOT LIMITED TO, THE IMPLIED WARRANTIES OF MERCHANTABILITY AND FITNESS FOR A PARTICULAR PURPOSE. THE ENTIRE RISK AS TO THE QUALITY AND PERFORMANCE OF THE SOFTWARE IS WITH YOU. SHOULD THE SOFTWARE PROVE DEFECTIVE, YOU ASSUME THE COST OF ALL NECESSARY SERVICING, REPAIR OR CORRECTION.

IN NO EVENT WILL AWI, ITS CONTRIBUTORS OR ANY ODV COPYRIGHT HOLDER BE LIABLE TO YOU FOR DAMAGES, INCLUDING ANY DIRECT, INDIRECT, GENERAL, SPECIAL, EXEMPLARY, INCIDENTAL OR CONSEQUENTIAL DAMAGES HOWEVER CAUSED AND ON ANY THEORY OF LIABILITY ARISING OUT OF THE USE OR INABILITY TO USE THE SOFTWARE (INCLUDING BUT NOT LIMITED TO LOSS OF DATA OR DATA BEING RENDERED INACCURATE OR LOSSES SUSTAINED BY YOU OR THIRD PARTIES, A FAILURE OF THE SOFTWARE TO OPERATE WITH ANY OTHER SOFTWARE OR BUSINESS INTERRUPTION).

© 1990 - 2018 Reiner Schlitzer, Alfred Wegener Institute, Am Alten Hafen 26, 27568 Bremerhaven, Germany, E-Mail: Reiner.Schlitzer@awi.de
