# Supplementary material for: Phylogenetic relationships of Pseudo-nitzschia subpacifica (Bacillariophyceae) from the Mexican Pacific, and its production of domoic acid in culture
Source: PLoS One. 2020 Apr 24;15(4):e0231902. doi: 10.1371/journal.pone.0231902 (PMC7182257; doi:10.1371/journal.pone.0231902)
Supplement: S1 Table — (DOCX) [file pone.0231902.s002.docx]

Table S1. List of ITS rDNA sequences of *Pseudo-nitzschia* used in phylogenetic studies

| Taxon | Strain designation | Origin | Accession no. ITS |
| --- | --- | --- | --- |
|  |  |  |  |
| *Fragilariopsis nana* | 2-E-F | Denmark | EF660058 |
| *Fragilariopsis nana* | ErikaFcyl | Denmark | EF660059 |
| *Fragilariopsis nana* | PA_plate12A | Denmark | EF660060 |
| *Fragilariopsis cylindrus* | Fcyl | Denmark | EF660057 |
| *Nitzschia longissima* | strain 33 | China | KJ671772 |
| *Pseudo-nitzschia abrensis* | NerJ3 | Abra of Bilbao, Spain | KC409109 |
|  | NerJ2 | Abra of Bilbao, Spain | KC409108 |
|  | Pnmi19 | Miri, Sarawak, Malaysia | KR021327 |
|  | Pnmi168 | Miri, Sarawak, Malaysia | KR021324 |
| *Pseudo-nitzschia americana* | HY31E2 | Korea | LC194944 |
|  | Kervel | - | EU523099 |
| *Pseudo-nitzschia arctica* | RCC2002 | Beaufort Sea, Canada | KT808253 |
|  | RCC2005 | Beaufort Sea, Canada | KT808255 |
| *Pseudo-nitzschia arenysensis* | Ner-D1 | Nervion River Estuary, Spain | GQ228393 |
|  | AL-24 |  | DQ813830 |
|  | ICMB-129 | Catalan coast, Spain | EU367951 |
| *Pseudo-nitzschia australis* | au43 | Monterey Bay, California, USA | DQ062661 |
|  | Pn 12 | California, USA | KC329500 |
|  | Pn 15 | California, USA | KC329501 |
|  | PLYSt19A | Scottish waters | AY452527 |
| *Pseudo-nitzschia batesiana* | PnTb19 | Teluk Batik, Perak, Malaysia | KC147514 |
|  | PnMi02 | Miri, Sarawak, Malaysia | KR021328 |
|  | PnMi32 | Miri, Sarawak, Malaysia | KX572953 |
| *Pseudo-nitzschia bipertita* | PnMi04 | Miri, Sarawak, Malaysia | KR021318 |
|  | PnMi13 | Miri, Sarawak, Malaysia | KR021320 |
|  | PnMi18 | Miri, Sarawak, Malaysia | KR021319 |
| *Pseudo-nitzschia brasiliana* | PnSm07 | Samariang Batu, Sarawak, Malaysia | HQ111404 |
|  | PnKk31 | Kota Kinabalu, Sabah, Malaysia | JN252429 |
| *Pseudo-nitzschia bucculenta* | L2.6 | Namibian | MH376339 |
|  | L1.16 | Namibian | MH376342 |
|  | L1.3 | Namibian | MH376341 |
|  | L1.1 | Namibian | MH376340 |
| *Pseudo-nitzschia caciantha* | AL-56 | Gulf of Naples, Italy | DQ813834 |
|  | Mex20 | Tuxpam, Gulf of Mexico, Mexico | AY257861 |
|  | PnSL03 | Sibu Laut, Sarawak, Malaysia | KF482055 |
|  | PnSL05 | Sibu Laut, Sarawak, Malaysia | KF482056 |
| *Pseudo-nitzschia calliantha* | AL-112 | Gulf of Naples, Italy | DQ813841 |
|  | DS2 | Denmark | AY257856 |
| *Pseudo-nitzschia circumpora* | PnSb58 | Santubong, Sarawak, Malaysia | JN252430 |
| *Pseudo-nitzschia cuspidata* | AL-17 | Gulf of Naples, Italy | DQ813827 |
|  | NWFSC 189 | Washington, USA | KX572955 |
|  | NWFSC 190 | Washington, USA | JN091757 |
|  | NWFSC 191 | Washington, USA | KX572956 |
|  | NWFSC 194 | Washington, USA | JN050289 |
|  | PnSg10 | St. John Island, Singapore | KX572957 |
|  | Sydney1 | Bondi Beach, Sydney, Australia | AY257862 |
|  | Mex12 | Near Tuxpam, Mexico | AY257852 |
|  | Tenerife8 | Tenerife, Canary Islands | AY257853 |
| *Pseudo-nitzschia decipiens* | GranCan4-1 | Canary Islands | DQ336157 |
|  | Mex13 | Tuxpam, Gulf of Mexico, Mexico | DQ336156 |
|  | PnKk38 | Kota Kinabalu, Sabah, Malaysia | KP337355 |
| *Pseudo-nitzschia delicatissima* | Tasm 10 | Hobart, Tasmania, Australia | AY257848 |
|  | Laeso2 | Laeso, Kattegat, Denmark | DQ329206 |
|  | AL-22 | Italy | DQ813832 |
|  | OFPd972 | Ofunato Bay, Japan | DQ329208 |
|  | ICMB-102 | Catalan coast, Spain | EU478793 |
| *Pseudo-nitzschia dolorosa* | AL-59 | Italy | DQ813835 |
|  | Calif1 | Monterey Bay, California, USA | DQ336152 |
| *Pseudo-nitzschia fraudulenta* | AR3 | Arenys, Cataluña, Spain | DQ990366 |
|  | F10 | - | EU523102 |
|  | Limens1 | Limens, Spain | AY257840 |
| *Pseudo-nitzschia fryxelliana* | NWFSC 241 | Washington State, USA | JN050288 |
|  | NWFSC 242 | Washington State, USA | JN050287 |
| *Pseudo-nitzschia fukuyoi* | PnKk36 | Kota Kinabalu, Sabah, Malaysia | KC147515 |
|  | PnMi158 | Marina Bay, Miri, Sarawak, Malaysia | KR021317 |
|  | PnTb25 | Teluk Batik, Perak, Malaysia | KC147516 |
|  | PnTb31 | Teluk Batik, Perak, Malaysia | KC147517 |
| *Pseudo-nitzschia galaxiae* | ICMB-173 | P. Olimpic, Catalunya, Spain | EU327368 |
|  | (10)4A3 | Greece | JF714915 |
|  | Mex3 | Near Tuxpam, Mexico | AY257850 |
|  | Sydney4 | Australia | EU327370 |
| *Pseudo-nitzschia granii* | UBC100 | Ocean Station Papa | EU068676 |
| *Pseudo-nitzschia hallegraeffi* | CTD44_2 | East Coast of Australia (-32.465 ̊N,153.705 ̊S) | MF044023 |
|  | CTD49_1 | - | MF044021 |
| *Pseudo-nitzschia hasleana* | HAWK3/1 | - | KC017450 |
|  | NWFSC 186 | Coastal WA, NE Pacific Ocean | JN050282 |
|  | NWFSC 252 | Coastal WA, NE Pacific Ocean | JN085962 |
|  | OFP41014 | Ofunato Bay, Iwate, Japan | JN050286 |
| *Pseudo-nitzschia inflatula* | no7 | Phuket, Thailand | DQ329204 |
| *Pseudo-nitzschia kodamae* | PnPd26 | Port Dickson, Negeri Sembilan, Malaysia | KF482050 |
|  | PnPd31 | Port Dickson, Negeri Sembilan, Malaysia | KF482051 |
| *Pseudo-nitzschia limii* | PnMi06 | Rait, Miri, Sarawak, Malaysia | KR021313 |
|  | PnMi16 | Rait, Miri, Sarawak, Malaysia | KR021311 |
| *Pseudo-nitzschia lineola* | NWFSC 188 | Coastal WA, NE Pacific Ocean | JN050284 |
| *Pseudo-nitzschia lundholmiae* | PnMi01 | Marina Bay, Miri, Sarawak, Malaysia | KR021315 |
|  | PnMi28 | Krokop, Miri, Sarawak, Malaysia | KR021316 |
|  | PnTb10 | Teluk Batik, Perak, Malaysia | KC147523 |
| *Pseudo-nitzschia mannii* | AL-101 | Gulf of Naples, Italy | DQ813839 |
|  | (08)10A2 | Greece | JF714905 |
|  | CBA56 | Pesaro, Adriatic Sea, Italy | HE650977 |
|  | CBA60 | Pesaro, Adriatic Sea, Italy | HE650978 |
| *Pseudo-nitzschia micropora* | PnKk14 | Kota Kinabalu, Sabah, Malaysia | JN252422 |
|  | VPB-B3 | Van Phong Bay, Vietnam | AY257847 |
| *Pseudo-nitzschia multiseries* | mu3 | Monterey Bay, California, USA | AY257844 |
|  | OFPm984 | Ofunato Bay, Japan | DQ062664 |
| *Pseudo-nitzschia multistriata* | KoreaA | Chin Hae Bay, South Korea | AY257843 |
|  | B5 | Gulf of Naples, Italy | EF636677 |
| *Pseudo-nitzschia nanaoensis* | MC4188 | Nan’ao Island, China | MG787881 |
|  | MC4206 | Nan’ao Island, China | MG787879 |
|  | MC4213 | Nan’ao Island, China | MG787882 |
|  | MC4215 | Nan’ao Island, China | MG787880 |
| *Pseudo-nitzschia obtusa* | T5 | Tromso, Norway | DK062667 |
| *Pseudo-nitzschia plurisecta* | Hobart 5 | Tasmania, Australia | AY257851 |
|  | 1 MLR-2013 | Gulf of Naples, Italy | AY519273 |
| *Pseudo-nitzschia pseudodelicatissima* | AL–15 | Gulf of Naples, Italy | DQ813826 |
|  | AL-19 | Gulf of Naples, Italy | DQ813828 |
|  | Ner-D5 | Nervion River Estuary, Bay of Biscay, Northern Spain | GQ228392 |
|  | 8A10 | Thermaikos Gulf, Greece | FJ859046 |
|  | 8A13 | Thermaikos Gulf, Greece | FJ859048 |
|  | (09)7A4 | Thermaikos Gulf, Greece | JF714917 |
|  | (09)9A1 | Thermaikos Gulf, Greece | JF714918 |
| *Pseudo-nitzschia pungens* | PnMt45 | MuaraTebas, Sarawak, Malaysia | HQ111412 |
|  | PnSb44 | Santubong, Sarawak, Malaysia | HQ111413 |
| *Pseudo-nitzschia sabit* | PnPd57 | Port Dickson, Negeri Sembilan, Malaysia | KM400610 |
|  | PnPd68 | Port Dickson, Negeri Sembilan, Malaysia | KM400604 |
|  | Ps102 | Manzanillo Bay, Colima Mexico | KP288506 |
|  | Ps147 | Manzanillo Bay, Colima Mexico | KP288507 |
| *Pseudo-nitzschia seriata* | Nissum3 | Nissum Bredning, Denmark | AY257841 |
|  | PLYSt52B | Scotland, UK | AY452524 |
| *Pseudo-nitzschia simulans* | MC281 | Daya Bay, South China Sea | MF374769 |
|  | MC282 | Qingdao, Yellow Sea | MF374770 |
|  | MC940 | Wanshan Island, South China Sea | MF374771 |
|  | MC984 | Taiwan Strait, East China Sea | MF374772 |
| *Pseudo-nitzschia subcurvata* | 1-F | Ross Sea, Antarctica | DQ329205 |
| *Pseudo-nitzschia subfraudulenta* | PnMi71 | Krokop, Miri, Sarawak, Malaysia | KR021299 |
|  | PnMi170 | Marina Bay, Miri, Sarawak, Malaysia | KR021298 |
|  | (08)8A3 | Greece | JF714929 |
| *Pseudo-nitzschia subpacifica* | Nezen | France | EU523104 |
|  | HY32E1 | Korea | LC194952 |
|  | BC26 CL13 10 | France | KM245509 |
|  | Pn252-07E7 | Gulf of Maine, Northwestern Atlantic Ocean | KF006831 |
|  | (09) 2A5 | Greece | JF714906 |
|  | Ps 290 | Todo los Santos Bay, Ensenada | MK706532 |
|  | Ps 291 | Todo los Santos Bay, Ensenada | MK706533 |
|  | Ps 272 | Manzanillo Bay | MK706531 |
|  | Ps 275 | Manzanillo Bay | MK706530 |
| *Pseudo-nitzschia cf subpacifica* | RdA8 | Ria de Arousa, Spain | AY257860 |
|  | P-28 | Costa Nova, Portugal | AY257858 |
|  | Limens8 | Limens, Spain | AY257859 |
|  | Ner-1D | Southern Bay of Biscay, Spain | KC409107 |
| *Pseudo-nitzschia turgidula* | NWFSC 220 | - | JN091764 |
| *Pseudo-nitzschia turgiduloides* | 3-19 | Ross Sea, Antarctica | AY257839 |
