# Supplementary material for: Phylogenetic relationships of Pseudo-nitzschia subpacifica (Bacillariophyceae) from the Mexican Pacific, and its production of domoic acid in culture
Source: PLoS One. 2020 Apr 24;15(4):e0231902. doi: 10.1371/journal.pone.0231902 (PMC7182257; doi:10.1371/journal.pone.0231902)
Supplement: S2 Table — (DOCX) [file pone.0231902.s003.docx]

>08_10A2

--D-DD-A-D-LCR-GCGCLG--G----------L-G-R------LE-A---I--D----I--------A-------A------AAAAAN-----K----Q--N-----Q--Q-H-----H-I----K-HQKHHIIE-DI-I---IGG---CIIIGRCGGL----GG--LIAGLIGCL----D-I-AEIDID--INH-KDHQDHQKH-Q---K-H-KQIIIHQKI-AAA-I--L--C-----L-R----D--AI-C-RC--I--EI-ADHKH-DA-----K--NHNID-L---R-GR-A-AL-G-IDAL-GGI-I--------EI-I----D-L----G--L-D--L--GA--A-IG--R---CGGCE-I-LG-AC--R-D-I-CLC--RIDIAICLCLGG----IR------G--D----I------I---E--I--Q----K-------Q--K-H---HNHII-E-A-AA-D-KHNHI--I--K-H--DK--H--I--I--A-H--Q---K-HK-K-I-E-D-Q-N-A-N-QHI--I----D-QQ------H--E-I-QNEI--E-K--Q-K-NDE--AIDLG--GG-L----R-G---L-------G-------G--------------I------E----D--D-------------A---Q---QN-Q--K-NKQ-KN-DADA-----

>PnKk14

--D-DD-A-D-LCR-GCRCLG--G----------L-C-L------R--R---I-------D----------------I------K----------K-N--H--N-----Q--K-N-----H-K----A-HQKHHIIE-DI-I---IGG---CDIIGR---C-I--GC--C------I-----D-I-----------H---------H-Q---I-H-KQIDIHQKI-AAA-I--I--D-----L-R----C--A--ADA---D--EI-ADHKH-DA-----I--RGRLD-L---R-GR-A-AR-G-I-CL-L-R----------G--D----D-I----G--L----L-DII--A-GG--R---CGGCE-I-LG-RC--R-I-I-CLC--RIDIAICLCLGG----IR------G--D----I------I---E--I--Q----K-------Q--K-H---HNHII-D-A--D-K--HNHI--I--K-H--KK--N--I--D--A-H--Q---K-HK-K-Q-E-A-AAN---N-Q-I--A------QK------H--N-H-IQKA--I-DK-Q-K-N-A--HKQKR--G--C----IIG---C----R--G--------------------I-I------I-------A-----------------Q---KH-Q--I-DHQ-KA-IDIA-----

>PLYSt52B

--D-DD-A-D-LCR-GCGCLG--G----------L-----------------I------------------------I------A---------------E--H-----Q--Q-H-----H-K----D-HQKHHIIE-II-I---IGG---CDIIGR---C-R--GC--L------R-----D-I--A---E--K-N---------H-Q---K-H-KQIDIHQKI-AAE-I--I--D-----L-R----C-GE--I-AI--D--EI-AQHKH-DA-----I--RGRLL-L---R-GL---I--R-C-CL-C-R----------RD-C----IEI-D--L--L-E--L---C--R-AG--R---CGGCE-I-LG-RC--R-D-L-CLL--GIDIAICLCLGG----IAED--LLG--L-R--I------I-I-AI-K-NQNNA-IA-----IQ--K-H---HNHII-I-E--I-K--NNHN--I--K-H--KQ--N--IE-IIDA-H--Q---K-HK-K-K---H-N-N---N-D-I--I----I-ID------H-AK-K-HNHI--HKDN-Q-K-N-H--NKQKR--G--C---LR-G--CL----R-LR-------C------------I-I------I----D--D------I----H-K---NIK-NH-Q--K-NHQ-KA-IDDA-----

>MC281

--D-DD-D-D-LCR-GCGCLG--G----------L-I-I------A--I---I-------D----------------I------A------------A-----H-----Q--K-N-----H-Q----A-HQKHHIIE-DI-I---IGG---CIIIGR---C-----C--R-----DA-----D-I--I---E--I-E---------A-K---H-H-KQIIIHQKI-AAA-I--I--D-----L-R----C--A----ADA-D--EI-ADHKH-DA-----I--RGRLI-I---G-GR-A-AR-G-L-DI-L-G----------G--C----C-I----G--L----C-I-E--A-RRE-R---CGGCG-L-LG-RC--R-I-I-CLC--RIDIAICLCIGG----LR------GC-I----IA--A-DI---H--I--Q----K-------N--K-K-IIHNHII-D-I-II-K--HNHI--I--K-H--KK-HH--Q--I--A-H--Q---K-HKEK-K-E-A-AIH---N-Q-H--H------KK------N--I-I-NQKE--D-KK-Q-I---I--HKQKR--G--C----IIG---C----R--G--------------------D-I------D----I--I-D-E--E---AE-I---Q---KH-Q--I-IHQ-KA-IDDA-----

>MC282

--D-DD-D-D-LCR-GCGCLG--G----------L-I-I------A--I---I-------D----------------I------A------------A-----H-----Q--K-N-----H-Q----A-HQKHHIIE-DI-I---IGG---CIIIGR---C-----C--R-----DA-----D-I--I---E--I-E---------A-K---H-H-KQIIIHQKI-AAA-I--I--D-----L-R----C--A----ADA-D--EI-ADHKH-DA-----I--RGRLI-I---G-GR-A-AR-G-L-DI-L-G----------G--C----C-I----G--L----C-I-E--A-RRE-R---CGGCG-L-LG-RC--R-I-I-CLC--RIDIAICLCIGG----LR------GC-I----IA--A-DI---H--I--Q----K-------N--K-K-IIHNHII-D-I-II-K--HNHI--I--K-H--KK-HH--Q--I--A-H--Q---K-HKEK-K-E-A-AIH---N-Q-H--H------KK------N--I-I-NQKE--D-KK-Q-I---I--HKQKR--G--C----IIG---C----R--G--------------------D-I------D----I--I-D-E--E---AE-I---Q---KH-Q--I-IHQ-KA-IDDA-----

>MC940

--D-DD-D-D-LCR-GCGCLG--G----------L-I-I------A--I---I-------D----------------I------A------------A-----H-----Q--K-N-----H-Q----A-HQKHHIIE-DI-I---IGG---CIIIGR---C-----C--R-----DA-----D-I--I---E--I-E---------A-K---H-H-KQIIIHQKI-AAA-I--I--D-----L-R----C--A----ADA-D--EI-ADHKH-DA-----I--RGRLI-I---G-GR-A-AR-G-L-DI-L-G----------G--C----C-I----G--L----C-I-E--A-RRE-R---CGGCG-L-LG-RC--R-I-I-CLC--RIDIAICLCIGG----LR------GC-I----IA--A-DI---H--I--Q----K-------N--K-K-IIHNHII-D-I-II-K--HNHI--I--K-H--KK-HH--Q--I--A-H--Q---K-HKEK-K-E-A-AIH---N-Q-H--H------KK------N--I-I-NQKE--D-KK-Q-I---I--HKQKR--G--C----IIG---C----R--G--------------------D-I------D----I--I-D-E--E---AE-I---Q---KH-Q--I-IHQ-KA-IDDA-----

>MC984

--D-DD-D-D-LCR-GCGCLG--G----------L-A-I------A--I---I-------D----------------I------E----------I-A--I--H-----Q--K-H-----H-Q----A-HQKHHIIE-DI-I---IGG---CIIIGR---C-----C--R-----IA-----D-I--A---E--D-E---------A-K---H-H-KQIIIHQKI-AAA-I--I--D-----L-R----C--A----ADA-D--EI-ADHKH-DA-----I--RGRLI-I---G-GR-A-AR-G-L-DI-L-G----------G--C----E-I----G--L----C-I-E--AARRE-R---CGGCG-L-LG-RC--R-I-I-CLC--RIDIAICLCIGG----LR------GC-I----IA--A-DI---H--I--Q----K-------N--K-K-IIHNHII-D-I-II-K--HNHI--I--K-H--KK-HH--K--I--A-H--Q---K-HKEK-K-E-E-AIH---N-K-E--H------QK------N--I-I-NQKE--D-KK-Q-I---I--HKQKR--G--C----IIG---C----R--G--------------------D-I------D----I--I-D-E--E---AE-A---Q---KH-Q--I-IHQ-KA-IDDA-----

>1F

--D-DD-A-D-LCR-GCGCLR--G----------L-C-L------R--I---I-------D----------------I------E----------K-N--H--N-----Q--K-H-----H-D----K-HQKHHIIE-AI-I---IGG---CIIIGR---C-I---G--C------------I-I--D------I-E---------H-Q---I-H-KQIDDHQKI-AAE-I--L--C-----L-R----D--A----ACA-D--EI-ADE-H-DA-----K--NHNID-L---R-GR-R-RR-G-I-DA-C-G----------I--D----D-L----G--L------D-EGRA-GR--R---AGGCA-I-LG-RC--R-I-I-CLC--RIDIAICLCLEG----LR------G--D----I------I---E--I--Q----K-------N--K-A-AHHNHII-A-A-AD-K--HNHI--I--K-H--KK--N--I--I--A-H--Q---Q-EK-K-Q-D-K-QAA---N-Q-ND-I------QH------A--E-I-QKKK--D-K--Q-K-H-I--AIDLR--G--C----IIG---C----R--G--------------------D-I------D-------------------E-I---Q---KH-Q--I-DHQ-KN-IAIA-----

>PnMi71

--D-DD-A-D-LCR-GCGCLR--G----------L-C-L------R--C---I-------I----------------I------H----------K-N--HN-I-----K--K-H-----H-D----K-HQKHHIIE-DI-I---IGG---CIIIGR---C-I--GC--L------E-----D-I--I------I-E---DN----H-Q---I-H-KQIDIHQKI-AAA-I--L--C-----L-R----D--A----ACR-D--EI-ADEKH-DA-----K--NHNIE-L---R-GR-A-RA-G-I-CL-C-G----------E--D----I-I----G--L-D--L--GA--A-AG--R---CGGCE-I-IG-RC--R-C-I-CLL--GLDIAICLCLGG----IR------G--D----ILGLRLED---AAHK-NQN---I------IQKQK-H---HNHII-I-A-NQ-I--NNHI--H--K-H--KQ-IA--I--I--A-H--Q---K-HK-K-I-E-D-QNA---N-Q-I--A------AQ------H--N-H-KDKE--I-DK-Q-K-N-E--AIDLG--G--CC---LIG---C----R--G-------L---------E--D-A------A----E--A------N------Q---D---KH-Q--N-HHQ-KN-IDDA-----

>VPBB3

--D-DD-A-D-LCR-GCRCLG--G----------L-C-L------R--R---I-------D----------------I------K----------K-N--H--N-----Q--K-N-----H-K----A-HQKHHIIE-DI-I---IGG---CDIIGR---C-I--GC--C------I-----D-I-----------H---------H-Q---I-H-KQIDIHQKI-AAA-I--I--D-----L-R----C--A--ADA---D--EI-ADHKH-DA-----I--RGRLD-L---R-GR-A-AR-G-I-CL-L-R----------G--D----D-I----G--L----L-DII--A-GG--R---CGGCE-I-LG-RC--R-I-I-CLC--RIDIAICLCLGG----IR------G--D----I------I---E--I--Q----K-------Q--K-H---HNHII-D-A--D-K--HNHI--I--K-H--KK--N--I--D--A-H--Q---K-HK-K-Q-E-A-AAN---N-Q-I--A------QK------H--N-H-IQKA--I-DK-Q-K-N-A--HKQKR--G--C----IIG---C----R--G--------------------I-I------I-------A-----------------Q---KH-Q--I-DHQ-KA-IDIA-----

>PnMi170

--D-DD-A-D-LCR-GCGCLR--G----------L-C-L------R--C---I-------I----------------I------H----------K-N--HN-I-----K--K-H-----H-D----K-HQKHHIIE-DI-I---IGG---CIIIGR---C-I--GC--L------E-----D-I--I------I-E---DN----H-Q---I-H-KQIDIHQKI-AAA-I--L--C-----L-R----D--A----ACR-D--EI-ADEKH-DA-----K--NHNIE-L---R-GR-A-RA-G-I-CL-C-G----------E--D----I-I----G--L-D--L--GA--A-AG--R---CGGCE-I-IG-RC--R-C-I-CLL--GLDIAICLCLGG----IR------G--D----ILGLRLED---EAHK-NQN---I------IQKQK-H---HNHII-I-A-NQ-I--NNHI--H--K-H--KQ-IA--I--I--A-H--Q---K-HK-K-I-E-D-QNA---N-Q-I--A------AQ------H--N-H-KDKE--I-DK-Q-K-N-E--AIDLG--G--CC---LIG---C----R--G-------L---------E--D-A------A----E--A------N------Q---D---KH-Q--N-HHQ-KN-IDDA-----

>088A3

--D-DD-A-D-LCR-GCGCLR--G----------L-C-L------R--C---I-------I----------------I------H----------K-N--HN-I-----K--K-H-----H-D----K-HQKHHIIE-DI-I---IGG---CIIIGR---C-I--GC--L------E-----D-I--I------I-E---IN----H-Q---I-H-KQIDIHQKI-AAA-I--L--C-----L-R----D--A----ACR-D--EI-ADEKH-DA-----K--NHNIE-L---R-GR-A-RA-G-I-CL-C-G----------E--D----I-I----G--L-D--L--GA--A-AG--R---CGGCE-I-IG-RC--R-C-I-CLL--GLDIAICLCLGG----IR------G--D----ILGLRLED---AAHK-NQN---I------IQKQK-H---HNHII-I-A-NQ-I--NNHI--H--K-H--KQ-IA--I--I--A-H--Q---K-HK-K-I-E-D-QNA---N-Q-I--A------AQ------H--N-H-KDKE--I-DK-Q-K-N-E--AIDLG--G--CD---LIG---C----R--G-------L---------E--D-A------A----D--A------N------D---Q---KH-Q--N-EHQ-KN-IDDA-----

>mu3

--D-DD-A-D-LCR-GCGCLG--G----------C-----------------D------------------------I------D----------E----I--H-----Q--K-H-----H-Q----I-HQKHHIIE-II-I---IGG---CIIIGR---CIG--G---C------R-----D-I--A---I--K-H-----------Q---QIH-KQIDIHQKI-AAE-I--I--D-----L-R----CIGL----AI--A--EI-NQHKH-DA-----I--RGACG-L-GGR-R----G--G-C--L-L-G----------AD-CL--GLCI-R--G--L----L---A--R-RG--R---CGGCE-I-IG-RC--R-D-I-CLL--GLDIAICLCLGG----LREA--LLG--L-RCLI------I-I-NH-K-NKHNAAKN-----KQ--I-H---HNHII-I-A--D-I--NKHN--HIIK-H--K---K--II-D--A-H--Q---K-HK-K-K-K-E-A-N---N-Q----KH---N-KH------H-AA-Q-HNHQ--A-QK-KQK-N-K--HDQKR--R--C----R--A--R----C-LG----------------------I------I----I--A-------------Q-------NH----KDKHK-KA-IADA-----

>Nezen

--D-DD-A-D-LCR-GCGCLG--G---L----C-L-G-C------C--D---I-------I----------------I------E-H--H-----K-H--H--N-----K--K-H-----H-K----A-HQKHHIIA-DI-I---IGG---CIIIGR---I----GG--R------------I-I--D------I-A---------K-Q---Q-I-KQIIIHQKI-AAA-I--L--C-----L-R----D--A--L-ACA-I--EI-ADHDH-AA-----K--NHNID-L-GGR-G----GR-G-L-DL-L-G-I--------G--L----D-L----G--I-DC-L---A--R-GG--R---CGGCA-I-IG-GC--R-R-I-CLC--ALCIAICLCLGG----IR------G--R-G--I------I-A-A--K-KQ----K-------Q--K-H---HNHII-I-H-NA-D--HNHI--IK-K-H--QQ--I--I--I--A-H--Q---K-HK-K-K-K-E-N-H-I-I-Q-N--N------QQ------N--N-A-HQKK--K-KI-Q-K-N-EI-EAA-G--GEDC----R-G---L----R--G-------C--------R---E-I------A----A--A------K----H-D---Q---KN-Q--K-HAQ-QA-ADIA-----

>OFPm984

--D-DD-A-D-LCR-GCGCLG--G----------C-----------------D------------------------I------D----------E----I--H-----Q--K-H-----H-Q----I-HQKHHIIE-II-I---IGG---CIIIGR---CIG--G---C------R-----D-I--A---I--K-H-----------Q---QIH-KQIDIHQKI-AAE-I--I--D-----L-R----CIGL----AI--A--EI-NQHKH-DA-----I--RGACG-L-GGR-R----G--G-C--L-L-G----------AD-CL--GLCI-R--G--L----L---A--R-RG--R---CGGCE-I-IG-RC--R-D-I-CLL--GLDIAICLCLGG----LREA--LLG--L-RCLI------I-I-NH-K-NKHNAAKN-----KQ--I-H---HNHII-I-A--D-I--NKHN--HIIK-H--K---K--II-D--A-H--Q---K-HK-K-K-K-E-A-N---N-Q----KH---N-KH------H-AA-Q-HNHQ--A-QK-KQK-N-K--HDQKR--R--C----R--A--R----CALG----------------------I------I----I--A-------------Q-------NH----KDKHK-KA-IADA-----

>KoreaA

--D-DD-A-D-LCR-GCGCLG--G----------L-C-LGGLCLGRL----CI-------A----------------I------A----DHN---KAQ--H--HN----Q--Q-H-----HHKQNHQI-HQKHHIIE-DI-D---IGG---CDIIGR---CIGCLGC--LI-----GLIIIAD-IIIA--DINQI-N---------H-QHHQI-H-KQIDIHQKI-AAE-I--I--D-----L-R----C-GL--D-AD--I--EI-NQHKH-DA-----I--RGRLC-L---R-G----AA-G-C-LL-C-G----------RLGC---GIDI-C--R--C-E--A---A--R--G--R---CGGCE-I-LG-RC----ACL-CLL--GIDIAICLCIGG----LREE--LLG--C-R--I--------D-AE-K-HQNNAAKN-----KQ--I-I---HNHII-I-E--I-K--HNHN--H--A-H--KQ--N--IE-IIEA-H--Q---K-HK-K-K---E-E-A---H-K-H--I----E-KH------K-HK-Q-HNHD--H-QAEQ-K-N-H--HKQKR--G--C---LR-G---L----A--R-------D--------------I------A----D--I-----------E-K---A---N--Q--K-NHQ-KA-IDDA-----

>B5

--D-DD-A-D-LCR-GCGCLG--G----------L-C-LGGLCLGRL----CI-------A----------------I------A----DHN---KAQ--H--HN----Q--Q-H-----HHKQNHQI-HQKHHIIE-DI-D---IGG---CDIIGR---CIGCLGC--LI-----GLIIIAD-IIIA--DINQI-N---------H-QHHQI-H-KQIDIHQKI-AAE-I--I--D-----L-R----C-GL--D-AD--I--EI-NQHKH-DA-----I--RGRLC-L---R-G----AA-G-C-LL-C-G----------RLGC---GIDI-C--R--C-E--A---A--R--G--R---CGGCE-I-LG-RC----ACL-CLL--GIDIAICLCIGG----LREE--LLG--C-R--I--------D-AE-K-HQNNAAKN-----KQ--I-I---HNHII-I-E--I-K--HNHN--H--A-H--KQ--N--IE-IIEA-H--Q---K-HK-K-K---E-E-A---H-K-H--I----E-KH------K-HK-Q-HNHD--H-QAEQ-K-N-H--HKQKR--G--C---LR-G---L----A--R-------D--------------I------A----E--I-----------E-K---A---N--Q--K-NHQ-KA-IDDA-----

>AL15

--D-DD-A-D-LCR-GCRCLG--G----------L-G--------R------I-------I----------------I------D----------K----Q--N-----Q--K-N-----H-K----A-HQKHHIIA-DI-I---IGG---CIIIGR---C----GG--L------C-----I-E-AA---D--I-H---------N-Q---K-H-KQIIIHQKI-AAA-I--L--C-----L-R----D--AIIC-RL-----EI-ADHKH-DA-----K--NHNID-L---R-G--A-RRCG----L-C-I-I--------GG-C----D-D----G--L----L--GR--R-IA--R---CGGLE-D-LG-RC--R---ILCLC--GLDLAICLCIGG----IR------G--C-L--I------I-A-D--H-HQ----K-------I--KQI-I-HNHII-I-ANIE-H-KHNHN--I--K-H--KK--N-----A--A-HQ-Q---I-HK-D-I-K-K-K-H---H-QAA--H------QQ---------I-I-HNKI--H-KKEQ-K-N-A--EIDIR--GC-L----RIG--CL-------G---L---L--------R---A-I-----------K--N-----------N-I---Q---HH-Q--K-NHQ-KA-IDDA-----

>AL19

--D-DD-A-D-LCR-GCRCLG--G----------L-G--------R------I-------I----------------I------D----------K----Q--N-----Q--K-N-----H-K----A-HQKHHIIE-DI-I---IGG---CIIIGR---C----GG--L------C-----I-E-AA---D--I-H---------N-Q---K-H-KQIIIHQKI-AAA-I--L--C-----L-R----D--AIIC-RL-----EI-ADHKH-DA-----K--NHNID-L---R-G--A-RRCG----L-C-I-I--------GG-C----D-D----G--L----L--GR--R-IA--R---CGGLE-D-LG-RC--R---ILCLC--GLDLAICLCIGG----IR------G--C-L--I------I-A-D--H-HQ----K-------I--KQI-I-HNHII-I-ANIE-H-KHNHN--I--K-H--KK--N-----A--A-HQ-Q---I-HK-D-I-K-K-K-H---H-QAA--H------QQ---------I-I-HNKI--H-KKEQ-K-N-A--EIDIR--GC-L----RIG--CL-------G---L---L--------R---A-I------I----K--N-----------N-I---Q---HH-Q--K-NHQ-KA-IDDA-----

>NerD5

--D-DD-A-D-LCR-GCRCLG--G----------L-G--------R------I-------I----------------I------D----------K----Q--N-----Q--K-N-----H-K----A-HQKHHIIA-DI-I---IGG---CIIIGR---C----GG--L------C-----I-E-AA---D--I-H---------N-Q---K-H-KQIIIHQKI-AAA-I--L--C-----L-R----D--AIIC-RL-----EI-ADHKH-DA-----K--NHNID-L---R-G--A-RRCG----L-C-I-I--------GG-C----D-D----G--L----L--GR--R-IA--R---CGGLE-D-LG-RC--R---ILCLC--GLDLAICLCIGG----IR------G--C-L--I------I-A-D--H-HQ----K-------I--KQI-I-HNHII-I-ANIE-H-KHNHN--I--K-H--KK--N-----A--A-HQ-Q---I-HK-D-I-K-K-K-H---H-QAA--H------QQ---------I-I-HNKI--H-KKEQ-K-N-A--EIDIR--GC-L----RIG--CL-------G---L---L--------R---A-I-----------K--N-----------N-I---Q---HH-Q--K-NHQ-KA-IDDA-----

>NerJ2

--DEDD-I-D-L---G-E-L---G---I------L----------E------I------------------------D-----------E-----E----H--------D--Q-H-----E-Q----A-EDIEHIIA-DI-I---IGG---CIIIGR---G----GG--R-----ECGD---I-I-DE---E--K-H----A----K-Q---Q-K-KQIDIHQKI-EAA-IIDLA-C-------R----C-----R--CI----EI-ADHKH-DA-----K--AHHIDDI-G-R-G-----RAE-I-DL-C-G----------GD-C------C----GI-L-I--I--EA--D-GG--R---CGGCD-I-CE-RC--R-R-C-CLC--ALCIAICLCLGE----IAAE---AG-AG-G--E------I-A-AA-A-QQ----KDEAAEADA--K-H---HNHIIEI-H-NA----HHHI--HK-K-H--KA--H--I--E--A-H--Q---K-HK-K-K-E-I-A-EEA-H-Q-H--H----A-KQ------H--N-A-IDDA--K-QK-Q-L-L--------G--GDDI----G-G---C----L--L-----------------------------E----E--A-----------H-----N---HQ-Q--I-DQQ-ENNAEEAA----

>8A10

--D-DD-A-D-LCR-GCRCLG--G----------L-G--------R------I-------I----------------I------D----------K----Q--N-----Q--K-N-----H-K----A-HQKHHIIA-DI-I---IGG---CIIIGR---C----GG--L------C-----I-E-AA---D--I-H---------N-Q---K-H-KQIIIHQKI-AAA-I--L--C-----L-R----D--AIIC-RL-----EI-ADHKH-DA-----K--NHNID-L---R-G--A-RRCG----L-C-I-I--------GG-C----D-D----G--L----L--GR--R-IA--R---CGGLE-D-LG-RC--R---ILCLC--GLDLAICLCIGG----IR------G--C-L--I------I-A-D--H-HQ----K-------I--KQI-I-HNHII-I-ANIE-H-KHNHN--I--K-H--KK--N-----A--A-HQ-Q---I-HK-D-I-K-K-K-H---H-QAA--H------QQ---------I-I-HNKI--H-KKEQ-K-N-A--EIDIR--GC-L----RIG--CL-------G---L---L--------R---A-I------I----K--N-----------N-I---Q---HH-Q--K-NHQ-KA-IDDA-----

>NerJ3

--DEDD-I-D-L---G-E-L---G---I------L----------E------I------------------------D-----------E-----E----H--------D--Q-H-----E-Q----A-EDIEHIIA-DI-I---IGG---CIIIGR---G----GG--R-----ECGD---I-I-DE---E--K-H----A----K-Q---Q-K-KQIDIHQKI-EAA-IIDLA-C-------R----C-----R--CI----EI-ADHKH-DA-----K--AHHIDDI-G-R-G-----RAE-I-DL-C-G----------GD-C------C----GI-L-I--I--EA--D-GG--R---CGGCD-I-CE-RC--R-R-C-CLC--ALCIAICLCLGE----IAAE---AG-AG-G--E------I-A-AA-A-QQ----KDEAAEADA--K-H---HNHIIEI-H-NA----HHHI--HK-K-H--KA--H--I--E--A-H--Q---K-HK-K-K-E-I-A-EEA-H-Q-H--H----A-KQ------H--N-A-IDDA--K-QK-Q-L-L--------G--GDDI----G-G---C----L--L-----------------------------E----E--A-----------H-----N---HQ-Q--I-DQQ-ENNAEEA-----

>0910A3

--D-DD-A-D-LCR-GCRCLG--G----------L-G--------R------I-------I----------------I------D----------K----Q--N-----Q--K-N-----H-K----A-HQKHHIIA-DI-I---IGG---CIIIGR---C----GG--L------C-----I-E-AA---D--I-H---------N-Q---K-H-KQIIIHQKI-AAA-I--L--C-----L-R----D--AIIC-RL-----EI-ADHKH-DA-----K--NHNID-L---R-G--A-RRCG----L-C-I-I--------GG-C----D-D----G--L----L--GR--R-IA--R---CGGLE-D-LG-RC--R---ILCLC--GLDLAICLCIGG----IR------G--C-L--I------I-A-D--H-HQ----K-------I--KQI-I-HNHII-I-ANIE-H-KHNHN--I--K-H--KK--N-----A--A-HQ-Q---I-HK-D-I-K-K-K-H---H-QAA--H------QQ---------I-I-HNKI--H-KKEQ-K-N-A--EIDIR--GC-L----RIG--CL-------G---L---L--------R---A-I------I----K--N-----------N-I---Q---HH-Q--K-NHQ-KA-IDDA-----

>PnMi19

--DEDD-I-D-L---G-E-L---G---I------L----------E------I------------------------D-----------E----------H--------D--Q-H-----A-Q----A-EDIEHIIA-DI-I---IGG---CIIIGR---G----GG--R-----ECGD---I-I-DE---E--K-H----A----K-Q---Q-Q-KQIDIHQKI-EAA-IIDLA-C-------R----C-----R--CI----EI-ADHKH-DA-----K--AHHIDDI-G-R-G-----RAE-I-DL-C-G----------GD-C------C----GI-L-I--I--EA--D-GG--R---CGGCD-I-CE-RC--R-R-C-CLC--ALCIAICLCLGE----IAAE---AG-AG-G--E------I-A-AA-A-QQ----KDEAAEADA--K-H---HNHIIEI-H-NA----HHHI--HK-K-H--KA--H--I--E--A-H--Q---K-HK-K-K-E-I-A-EEA-H-Q-H--H----A-KQ------H--N-A-IDDA--K-QK-Q-L-L--------G--GDDI----G-G---C----L--L-----------------------------E----E--A-----------H-----N---HQ-Q--I-DQQ-ENNAEEAAEEII

>Pnmi168

--DEDD-I-D-L---G-E-L---G---I------L----------E------I------------------------D-----------E----------H--------D--Q-H-----A-Q----A-EDIEHIIA-DI-I---IGG---CIIIGR---G----GG--R-----ECGD---I-I-DE---E--K-H----A----K-Q---Q-Q-KQIDIHQKI-EAA-IIDLA-C-------R----C-----R--CI----EI-ADHKH-DA-----K--AHHIDDI-G-R-G-----RAE-I-DL-C-G----------GD-C------C----GI-L-I--I--EA--D-GG--R---CGGCD-I-CE-RC--R-R-C-CLC--ALCIAICLCLGE----IAAE---AG-AG-G--E------I-A-AA-A-QQ----KDEAAEADA--K-H---HNHIIEI-H-NA----HHHI--HK-K-H--KA--H--I--E--A-H--Q---K-HK-K-K-E-I-A-EEA-H-Q-H--H----A-KQ------H--N-A-IDDA--K-QK-Q-L-L--------G--GDDI----G-G---C----L--L-----------------------------E----E--A-----------H-----N---HQ-Q--I-DQQ-ENNAEEAA----

>HY31E2

--D-DD-A-D-LCR-GCGCLG--G----------L-R-L-------------I-------A----------------D------A----------IN---K--N-----K--Q-N-----H-I----K-HQKHHIIE-II-I---IGG---CIIIGR---C-I--GC---------G-----I-I---------I-Q---------H-Q---I-H-KQIDIHQKD-AAE-I--I--D-----L-R----C-GE--I-AI--D--EI-AQHKH-DA-----I--RGRLG-C---R-GR---AA-E-D--L-C-G----------RD-DL--GIDI-I--G--C-E--A---A--R--G--R---CGGCE-I-LG-GC--RCI---CLL--GIDIAICLCIGG----LRGI---RG--C----A--------D--I-A-HQKID-KK-----NQ--K-I---HNHII-I-A--IIK--HNHI--IH-K-H--QQ--N--IE-IIDA-H--Q---Q-HK-K-K-E-E-A-A---H-Q-I--I----I-IQ------H-AK-Q-HNAI--I-EK-Q-K-H-K--HKQKR--G--CCIALG-G---G----G-LG-------C------------L-I------I-------I------N----H-K---NQQ-AQ-QNDI-HHQ-KA-IDDA-----

>Kervel

--D-DD-A-D-LCR-GCGCLG--G----------L-R-L-------------I-------A----------------D------A----------IN---K--N-----K--Q-N-----H-I----K-HQKHHIIE-II-I---IGG---CIIIGR---C-I--GC---------G-----I-I---------I-Q---------H-Q---I-H-KQIDIHQKD-AAE-I--I--D-----L-R----C-GE--I-AI--D--EI-AQHKH-DA-----I--RGRLG-C---R-GR---AA-E-D--L-C-G----------RD-DL--GIDI-I--G--C-E--A---A--R--G--R---CGGCE-I-LG-GC--RCI---CLL--GIDIAICLCIGG----LRGI---RG--C----A--------D--I-A-HQKID-KK-----NQ--K-I---HNHII-I-A--IIK--HNHI--IH-K-H--QQ--N--IE-IIDA-H--Q---Q-HK-K-K-E-E-A-A---H-Q-I--I----I-IQ------H-AK-Q-HNAI--I-EK-Q-K-H-K--HKQKR--G--CCIALG-G---G----G-LG-------C------------L-I------I-------I------N----H-K---NQQ-AQ-QNDI-HHQ-KA-IDDA-----

>RCC2002

--D-DD-A-D-LCR-GCGCLR--G----------L-C-L------R--I---I-------D----------------I------E----------K-N--H--H-----Q--K-H-----H-D----K-HQKHHIIE-AI-I---IGG---CDIIGR---C-A---G--C------------I-I--D------I-E---------H-Q---I-H-KQIDIHQKI-AAE-I--L--C-----L-R----D--A----ACA-D--EI-ADE-H-DA-----K--NHNID-I---R-GR-R-RR-G-I-DA-C-G----------I--D----D-L----G--L------D-EGRA-GR--R---AGGCA-I-LG-RC--R-I-I-CLC--RIDIAICLCIEG----LR------G--D----I------I---E--I--Q----K-------N--K-I---HNHII-A-A-AD-K--HNHI--I--K-H--KK--N--E--I--A-H--Q---Q-EK-K-Q-D-K-QAA---N-Q-ND-I------QH------A--E-I-QKKK--D-K--Q-K-E-I--AIDLR--G--C----IIG---C----R--G--------------------D-I------D-------------------E-I---Q---KH-Q--I-AHQ-KN-IAIA-----

>RCC2005

--D-DD-A-D-LCR-GCGCLR--G----------L-C-L------R--I---I-------D----------------I------E----------K-N--H--H-----Q--K-H-----H-D----K-HQKHHIIE-AI-I---IGG---CDIIGR---C-A---G--C------------I-I--D------I-E---------H-Q---I-H-KQIDIHQKI-AAE-I--L--C-----L-R----D--A----ACA-D--EI-ADE-H-DA-----K--NHNID-I---R-GR-R-RR-G-I-DA-C-G----------I--D----D-L----G--L------D-EGRA-GR--R---AGGCA-I-LG-RC--R-I-I-CLC--RIDIAICLCIEG----LR------G--D----I------I---E--I--Q----K-------N--K-I---HNHII-A-A-AD-K--HNHI--I--K-H--KK--N--E--I--A-H--Q---Q-EK-K-Q-D-K-QAA---N-Q-ND-I------QH------A--E-I-QKKK--D-K--Q-K-E-I--AIDLR--G--C----IIG---C----R--G--------------------D-I------D-------------------E-I---Q---KH-Q--I-AHQ-KN-IA-------

>AL24

--D-DD-D-D-LCR-GCRCLG--G----------L-G-L------R--G---IGLR----I----------------I---D--E----------K-N--K--I-----Q--K-NKNQQHH-K----A-HQKHHIIE-DI-I---IGG---CDIIGR---C-I--GC--D------I-----D-I-----------E---------H-K---I-H-KQIIIHQKI-AAA-I--L--C-----L-R----D--A----A---CRCEI-ADHKH-DA-----K--NHNID-L---R-GR-A-AR-G-I-CL-L-R----------G--D----D-D----G--L----L-CLE--R-RE--R---CGGCE-I-IE-RC--R-I-I-CLC--RIDIAICLCLGG----IR------G--D----I------I---E--I--Q----K-------Q--K-H---HNHII-I-I-ID-K--HNHI--I--K-H--KE--A--I--D--A-H--Q---K-HKEK-K-E-A-NHH---N-Q-I--A------QK------H--N-H-IQKE--I-DK-Q-K-N-A--EIDLR--G--C----IIG---CGGLLR--G---L-R-L--------RG--I-A------I----A--K-K----H----KNQKNHQ---KH-Q--I-IHQ-KN-IDDA-----

>ICMB129

--D-DD-D-D-LCR-GCRCLG--G----------L-G-L------R--G---IGLR----I----------------I---D--E----------K-N--K--I-----Q--K-NKNQQHH-K----A-HQKHHIIE-DI-I---IGG---CDIIGR---C-I--GC--D------I-----D-I-----------E---------H-K---I-H-KQIIIHQKI-AAA-I--L--C-----L-R----D--A----A---CRCEI-ADHKH-DA-----K--NHNID-L---R-GR-A-AR-G-I-CL-L-R----------G--D----D-D----G--L----L-CLE--R-RE--R---CGGCE-I-IE-RC--R-I-I-CLC--RIDIAICLCLGG----IR------G--D----I------I---E--I--Q----K-------Q--K-H---HNHII-I-I-ID-K--HNHI--I--K-H--KE--A--I--D--A-H--Q---K-HKEK-K-E-A-NHH---N-Q-I--A------QK------H--N-H-IQKE--I-DK-Q-K-N-A--EIDLR--G--C----IIG---CGGLLR--G---L-R-L--------RG--I-A------I----A--K-K----H----KNQKNHQ---KH-Q--I-IHQ-KN-IDDA-----

>HY32E1

--D-DD-A-D-LCR-GCGCLG--G---L----C-L-G-C------C--D---I-------I----------------I------E-H--H-----K-H--H--N-----K--K-H-----H-K----A-HQKHHIIA-DI-I---IGG---CIIIGR---I----GG--R------------I-I--D------I-E---------K-Q---Q-I-KQIIIHQKI-AAA-I--L--C-----L-R----D--A--L-ACA-I--EI-ADHDH-AA-----K--NHNID-L-GGR-G----GR-G-L-DL-L-G-I--------G--L----D-L----G--I-DC-L---A--R-GG--R---CGGCA-I-IG-GC--R-R-I-CLC--ALCIAICLCLGG----IR------G--R-G--I------I-A-A--K-KQ----K-------Q--K-H---HNHII-I-H-NA-D--HNHI--IK-K-H--QQ--I--I--I--A-H--Q---K-HK-K-K-K-E-N-H-I-I-Q-N--N------QQ------N--N-A-HQKK--K-KI-Q-K-N-EI-EAA-G--GEDC----R-G---L----R--G-------C--------R---E-I------A----A--A------K----H-D---Q---KN-Q--K-HAQ-QA-ADIA-----

>BC26CL1310

--D-DD-A-D-LCR-GCGCLG--G---L----C-L-G-C------C--D---I-------I----------------I------E-H--H-----K-H--H--N-----K--K-H-----H-K----A-HQKHHIIA-DI-I---IGG---CIIIGR---I----GG--R------------I-I--D------I-A---------K-Q---Q-I-KQIIIHQKI-AAA-I--L--C-----L-R----D--A--L-ACA-I--EI-ADHDH-AA-----K--NHNID-L-GGR-G----GR-G-L-DL-L-G-I--------G--L----D-L----G--I-DC-L---A--R-GG--R---CGGCA-I-IG-GC--R-R-I-CLC--ALCIAICLCLGG----IR------G--R-G--I------I-A-A--Q-KQ----K-------Q--K-H---HNHII-I-H-NA-D--HNHI--IK-K-H--QQ--I--I--I--A-H--Q---K-HK-K-K-K-E-N-H-I-I-Q-N--N------QQ------N--N-A-HQKK--K-KI-Q-K-N-EI-EAA-G--GEDC----R-G---L----R--G-------C--------R---E-I------A----A--A------K----H-D---Q---KN-Q--K-HAQ-QA-ADIA-----

>33

-DD-DD-A-D-LCR-GCGCLR--G---D------L---L------L--D---I-------I----------------IDH----N----H-----A-AA-I--AI----Q--K-N-----H-Q----A-HQKHH--A-DI-D---IGR---CRGIGR---C-----L--G------------IRI-AI------K-I----K----I-D---H-H-KQIQKHKQI-AAE-IA-I--A-----LIC---ED--D----A---D--EA-EAHIH-EI-----IEARCRC--L-R-R-G----LR------L---G----------G--C------L----IA-L----L--LE--A--G--CIARCLRD--IGLG-LDAAR-C-A-CLC--RLCLRICLCLEE----IR------G--C----A------I---I--D-HQ----K------II--A-H---HNHDI-K-NDHN-K--HNHA--H--K-DI-AN--Q-NQ--I--AKH--H---K-AI-H-Q---A-A-N-N-N-D-IN-H------QK------NKNK-A-IKKN--H-KI-H-K-G-A--DII---DG-------R-------------E----------------------A------E----A--A-----------E-----K---KD-A--I-A-D-KA---I------

>Ps290Ensenada1

--D-DD-A-D-LCR-GCGCLG--G---L----C-L-G-C------C--D---I-------I----------------I------E-H--H-----K-H--H--N-----K--K-H-----H-K----A-HQKHHIIA-DI-I---IGG---CIIIGR---I----GG--R------------I-I--D------I-E---------K-Q---Q-I-KQIIIHQKI-AAA-I--L--C-----L-R----D--A--L-ACA-I--EI-ADHDN-AA-----K--NHNID-L-GGR-G----GR-G-L-DL-L-G-I--------G--L----D-L----G--I-DC-L---A--R-GG--R---CGGCA-I-IG-GC--R-R-I-CLC--ALCIAICLCLGG----IR------G--R-G--I------I-A-A--K-KQ----K-------Q--K-H---HNHII-I-H-NA-D--HNHI--IK-K-H--QQ--I--I--I--A-H--Q---K-HK-K-K-K-E-N-H-I-I-Q-N--N------QQ------N--N-A-HQKK--K-KI-Q-K-H-EI-EAA-G--GEDC----R-G---L----R--G-------C--------R---E-I------A----A--A------K----H-D---Q---KN-Q--K-HAQ-QA-ADIA-----

>NLN33

--D-DD-A-D-LCA-GCGCIG--G----------L-EAL------GIED---A-------A----------------IKN----A----H-----K-K--I--HI----QH-QDH-----H-L----L-GCLGE--A-DI-I---IEI---CRGIGC---R-----C--G------------E-I--D------E-E---------I-Q---H-K-HQIQKHIIIEAAA-KHHKHNIGRCGLLGL---LI--A----ADN-N--QNDNQHKQDAIEIIDAIEIGCRRG-LDGCR-GR---CR-R----L-C-GAAARLCLLRCGR-C------G----G--L----G--CEE-A--G--REE-CLLL--CCCC-LC--G-G---CCC--GLCCRICLCLGG----IR------G--C-RG-I------D-E-I--QKHQ----K------IIEKK-H---HNHII-K-H-HN-Q--EAEAD-H--H-H--KQ--H-HI--I--HHHA-HNNNH-AI-K-Q-E-AIA-H-Q-N-Q-EK-H------KKIEIHKNHHHK-K-HHKK--H-KQ-E-K-HKAAHQIKKHQDG--C----R-L---C-------G-------C------------R-A------GGLG-I--I-----------E-D---Q-NQQDKH--Q-HHKHKIEIDIII----

>Ps291Ensenada

--D-DD-A-D-LCR-GCGCLG--G---L----C-L-G-C------C--D---I-------I----------------I------E-H--H-----K-H--H--N-----K--K-H-----H-K----A-HQKHHIIA-DI-I---IGG---CIIIGR---I----GG--R------------I-I--D------I-E---------K-Q---Q-I-KQIIIHQKI-AAA-I--L--C-----L-R----D--A--L-ACA-I--EI-ADHDN-AA-----K--NHNID-L-GGR-G----GR-G-L-DL-L-G-I--------G--L----D-L----G--I-DC-L---A--R-RG--R---CGGCA-I-IG-GC--R-R-I-CLC--ALCIAICLCLGG----IR------G--R-G--I------I-A-A--K-KQ----K-------Q--K-H---HNHII-I-H-NA-D--HNHI--IK-K-H--QQ--I--I--I--A-H--Q---K-HK-K-K-K-E-N-H-I-I-Q-N--N------QQ------N--N-A-HQKK--K-KI-Q-K-H-EI-EAA-G--GEDC----R-G---L----R--G-------C--------R---E-I------A----A--A------K----H-D---Q---KN-Q--K-HAQ-QA-ADIA-----

>MJBA1540

--D-DD-A-D-ICR-GCGCLG--G----------L-GRLL-----G-GC---A-------A----------------IDA----A----H-----K-QN-I--HK----QH-QQH-----H-I----K-HQKHE--A-DI-I---IEI---CRGIGC---R-----L--G------------E-I--D------E-E---------I-Q---H-K-HQIQKHIIEEAAA-IE-I--AG--GRLGL----D--A----A---A--IA-NKHKQ-KIG---RLGLGCRRG-GCGCR-GR---CR-R----L-C-GRA-------CGR-C------G----G--L----G--CEE-A--G--REE-CLLL--CCCC-LC--G-G---CCC--GLCCRICLCLGG----LR------G--CGRG-I------I-I-IDKKKHQ----KH-----II-KK-H---HNHII-K-H-HN-Q--EAEAD-H--H-HI-KK--H-EA--I--HHHH-HNNNH-AE-K-Q-E-EAA-H-Q-N-Q-EK-H------KQ------HEEK-K-HHKK--H-KQ-E-K-HQAAHQKKKHDQH--Q----N-K---Q-------E-------D------------A-A------E----E--I-----------E-I---IGLCLIIE--I-IIDHHN-KDI------

>Ps275Manzanillo

--D-DD-A-D-LCR-GCGCLG--G---L----C-L-G-C------C--I---I-------I----------------I------D-H--H-----K-H--H--N-----K--K-H-----H-K----A-HQKHHIIA-DI-I---IGG---CIIIGR---I----GG--R------------I-I--D------I-E---------K-Q---Q-I-KQIIIHQKI-AAA-I--L--C-----L-R----D--A--L-ACA-I--EI-ADHDH-AA-----K--NHNID-L-GGR-G----GR-G-L-DL-L-G-I--------G--L----D-L----G--I-DC-L---A--R-GG--R---CGGCA-I-IG-GC--R-R-I-CLC--ALCIAICLCLGG----IR------G--R-G--I------I-A-A--K-KQ----K-------Q--K-H---HNHII-I-H-NA-D--HNHI--IK-K-H--QQ--I--I--I--A-H--Q---K-HK-K-K-K-E-N-H-I-I-Q-N--N------QQ------N--N-A-HQKK--K-KI-Q-K-N-EI-EAA-G--GEDC----R-G---L----R--G-------C--------R---E-I------A----A--A------K----H-D---Q---KN-Q--K-HAQ-QA-ADIA-----

>NTB02

--D-DD-A-D-ICR-GCRCLG--G----------L-GRLL-----G-GC---A-------A----------------IIA----A----H-----K-QN-I--HK----QH-QQH-----H-I----K-HQKHE--A-DI-E---IEI---CRGIGC---R-----L--G------------E-I--D------E-E---------I-Q---H-K-HQIQKHIIEEAAA-IE-I--IG--GRLGL----I--A----A---A--IA-NKHKQ-KIG---REGLGCRRG-GCGCR-GR---CR-R----L-C-GRA-------CGR-C------G----G--L----G--CEE-A--G--REE-CLLD--CCCC-LC--G-G---CCC--GLCCRICLCLGG----LR------G--CGRG-I------I-I-IDKKKHQ----KH-----II-KK-H---HNHII-K-H-HN-Q--EAEAD-H--H-HIDKK--H-EA--I--HHHH-HANNH-AE-K-Q-E-EAA-H-Q-N-Q-EK-H------KQ------HEEK-K-HHKK--H-KQ-E-K-HQAAHQQKKHDQH--Q----A-K---Q-------E-------D------------A-A------E----E--I-----------E-I---IGLCLIIE--I-IIDHHN-KDI------

>Ps272Manzanillo

--D-DD-A-D-LCR-GCGCLG--G---L----C-L-G-C------C--I---I-------I----------------I------D-H--H-----K-H--H--N-----K--K-H-----H-K----A-HQKHHIIA-DI-I---IGG---CIIIGR---I----GG--R------------I-I--D------I-E---------K-Q---Q-I-KQIIIHQKI-AAA-I--L--C-----L-R----D--A--L-ACA-I--EI-ADHDH-AA-----K--NHNID-L-GGR-G----GR-G-L-DL-L-G-I--------G--L----D-L----G--I-DC-L---A--R-GG--R---CGGCA-I-IG-GC--R-R-I-CLC--ALCIAICLCLGG----IR------G--R-G--I------I-A-A--K-KQ----K-------Q--K-H---HNHII-I-H-NA-D--HNHI--IK-K-H--QQ--I--I--I--A-H--Q---K-HK-K-K-K-E-N-H-I-I-Q-N--N------QQ------N--N-A-HQKK--K-KI-Q-K-N-EI-EAA-G--GEDC----R-G---L----R--G-------C--------R---E-I------A----A--A------K----H-D---Q---KN-Q--K-HAQ-QA-ADIA-----

>420

--D-DD-A-D-LCR-GCGLLG--G----------L-R-L------R------I-------E----------------I------A-I--I-----K-N--K--N-----Q--Q-N-----N-Q----I-HQKHHIIE-II-I---IGG---CIIIGR---C-I--GC--L------------D-I--A---E--I-N---------H-Q---I-H-KQIDIHQQI-AAEII-DL--R-----C--AAA-D--A----I---I--EI-ADHKH-DA-----I--RGRL--L---DIGR---RRIG----L-LLG----------R--C---CD-I----GIDL----LGIEA--R-RG--R---CGGCG-L-LG-RL--G-L-L-CLL--GIDIAICLCLGGLRGDIL------G--L-RI-I------I---AIIK-NQN---II-KKH-IQ--K-H---HNHII-I-A-II-K--NNHH--N--K-H--KK-NNQAD--I--A-H--Q---K-HK-K-K-K-E-ADI-Q-N-N-I--I------QH------H--K-Q-HNHQ--K-KK-Q-I-A-H--NKQKE--G--C----LIG---L----R--G---LIG-L--------RR--I-I------A----A--D-K----K----NQN---Q---KH-Q--I-NHQ-IA-IDDA-----

>Pn25207E7

--D-DD-A-D-LCR-GCGCLG--G---L----C-L-G-C------C--D---I-------I----------------I------E-H--H-----K-H--H--N-----K--K-H-----H-K----A-HQKHHIIA-DI-I---IGG---CIIIGR---I----GG--R------------I-I--D------I-A---------K-Q---Q-I-KQIIIHQKI-AAA-I--L--C-----L-R----D--A--L-ACA-I--EI-ADHDH-AA-----K--NHNID-L-GGR-G----GR-G-L-DL-L-G-I--------G--L----D-L----G--I-DC-L---A--R-GG--R---CGGCA-I-IG-GC--R-R-I-CLC--ALCIAICLCLGG----IR------G--R-G--I------I-A-A--K-KQ----K-------Q--K-H---HNHII-I-H-NA-D--HNHI--IK-K-H--QQ--I--I--I--A-H--Q---K-HK-K-K-K-E-N-H-I-I-Q-N--N------QQ------N--N-A-HQKK--K-KI-Q-K-N-EI-EAA-G--GEDC----R-G---L----R--G-------C--------R---E-I------A----A--A------K----H-D---Q---KN-Q--K-HAQ-QA-ADIA-----

>MME13C1

--D-DD-A-D-LCR-GCGLLG--G----------L-R-L------R------I-------E----------------I------A-I--I-----K-N--K--N-----Q--Q-N-----N-Q----I-HQKHHIIE-II-I---IGG---CIIIGR---C-I--GC--L------------D-I--A---E--I-N---------H-Q---I-H-KQIDIHQQI-AAEII-DL--R-----C--AAA-D--A----I---I--EI-ADHKH-DA-----I--RGRL--L---DIGR---IRIG----L-LLG----------R--C---CD-I----GIDL----LGIEA--R-RG--R---CGGCG-L-LG-RL--G-L-L-CLL--GIDIAICLCLGGLRGDIL------G--L-RI-I------I---AIIK-NQN---II-KKH-IQ--K-H---HNHII-I-A-II-K--NNHH--N--K-H--KK-NNQAD--I--A-H--Q---K-HK-K-K-K-E-ADI-Q-N-N-I--I------QH------H--K-Q-HNHQ--K-IK-Q-I-A-H--NKQKE--G--C----LIG---L----R--G---LIG-L--------RR--I-I------A----A--D-K----K----NQN---Q---KH-Q--I-NHQ-IA-IDDA-----

>092A5

--D-DD-A-D-LCR-GCGCLG--G---L----C-L-G-C------C--D---I-------I----------------I------E-H--H-----K-H--H--N-----K--K-H-----H-K----A-HQKHHIIA-DI-I---IGG---CIIIGR---I----GG--R------------I-I--D------I-A---------K-Q---Q-I-KQIIIHQKI-AAA-I--L--C-----L-R----D--A--L-ACA-I--EI-ADHDH-AA-----K--NHNID-L-GGR-G----GR-G-L-DL-L-G-I--------G--L----D-L----G--I-DC-L---A--R-GG--R---CGGCA-I-IG-GC--R-R-I-CLC--ALCIAICLCLGG----IR------G--R-G--I------I-A-A--K-KQ----K-------Q--K-H---HNHII-I-H-NA-D--HNHI--IK-K-H--QQ--I--I--I--A-H--Q---K-HK-K-K-K-E-N-H-I-I-Q-N--N------QQ------N--N-A-HQKK--K-KI-Q-K-N-EI-EAA-G--GEDC----R-G---L----R--G-------C--------R---E-I------A----A--A------K----H-D---Q---KN-Q--K-HAQ-QA-ADIA-----

>FcyL

--D-DD-RCI--CR-GCGLLG--R----------L-R-LL-----RLRL---I-------I----------------I------N-K--N-----KHN--K--N-----K--K-H-----N-I----K-HQKHHKIA-II-I---IGG---CIDIGR---C-L--GC--L------------D-I--A---E--I-N---------H-K---N-H-KQIDIHQQI-AAGLI-DL--R-----C-RI-I-D--A----I---K--HK-NDEIH-QA-----I--RGRL--L---CLGR---RR-C----L-LIA----------R--C---RI-I----EIDL----LGIEA--R-RG--R---CGGCG-L-LG-RL--G-L-L-CLL--GIDIAICLCLGGIAEDIL------G--L-RI-I------I---AIIK-NQN---II-ID--IQ--K-H---HNHII-I-A-II-K--NNHH--N--K-H--KK-NNQAD--I--A-H--Q---K-HK-K-K-K-A-A-I-Q-N-N-I--I------IK------H--K-D-NNHD--K-KK-Q-N-H-H--NKQKA--G--C----AII---C----R--G--------------------I-I------I-------I-------------I---Q---KH-I--A-DHQ-IA-IDDA-----

>RdA8

--D-DD-A-D-LCR-GCGCLG--G---L----C-L-G-C------C--D---I-------I----------------I------E-H--H-----K-H--H--N-----K--K-H-----H-K----A-HQKHHIIA-DI-I---IGG---CIIIGR---I----GG--R------------I-I--D------I-A---------K-Q---Q-I-KQIIIHQKI-AAA-I--L--C-----L-R----D--A--L-ACA-I--EI-ADHDH-AA-----K--NHNID-L-GGR-G----GR-G-L-DL-L-G-I--------G--L----D-L----G--I-DC-L---A--R-GG--R---CGGCA-I-IG-GC--R-R-I-CLC--ALCIAICLCLGG----IR------G--R-G--I------I-A-A--K-KQ----K-------Q--K-H---HNHII-I-H-NA-D--HNHI--IK-K-H--QQ--I--I--I--A-H--Q---K-HK-K-K-K-E-N-H-I-I-Q-N--N------QQ------N--N-A-HQKK--K-KI-Q-K-N-EI-EAA-G--GEDC----R-G---L----R--G-------C--------R---E-I------A----A--A------K----H-D---Q---KN-Q--K-HAQ-QA-ADIA-----

>2EF

--D-DD-A-D-LCR-GCGLLG--R----------L-R-LL-----RLRL---I-------I----------------A------N-K--N-----KHN--K--N-----K--K-H-----N-I----K-HQKHHIIA-II-I---IGG---CIDIGR---C-I--GC--I------------D-I--A---E--I-A---------H-K---A-H-KQIDIHQQI-AAE-I--L--C-----L-RDAILD--A----I---I--EI-ADEIH-DA-----K--NHNI--I---CLGR---RR-C----L-LIA----------R--C---RI-I----EIDL----LGIEA--A-RG--R---CGGCG-L-LG-RL--G-L-L-CLL--GIDIAICLCLGGIAEDIL------G--L-RI-I------A---AIIK-NQN---II-ID--IQ--K-H---HNHII-I-A-II-K--NNHH--N--K-H--KK-NNQAD--I--A-H--Q---K-HK-K-K-E-A-A-I-Q-N-N-I--I------IK------H--K-D-NNHD--K-KK-Q-N-H-E--AIDLR--G--C----AII---C----R--G---L-R-L--------R---I-I------D----A--I-KN---K----NAI---Q---KH-I--I-DHQ-KN-IDDA-----

>Limens8

--D-DD-A-D-LCR-GCGCLG--G---L----C-L-G-C------C--D---I-------I----------------I------E-H--H-----K-H--H--N-----K--K-H-----H-K----A-HQKHHIIA-DI-I---IGG---CIIIGR---I----GG--R------------I-I--D------I-A---------K-Q---Q-I-KQIIIHQKI-AAA-I--L--C-----L-R----D--A--L-ACA-I--EI-ADHDH-AA-----K--NHNID-L-GGR-G----GR-G-L-DL-L-G-I--------G--L----D-L----G--I-DC-L---A--R-GG--R---CGGCA-I-IG-GC--R-R-I-CLC--ALCIAICLCLGG----IR------G--R-G--I------I-A-A--K-KQ----K-------Q--K-H---HNHII-I-H-NA-D--HNHI--IK-K-H--QQ--I--I--I--A-H--Q---K-HK-K-K-K-E-N-H-I-I-Q-N--N------QQ------N--N-A-HQKK--K-KI-Q-K-N-EI-EAA-G--GEDC----R-G---L----R--G-------C--------R---E-I------A----A--A------K----H-D---Q---KN-Q--K-HAQ-QA-ADIA-----

>OceanicaB

--D-DD-RCI--CR-GCRLLG--G----------L-L-L------R------D-------I----------------I------E----------K-N--N--N-----Q--K-H-----N-I----K-HQKHHKIA-II-I---IGG---CIDIGR---C-I--GC--L------------I-I--I---A--IIN---------H-Q---I-H-KQIDIHQQI-AAGLI-DL--R-----C-R--A-D--A----I---K--HK-NDEIH-QA-----I--RGRL--L---LDGR---AAAC----L-L-G----------R--CDLGLD-I-----I-G----L--EA--A-RG--R---CGGCG-L-LG-RC--R-L-L-CLL--GIDIAICLCLGGIAEDIL------G--L-RL-D------I---AN-K-NQN---II-ID--IQ--K-H---HNHII-I-A-II-K--NNHN--N--K-H--IKQNNQAI--I--A-H--Q---K-HK-K-K-E-E-A-N-Q-I-A-I--N------QN------H--K-Q-NNHE--I-DK-Q-I-N-H--NKQKA--G--C----IIG---C----R--G--------------------I-I------I----A--I-----------------Q---KH-Q--I-DHQ-IA-IDIA-----

>Fcylsmal

--D-DD-RCI--CR-GCGLLG--G----------L-L-L------R------D-------I----------------I------E----------K-N--N--N-----Q--K-H-----H-D----K-HQKHHKIA-II-I---IGG---CIDIGR---C-I--GC--L------------I-I--I--------N---------H-Q---I-H-KQIDIHQQI-AAGLI-DL--R-----C-R--A-D--A----I---K--HK-NDEIH-QA-----I--RGRL--L---LDGR---AAAC----L-L-G----------R--CDLGLD-I-----I-G----L--EA--A-RG--R---CGGCG-L-LG-RC--R-L-L-CLL--GIDIAICLCLGGIAEDIL------G--L-RL-D------I---AN-K-NQN---II-ID--IQ--K-H---HNHII-I-A-II-K--NNHN--N--K-H--IKQNNQAI--I--A-H--Q---K-HK-K-K-E-E-A-N-Q-I-A-I--N------QN------H--K-Q-NNHE--I-DK-Q-I-N-H--NKQKA--G--C----IIG---C----R--G--------------------I-I------I----A--I-----------------Q---KH-Q--I-DHQ-IA-IDIA-----

>NerD1

--D-DD-D-D-LCR-GCRCLG--G----------L-G-L------R--G---IGLR----I----------------I---D--E----------K-N--K--I-----Q--K-NKNQQHH-K----A-HQKHHIIE-DI-I---IGG---CDIIGR---C-I--GC--D------I-----D-I-----------E---------H-K---I-H-KQIIIHQKI-AAA-I--L--C-----L-R----D--A----A---CRCEI-ADHKH-DA-----K--NHNID-L---R-GR-A-AR-G-I-CL-L-R----------G--D----D-D----G--L----L-CLE--R-RE--R---CGGCE-I-IE-RC--R-I-I-CLC--RIDIAICLCLGG----IR------G--D----I------I---E--I--Q----K-------Q--K-H---HNHII-I-I-ID-K--HNHI--I--K-H--KE--A--I--D--A-H--Q---K-HKEK-K-E-A-NHH---N-Q-I--A------QK------H--N-H-IQKE--I-DK-Q-K-N-A--EIDLR--G--C----IIG---CGGLLR--G---L-R-L--------RG--I-A------I----A--K-K----H----KNQKNHQ---KH-Q--I-IHQ-KN-IDDA-----

>au43

--D-DD-A-D-LCR-GCGCLG--G----------L-----------------I------------------------I------A---------------E--H-----Q--Q-N-----H-K----D-HQKHHIIE-II-I---IGG---CDIIGR---C-R--GC--L------R-----D-I--A---E--K-N---------H-Q---K-H-KQIDIHQKI-AAE-I--I--D-----L-R----C-GE--I-AI--D--EI-AQHKH-DA-----I--RGRLL-L---R-G----AG-R-C-CL-C-R----------RD-D----IEI-D--L--L-E--L---C--R-AG--R---CGGCE-I-LG-RC--R-I-L-CLL--GIDIAICLCLGG----IAED--LLG--L-R--I------I-D-AI-K-NQNNA-IA-----IQ--K-H---HNHII-I-E--I-K--NNHN--I--K-H--KQ--N--IE-IIDA-H--Q---K-HK-K-K---H-N-N---N-D-I--I----I-ID------E-AK-K-HNHD--HKQA-Q-K-N-H--NKQKR--G--C---LR-G--CL----R-LR-------C------------L-I------I----D--I------N----H-K---NDK-NH-Q--K-NHQ-KA-IDDA-----

>PLYST19A

--D-DD-A-D-LCR-GCGCLG--G----------L-----------------I------------------------I------A---------------E--H-----Q--Q-N-----H-K----D-HQKHHIIE-II-I---IGG---CDIIGR---C-R--GC--L------R-----D-I--A---E--K-N---------H-Q---K-H-KQIDIHQKI-AAE-I--I--D-----L-R----C-GE--I-AI--D--EI-AQHKH-DA-----I--RGRLL-L---R-G----AG-R-C-CL-C-R----------RD-D----IEI-D--L--L-E--L---C--R-AG--R---CGGCE-I-LG-RC--R-I-L-CLL--GIDIAICLCLGG----IAED--LLG--L-R--I------I-D-AI-K-NQNNA-IA-----IQ--K-H---HNHII-I-E--I-K--NNHN--I--K-H--KQ--N--IE-IIDA-H--Q---K-HK-K-K---H-N-N---N-D-I--I----I-ID------E-AK-K-HNHD--HKQA-Q-K-N-H--NKQKR--G--C---LR-G--CL----R-LR-------C------------L-I------I----D--I------N----H-K---NDK-NH-Q--K-NHQ-KA-IDDA-----

>Pn12

--D-DD-A-D-LCR-GCGCLG--G----------L-----------------I------------------------I------A---------------E--H-----Q--Q-N-----H-K----D-HQKHHIIE-II-I---IGG---CDIIGR---C-R--GC--L------R-----D-I--A---E--K-N---------H-Q---K-H-KQIDIHQKI-AAE-I--I--D-----L-R----C-GE--I-AI--D--EI-AQHKH-DA-----I--RGRLL-L---R-G----AG-R-C-CL-C-R----------RD-D----IEI-D--L--L-E--L---C--R-AG--R---CGGCE-I-LG-RC--R-I-L-CLL--GIDIAICLCLGG----IAED--LLG--L-R--I------I-D-AI-K-NQNNA-IA-----IQ--K-H---HNHII-I-E--I-K--NNHN--I--K-H--KQ--N--IE-IIDA-H--Q---K-HK-K-K---H-N-N---N-D-I--I----I-ID------E-AK-K-HNHD--HKQA-Q-K-N-H--NKQKR--G--C---LR-G--CL----R-LR-------C------------L-I------I----D--I------N----H-K---NDK-NH-Q--K-NHQ-KA-IDDA-----

>Pn15

--D-DD-A-D-LCR-GCGCLG--G----------L-----------------I------------------------I------A---------------E--H-----Q--Q-N-----H-K----D-HQKHHIIE-II-I---IGG---CDIIGA---C-R--GC--L------R-----D-I--A---E--K-N---------H-Q---K-H-AQIDIHQKI-AAE-I--I--D-----L-R----C-GE--I-AI--D--EI-AQHKH-DA-----I--RGRLL-L---R-G----AG-R-C-CL-C-R----------RD-D----IEI-D--L--L-E--L---C--R-AG--R---CGGCE-I-LG-RC--R-I-L-CLL--GIDIAICLCLGG----IAED--LLG--L-R--I------I-D-AI-K-NQNNA-IA-----IQ--K-H---HNHII-I-E--I-K--NNHN--I--K-H--KQ--N--IE-IIDA-H--Q---K-HK-K-K---H-N-N---N-D-I--I----I-ID------E-AK-K-HNHD--HKQA-Q-K-N-H--NKQKR--G--C---LR-G--CL----R-LR-------C------------L-I------I----D--I------N----H-K---NDK-NH-Q--K-NHQ-KA-IDDA-----

>PnMi32

--DDDD-A-D-LCR-GCRCL---G---G------L----------C------I-------A----------------I-----------H----------H--------Q--Q-H-----H-K----A-HQKHHIIA-DI-I---IGG---CIIIGR---G----GG--G-----GCRD---I-I--E---I--K-H----Q----Q-Q---K-K-KQIIDHQKI-EAA-IIILA-C-----L-R----C-----R--CA----EI-ADHKH-DA-----K--NHNID-I-GGR-G-----RAE-I-DL-L-G----------GA-C------L----G--L-D--I--EA--A-GG--R---CGGCD-I-CG-RL--R-R-C-CLC--ALCIAICLCLGG----IAA----LGRAG-G--R------I-I-AI-K-QQ----KQ-NA-DIQ--K-H---HNHII-I-H-NA----HHHI--HK-K-H--KK--H--I--I--A-H--Q---K-HK-K-K-E-E-A-A-A-H-Q-N--H----A-QQ------H--N-A-IDDA--K-QK-Q-K-I-E--EIDIG--GDII----G-----C----R--G-----------------------------A----I--A-----------I-D---Q---KH-K--I-IHQ-DADAD-A-----

>PnTb19

--DDDD-A-D-LCR-GCRCL---G---G------L----------C------I-------A----------------I-----------H----------H--------Q--Q-H-----H-K----A-HQKHHIIA-DI-I---IGG---CIIIGR---G----GG--G-----GCRD---I-I--E---I--K-H----Q----Q-Q---K-K-KQIIDHQKI-EAA-IIILA-C-----L-R----C-----R--CA----EI-ADHKH-DA-----K--NHNID-I-GGR-G-----RAE-I-DL-L-G----------GA-C------L----G--L-D--I--EA--A-GG--R---CGGCD-I-CG-RL--R-R-C-CLC--ALCIAICLCLGG----IAA----LGRAG-G--R------I-I-AI-K-QQ----KQ-NA-DIQ--K-H---HNHII-I-H-NA----HHHI--HK-K-H--KK--H--I--I--A-H--Q---K-HK-K-K-E-E-A-A-A-H-Q-N--H----A-QQ------H--N-A-IDDA--K-QK-Q-K-I-E--EIDIG--GDII----G-----C----R--G-----------------------------A----I--A-----------I-D---Q---KH-K--I-IHQ-DADAD-A-----

>Pnmi02

--DDDD-A-D-LCR-GCRCL---G---G------L----------C------I-------A----------------I-----------H----------H--------Q--Q-H-----H-K----A-HQKHHIIA-DI-I---IGG---CIIIGR---G----GG--G-----GCRD---I-I--E---I--K-H----Q----Q-Q---K-K-KQIIDHQKI-EAA-IIILA-C-----L-R----C-----R--CA----EI-ADHKH-DA-----K--NHNID-I-GGR-G-----RAE-I-DL-L-G----------GA-C------L----G--L-D--I--EA--A-GG--R---CGGCD-I-CG-RL--R-R-C-CLC--ALCIAICLCLGG----IAA----LGRAG-G--R------I-I-AI-K-QQ----KQ-NA-DIQ--K-H---HNHII-I-H-NA----HHHI--HK-K-H--KK--H--I--I--A-H--Q---K-HK-K-K-E-E-A-A-A-H-Q-N--H----A-QQ------H--N-A-IDDA--K-QK-Q-K-I-E--EIDIG--GDII----G-----C----R--G-----------------------------A----I--A-----------I-D---Q---KH-K--I-IHQ-DADAD-A-----

>PnMi04

--D-DD-A-D-LCR-GCGCLG--G---L----C-L-G-C------C--D---I--D----I----------------IDI----E-H--H-----K-H--H--N-----K--K-H-----H-Q----A-HQKHHIIA-DI-I---IGG---CIIIGR---I----GG--R------------I-I--D------I-E---------K-Q---Q-I-KQIIIHQKI-AAA-I--L--C-----L-R----D--A--L-ACA-I--EI-ADHDH-AA-----K--NHNID-L-GGA-G----GR-G-L-DL-L-G-I--------G--L----D-L----G--I-DC-E---A--R-GG--R---CGGCA-I-IG-GC--R-R-I-CLC--ALCIAICLCLGG----IR------G--R-ED-I------IDI-A--E-KQ----K-------Q--K-H---HNHII-I-H-NA-D--HNHE--IK-K-H--QQ--I--I--I--A-H--Q---K-HK-K-K-K-E-A-H-I-I-Q-N--N------QQ------N--N-A-HQKK--K-DI-Q-K-H-EI-EAA-G--GEDC----R-G---L----R--G-------C--------R---E-I------A----A---------K----H-D---Q---KH-Q--K-HEQ-QA-ADIA-----

>PnMi13

--D-DD-A-D-LCR-GCGCLG--G---L----C-L-G-C------C--D---I--D----I----------------IDI----E-H--H-----K-H--H--N-----K--K-H-----H-Q----A-HQKHHIIA-DI-I---IGG---CIIIGR---I----GG--R------------I-I--D------I-E---------K-Q---Q-I-KQIIIHQKI-AAA-I--L--C-----L-R----D--A--L-ACA-I--EI-ADHDH-AA-----K--NHNID-L-GGA-G----GR-G-L-DL-L-G-I--------G--L----D-L----G--I-DC-E---A--R-GG--R---CGGCA-I-IG-GC--R-R-I-CLC--ALCIAICLCLGG----IR------G--R-ED-I------IDI-A--E-KQ----K-------Q--K-H---HNHII-I-H-NA-D--HNHE--IK-K-H--QQ--I--I--I--A-H--Q---K-HK-K-K-K-E-A-H-I-I-Q-N--N------QQ------N--N-A-HQKK--K-DI-Q-K-H-EI-EAA-G--GEDC----R-G---L----R--G-------C--------R---E-I------A----A---------K----H-D---Q---KH-Q--K-HEQ-QA-ADIA-----

>P28

--D-DD-A-D-LCR-GCGCLG--G---L----C-L-G-C------C--D---I-------I----------------I------E-H--H-----K-H--H--N-----K--K-H-----H-K----A-HQKHHIIA-DI-I---IGG---CIIIGR---I----GG--R------------I-I--D------I-A---------K-Q---Q-I-KQIIIHQKI-AAA-I--L--C-----L-R----D--A--L-ACA-I--EI-ADHDH-AA-----K--NHNID-L-GGR-G----GR-G-L-DL-L-G-I--------G--L----D-L----G--I-DC-L---A--R-GG--R---CGGCA-I-IG-GC--R-R-I-CLC--ALCIAICLCLGG----IR------G--R-G--I------I-A-A--K-KQ----K-------Q--K-H---HNHII-I-H-NA-D--HNHI--IK-K-H--QQ--I--I--I--A-H--Q---K-HK-K-K-K-E-N-H-I-I-Q-N--N------QQ------N--N-A-HQKK--K-KI-Q-K-N-EI-EAA-G--GEDC----R-G---L----R--G-------C--------R---E-I------A----A--A------K----H-D---Q---KN-Q--K-HAQ-QA-ADIA-----

>Ner1D

--D-DD-A-D-LCR-GCGCLG--G---L----C-L-G-C------C--D---I-------I----------------I------E-H--H-----K-H--H--N-----K--K-H-----H-K----A-HQKHHIIA-DI-I---IGG---CIIIGR---I----GG--R------------I-I--D------I-A---------K-Q---Q-I-KQIIIHQKI-AAA-I--L--C-----L-R----D--A--L-ACA-I--EI-ADHDH-AA-----K--NHNID-L-GGR-G----GR-G-L-DL-L-G-I--------G--L----D-L----G--I-DC-L---A--R-GG--R---CGGCA-I-IG-GC--R-R-I-CLC--ALCIAICLCLGG----IR------G--R-G--I------I-A-A--K-KQ----K-------Q--K-H---HNHII-I-H-NA-D--HNHI--IK-K-H--QQ--I--I--I--A-H--Q---K-HK-K-K-K-E-N-H-I-I-Q-N--N------QQ------N--N-A-HQKK--K-KI-Q-K-N-EI-EAA-G--GEDC----R-G---L----R--G-------C--------R---E-I------A----A--A------K----H-D---Q---KN-Q--K-HAQ-QA-ADIA-----

>NWFSC220

--D-DD-A-D-LCR-GCACLG--G----------I-C-L------R--I---I-------DA---------------I------I----------K-N--H--I-----Q--Q-N-----H-I----D-HQKHHIIA-AI-D---IGG---CIIIGR---C-I--GC--D------------D-I-----------E---I-----H-Q---I-H-KQIIIHQKI-AAA-I--L--C-----L-R----D--E----ALR-D--EI-ADEKH-DA-----K--NHNID-L---R-GR-A-AA-G-L-CL-C-L----------E--I----D-L----G--L-D--I--EA--A-AE--R---CGGCE-I-LG-RC--R---IDCLC--RIDLAICLCIEG----IR------G--D----I------I---E--I--Q----K------DQ--I-H----NHII-I-A-ND-K--HNHI--I--K-H--KK--N--I--D--A-H--Q---K-HK-E-I-E-D-DNK---N-D-I--A------AH------H--N-H-NIKD--D-IK-Q-K-N-A--AIDLR--G--C----IIG---C----R--G--------------------I-I------I----I--A-------------D---Q---KH-Q--I-DHQ-KN-IDDA-----

>319

--D-DD-A-D-LCR-GCGCLR--G----------L-C-L------R--I---I-------D----------------I------E----------K-N--H--N-----Q--K-N-----H-D----K-HQKHHIIA-AI-D---IGG---CIIIGR---C-L--GC--I------I-----D-I-----------E---------H-Q---N-H-KQIIIHQKI-AAA-I--L--L-----L-R----D--I----ILR-I--EI-ADEKH-DA-----K--NHNAD-L---R-GR-A-AC-G-L-CL-L-G----------G--D----D-L----G--L-C--I--IA--A-IG--R---CGGCE-I-LG-RC--R-R-I-CLC--GICLAICLCLGG----LR------G--D----I------I---E--I--Q----K------NKA-K-H---HNHII-I-A-NH-K--HNHI--K--K-H--KK--N--I--D--A-H--Q---K-HK-K-I-E-D-HNK---N-D-I--A------QQ------H--N-H-HIKH--D-DK-Q-K-N-E--AIDLR--G--C----LLG---L----R--G--------------------I-D------I-------A-------------I---Q---KN-Q--N-NHQ-KN-IDIA-----

>MC4213

--D-DD-A-D-ICR-GCGCLGELG----------CLRGL------R------E------------------------I------A----------I----K--H-----Q--KNHQN---E-Q--HHIKHQKHEIDEEII-I---IGG---CIIIGRE--C-G--GLIIR------R-----AAI--E---A--K-K---II----N-Q---Q-HEKQIIIHQKI-AAE-I--I--D-----L-R----C-GE--D-AI--I--EI-AQHKH-DA-----I--RGRLG-L-RGR-R----AR-G-D-EL-R-G----------CD-C---GL---C--L--L----L---G--R-RREGR---LGGC--L-CGGRC--R-E-LLCLL--GIDIAICLCLGG----LREA--LLG--C-R--E------I-D-AE-K-HQNNAAKN-----KQ--I-H---HNHII-IIE--I-K--NNHN--H--K-H--KQ--K--DE-IH-N-H--Q---Q-HK-KEK-K-K-Q-H---H-DDI--IEIEDD-NH------H-QH-I-EDHQKNEIQKEKKK-K-HQ-NKQKR--G--C---LR-GIIGG----C-LR----------------------IDIIAA-I----E--I-------------K-------NH-QQIKKNHQ-KA-IDDA-----

>MC4188

--D-DD-A-D-ICR-GCGCLGELG----------CLRGL------R------E------------------------I------A----------I----K--H-----Q--KNHQN---E-Q--HHIKHQKHEIDEEII-I---IGG---CIIIGRE--C-G--GLIIR------R-----AAI--E---A--K-K---II----N-Q---Q-HEKQIIIHQKI-AAE-I--I--D-----L-R----C-GE--D-AI--I--EI-AQHKH-DA-----I--RGRLG-L-RGR-R----AR-G-D-EL-R-G----------CD-C---GL---C--L--L----L---G--R-RREGR---LGGC--L-CGGRC--R-E-LLCLL--GIDIAICLCLGG----LREA--LLG--C-R--E------I-D-AE-K-HQNNAAKN-----KQ--I-H---HNHII-IIE--I-K--NNHN--H--K-H--KQ--K--DE-IH-N-H--Q---Q-HK-KEK-K-K-Q-H---H-DDI--IEIEDD-NH------H-QH-I-EDHQKNEIQKEKKK-K-HQ-NKQKR--G--C---LR-GIIGG----C-LR----------------------IDIIAA-I----E--I-------------K-------NH-QQIKKNHQ-KA-IDDA-----

>MC4215

--D-DD-A-D-ICR-GCGCLGELG----------CLRGL------R------E------------------------I------A----------I----K--H-----Q--KNHQN---E-Q--HHIKHQKHEIDEEII-I---IGG---CIIIGRE--C-G--GLIIR------R-----AAI--E---A--K-K---II----N-Q---Q-HEKQIIIHQKI-AAE-I--I--D-----L-R----C-GE--D-AI--I--EI-AQHKH-DA-----I--RGRLG-L-RGR-R----AR-G-D-EL-R-G----------CD-C---GL---C--L--L----L---G--R-RREGR---LGGC--L-CGGRC--R-E-LLCLL--GIDIAICLCLGG----LREA--LLG--C-R--E------I-D-AE-K-HQNNAAKN-----KQ--I-H---HNHII-IIE--I-K--NNHN--H--K-H--KQ--K--DE-IH-N-H--Q---Q-HK-KEK-K-K-Q-H---H-DDI--IEIEDD-NH------H-QH-I-EDHQKNEIQKEKKK-K-HQ-NKQKR--G--C---LR-GIIGG----C-LR----------------------I-----------E--I-------------K-------NH-QQIKKNHQ-KA-IDDA-----

>MC4206

--D-DD-A-D-ICR-GCGCLGELG----------CLRGL------R------E------------------------I------A----------I----K--H-----Q--KNHQN---E-Q--HHIKHQKHEIDEEII-I---IGG---CIIIGRE--C-G--GLIIR------R-----AAI--E---A--K-K---II----N-Q---Q-HEKQIIIHQKI-AAE-I--I--D-----L-R----C-GE--D-AI--I--EI-AQHKH-DA-----I--RGRLG-L-RGR-R----AR-G-D-EL-R-G----------CD-C---GL---C--L--L----L---G--R-RREGR---LGGC--L-CGGRC--R-E-LLCLL--GIDIAICLCLGG----LREA--LLG--C-R--E------I-D-AE-K-HQNNAAKN-----KQ--I-H---HNHII-IIE--I-K--NNHN--H--K-H--KQ--K--DE-IH-N-H--Q---Q-HK-KEK-K-K-Q-H---H-DDI--IEIEDD-NH------H-QH-I-EDHQKNEIQKEKKK-K-HQ-NKQKR--G--C---LR-GIIGG----C-LR----------------------I-----------E--I-------------K-------NH-QQIKKNHQ-KA-IDDA-----

>CTD44_2

--D-DD-D-DDLCR-GCGCLG--G----------L-L-L------R--I---I------------------------I------D----------K-N--N--N-----Q--K-N-----H-Q----A-HQKHHIIE-DI-I---IGG---CIIIGR---C-----C--R-----DA-----DDI--A---E--I-E---------A-K---H-H-KQIIIHQKI-AAA-I--L--C-----L-R----D--A----ACR-C--EI-ADHKH-DA-----K--NHNII-I---G-GRRA-AR-G-L-DI-L-G----------G--D----D-I----G--L----C-L-E--AARG--R---CGGCE-L-LG-RC--R-I-I-CLC--RIDIAICLCIGG----LR------GC-L----IA--A-IH---H--I--Q----K-------N--K-KDI-HNHII-D-I-II-K--HNHI--I--K-H--KK-HH--I--I--A-H--Q---K-HK-K-K-E-E-AAH-H-N-K-EIEA------QQ------N--D-I-NQKE--D-KK-QKI---I--EIDLR--G--C----IIG---C----R--G---C-L-L------------I-I------D----E---------H---NH-D---Q---KH-Q--I-IHQ-KN-IDDAA----

>NerD6

-ID-DD-A-D-LCR-GCRCLG--G----------L-G--------R------ILRC----I---ILRIIIIAIIIKNI------DHK--ND----K----Q--N-----Q--K-N-----H-K----A-HQKHHIIE-DI-I---IGG---CIIIGR---C----GG--L------C-----I-E-AA---D--I-H---------N-Q---K-H-KQIIIHQKI-AAA-I--L--C-----L-R----D--AIIC-RL-----EI-ADHKH-DA-----K--NHNID-L---R-G--A-ARCG----L-C-I-I--------GG-C----D-D----G--L----L--GR--R-IA--R---CGGLE-D-LG-GC--R---ILCLC--GLDLAICLCIGG----IR------G--C-L--I------I-A-E--H-HQ----K-------I--KQI-I-HNHII-I-ENIE-H-KHNHN--I--K-H--KK--N-----A--A-HQ-Q---I-HK-D-I-K-K-K-H---H-QAA--H------QQ---------I-I-HNKI--H-KEEQ-K-N-A--AIDIR--GC-L----RIG--CL-------G---L---L-R------RI--E-I------IAAAKK--N-----------N-D---Q---HH-Q--K-NHQ-KA-IDAID----

>PnMi18

--D-DD-A-D-LCR-GCGCLG--G---L----C-L-G-C------C--D---I--D----I----------------IDI----E-H--H-----K-H--H--N-----K--K-H-----H-Q----A-HQKHHIIA-DI-I---IGG---CIIIGR---I----GG--R------------I-I--D------I-E---------K-Q---Q-I-KQIIIHQKI-AAA-I--L--C-----L-R----D--A--L-ACA-I--EI-ADHDH-AA-----K--NHNID-L-GGA-G----GR-G-L-DL-L-G-I--------G--L----D-L----G--I-DC-E---A--R-GG--R---CGGCA-I-IG-GC--R-R-I-CLC--ALCIAICLCLGG----IR------G--R-ED-I------IDI-A--E-KQ----K-------Q--K-H---HNHII-I-H-NA-D--HNHE--IK-K-H--QQ--I--I--I--A-H--Q---K-HK-K-K-K-E-A-H-I-I-Q-N--N------QQ------N--N-A-HQKK--K-DI-Q-K-H-EI-EAA-G--GEDC----R-G---L----R--G-------C--------R---E-I------A----A---------K----H-D---Q---KH-Q--K-HEQ-QA-ADIA-----

>PnSm07

--D-DD-A-D-LCR-GCGCLGILG----------C-C---------------I------------------------I------I----DI----III--H--H-----QNIQ-H-----H-Q----I-HQKHHIIE-II-I---IGG---CIIIGR---C-C--GC---------------D-I--D---E-II-I---------H-Q---H-H-KQIDIHQKD-AAE-I--I--D-----L-R----C-GE--IIAD--D--EI-AQHKH-DA-----I--RGRLC-L---R-G----AA-C-C-CL-C-A----------RC-C----IEI-DIIG--C----L---C--R-RG--R---CGGCG-L-LG-RC--R-C-L-CLL--GIDIAICLCLGG----IRGRRGLLG----G--D------I-D-EI-I-QQHNKKKKKI---IQ--K-N---HNHII-I-E--IIK--NHHN--H--KDH--KQ--N--IHQIAAA-H--Q---Q-HK-K-K-DKH-I-N---H-Q-I--I----IEII------H-HK-E-HNHD--H-HADQ-K-N-H--HKQKR--G--C---LR-L---G----G-CR-------L------------E-I------A----N--K-----------H-K-------QH----K-HHQ-KA-IDDA-----

>PnKk31

--D-DD-A-D-LCR-GCGCLGILG----------C-C---------------I------------------------I------I----DI----III--H--H-----QNIQ-H-----H-Q----I-HQKHHIIE-II-I---IGG---CIIIGR---C-C--GC---------------D-I--D---E-II-I---------H-Q---H-H-KQIDIHQKD-AAE-I--I--D-----L-R----C-GE--IIAD--D--EI-AQHKH-DA-----I--RGRLC-L---R-G----AA-C-C-CL-C-A----------RC-C----IEI-DIIG--C----L---C--R-RG--R---CGGCG-L-LG-RC--R-C-L-CLL--GIDIAICLCLGG----IRGRRGLLG----G--D------I-D-EI-I-QQHNKKKKKI---IQ--K-N---HNHII-I-E--IIK--NHHN--H--KDH--KQ--N--IHQIAAA-H--Q---Q-HK-K-K-DKH-I-N---H-Q-I--I----IEII------H-HK-E-HNHD--H-HAEQ-K-N-H--HKQKR--A--C---LR-L--EG----G-CR-------L------------E-I------A----N--K-----------H-K-------QH----K-HHD-KA-IDDA-----

>L26

--D-DD-D-DELCR-GCGCLG--G----------L-L-L------R--D---I-------D----------------I------E----------K-N--N--H-----Q--K-N-----H-Q----A-HQKHHIIE-DI-I---IGG---CIDIGR---C-----CGLR------R-----D-I--A---E--I-K---K-----N-K---H-H-KQIDIHQKI-AAA-I--I--D-----L-R----C--A----ADA-D--EI-ADHKH-DA-----I--RGRLI-I---G-GR-A-AR-G-L-DI-L-G----------G--D----D-I----G--L----C-IIE--A-RG--R---CGGCE-I-CG-RC--R-I-I-CLC--RIDIAICLCIGG----LA------GD-I----II--A-II---E--I-------K-------N--K-K-I-HNHII-D-I-II-K--HNHI--I--K-H--KK-EH--I--I--A-H--Q---K-HK-K-K-E-A-ADH-H-AIK-A--A------QQ------N--I-I-NQKE--D-IK-Q-K---I--HKQKR--G--C----IIG---C----R--G---C-R-C------------I-I------D----I--I------H---KH-I---Q---KH-Q--I-IHQ-KA-IDDA-----

>L11

--D-DD-D-D-LCR-GCGCLG--G----------L-L-L------R--D---I-------D----------------I------E----------K-N--N--H-----Q--K-N-----H-Q----A-HQKHHIIE-DI-I---IGG---CIDIGR---C-----CGLR------R-----D-I--A---E--I-K---K-----N-K---H-H-KQIDIHQKI-AAA-I--I--D-----L-R----C--A----ADA-D--EI-ADHKH-DA-----I--RGRLI-I---G-GR-A-AR-G-L-DI-L-G----------G--D----D-I----G--L----C-IIE--A-RG--R---CGGCE-I-CG-RC--R-I-I-CLC--RIDIAICLCIGG----LA------GD-I----II--A-II---E--I-------K-------N--K-K-I-HNHII-D-I-II-K--HNHI--I--K-H--KK-EH--I--I--A-H--Q---K-HK-K-K-E-A-ADH-H-AIK-A--A------QQ------N--I-I-NQKE--D-IK-Q-K---I--HKQKR--G--C----IIG---C----R--G---C-R-C------------I-I------D----I--I------H---KH-I---Q---KH-Q--I-IHQ-KA-IDD------

>L13

--D-DD-D-D-LCR-GCGCLG--G----------L-L-L------R--D---I-------D----------------I------E----------K-N--N--H-----Q--K-N-----H-Q----A-HQKHHIIE-DI-I---IGG---CIDIGR---C-----CGLR------R-----D-I--A---E--I-K---K-----N-K---H-H-KQIDIHQKI-AAA-I--I--D-----L-R----C--A----ADA-D--EI-ADHKH-DA-----I--RGRLI-I---G-GR-A-AR-G-L-DI-L-G----------G--D----D-I----G--L----C-IIE--A-RG--R---CGGCE-I-CG-RC--R-I-I-CLC--RIDIAICLCIGG----LA------GD-I----II--A-II---E--I-------K-------N--K-K-I-HNHII-D-I-II-K--HNHI--I--K-H--KK-EH--I--I--A-H--Q---K-HK-K-K-E-A-ADH-H-AIK-A--A------QQ------N--I-I-NQKE--D-IK-Q-K---I--HKQKR--G--C----IIG---C----R--G---C-R-C------------I-I------D----I--I------H---KH-I---Q---KH-Q--I-IHQ-KA-IDDA-----

>L116

--D-DD-D-D-LCR-GCGCLG--G----------L-L-L------R--D---I-------D----------------I------E----------K-N--N--H-----Q--K-N-----H-Q----A-HQKHHIIE-DI-I---IGG---CIDIGR---C-----CGLR------R-----D-I--A---E--I-K---K-----N-K---H-H-KQIDIHQKI-AAA-I--I--D-----L-R----C--A----ADA-D--EI-ADHKH-DA-----I--RGRLI-I---G-GR-A-AR-G-L-DI-L-G----------G--D----D-I----G--L----C-IIE--A-RG--R---CGGCE-I-CG-RC--R-I-I-CLC--RIDIAICLCIGG----LA------GD-I----II--A-II---E--I-------K-------N--K-K-I-HNHII-D-I-II-K--HNHI--I--K-H--KK-EH--I--I--A-H--Q---K-HK-K-K-E-A-ADH-H-AIK-A--A------QQ------N--I-I-NQKE--D-IK-Q-K---I--HKQKR--G--C----IIG---C----R--G---C-R-C------------I-I------D----I--I------H---KH-I---Q---KH-Q--I-IHQ-KA-IDDA-----

>AL56

-AI-DDDA-D-LCR-GCGLLG--G---L----L-L-R--------C-DD---I-------E----------------I-----------H-----K-H--N--N-----Q--Q-H-----H-Q----A-HQKHHIIA-II-D---IGG---CIIIGR---R----GG--GL----GCR----I-IIAI---D--IKH---------KHKQ--K-K-KQIIIHQKI-AAA-I--L--C-----L-R----D--A--L-RCA-I--EI-ADHKH-AA-----K--NHNID-I-GGR-G----IA-E-I-DL-L-G-I--------G--L------LI---G--L-DD-I--DA----GG--R---CGGCA-I-CG-GC--R-R----LCIDALCIAICLCLGG----LA------G--A-E--D-----EIDK-A--E-DE----N-------Q--K-H---HNHII-I-HNAA-D--HNEE--IK-K-H--QK--H--I--I--A-H--Q---K-HK-K-K---EEA-E-IHK-D-N--N------QQ------N--N-A-IDII--D-KK-Q-K-A-EI-EAAEG--G--C---LR-G---L----R--G--------------------D-I------I-------I------I----E-D---Q---KH-Q--K-NHK-QA-ADDAA----

>Mex20

--D-DD-A-D-LCR-GCGLCG--GLLLICCIIG-L-R-C------LREV---I-------ADKNHKNQ---------I------EIH--H-----I-H--N--H-----Q--K-H-----H-Q----A-HQKHHIIA-DI-I---IGG---CIIIGR---R----GR--R------CG----I-I--I------K-H---------K-K---K-K-KQIIDHQKI-AAA-I--L--C-----L-R----D--A--L-RCA-I--EI-ADHKH-AA-----K--NHNID-I-GGR-G----IA-E-I-DL-L-G-I--------G--L------LI---G--L-DD-I--II----GG--R---CGGCA-I-CR-GC--R-R----LCIDALCIAICLCLGG----LA------E--A-E--D-----EIDI-A--E-DE----N-------Q--K-H---HNHII-I-H-ND-D--HNEI--IK-K-H--KK--H--I--I--A-H--Q---K-HI-K-K-K-EEA-E-IHK-D-N--H------QQ------H--N-I-IDII--D-KK-Q-K-A-EI-EAAEG--G--C---LR-G---L----R--G---L-RGL--------R---D-I------I----E--I------K----H-QKNIQ---KH-K--K-NHK-QA-ADDA-----

>PnSL03

--I-ID-I-I-----E--LCI--G---I--IAC-L-R-G------L-AD---I-------A----------------I------E----E-----I-E--A--H-----Q--K-H-----H-D----A-EQIHHIIA-DI-I---IGG---CIIIGR---R----GR--R------CE----I-I--I------I-H---------K-K---K-K-KQIIDHQKI-AAA-I--L--C-----L-R----D--A--L-RCA-I--EI-ADHKH-AA-----K--NHNID-L-GGR-G----IR-G-I-DL-L-G-I--------G--L------LI---G--L-DC-L--LI----GG--R---CGGCA-I-CR-GC--R-R-I-CLC--AICIAICLCLGG----LA------E--A-E--D-----EIDI-A--E-DE----N-------Q--K-H---HNHII-I-H-AD-D--HNHI--IK-K-H--KK--H--I--I--A-H--Q---K-HK-K-K-I-EHN-H-IHK-D-N--H------QQ------H--N-I-IQKI--Q-KK-Q-I-N-EI-EAA-G--GE-C---LR-G---L----R--G---L-RGL--------R---D-I------I----E--I------K----H-QKNIQ---KH-K--K-NHK-QA-ADDA-----

>En345153B5

-ID-DD-A-D-LCR-GCRCLG--G----------L-G--------R------ILRC----I---ILRIIIIAIIIKNI------DHK--ND----K----Q--N-----Q--K-N-----H-K----A-HQKHHIIE-DI-I---IGG---CIIIGR---C----GG--L------C-----I-E-AA---D--I-H---------N-Q---K-H-KQIIIHQKI-AAA-I--L--C-----L-R----D--AIIC-RL-----EI-ADHKH-DA-----K--NHNID-L---R-G--A-ARCR----L-C-I-I--------GG-C----D-D----G--L----L--GR--R-IA--R---CGGLE-D-LG-GC--R---ILCLC--GLDLAICLCIGG----IR------G--C-L--I------I-A-E--H-HQ----K-------I--KQI-I-HNHII-I-ENIE-H-KHNHN--I--K-H--KK--N-----A--A-HQ-Q---I-HK-D-I-K-K-K-H---H-QAA--H------QQ---------I-I-HNKI--H-KEEQ-K-N-A--AIDIR--GC-L----RIG--CL-------G---L---L-R------RI--E-I------IAAAKK--N-----------N-D---Q---HH-Q--K-NHQ-KA-IDDAA----

>MC982

--D-DD-A-D-LCR-GCGCLR--G----------L-C-L------G--C---I-------D----------------I------H----------K-N--H--N-----Q--K-N-----H-K----A-HQKHHIIA-DI-A---IGG---CIIIGR---C-I--GC--L------------I-D---------I-H---------H-K---I-H-KQIIIHQKI-AAA-I--I--D-----L-R----C--A----ADA-I--EI-ADHKH-DA-----I--RGRLD-L---R-E--AIIG-G-I-AL-C-R----------G--D----D-I----G--LRA--L--II--I-I---R---CGGLE-D-LL-AC--R-A-I-CLC--DIDIAICLCLGG----IA------G--C----I------I---H--K--D----I-------Q--K-H---HNHII-I-D-AI-I--HHHE--I--K-H--AN--N-EI--D--A-N--Q---K-HK-I-I-E-E-N-A-K-N-Q-I--A------QK------H--N-D-IAKK--A-II-A-K-N-E--NKQKR--G--C----III---C----R--G----------------------I------I----I--A-------------I---Q---KH-I--I-AHQ-KA-IDDA-----

>MC985

--D-DD-A-D-LCR-GCGCLR--G----------L-C-L------G--C---I-------D----------------I------H----------K-N--H--N-----Q--K-N-----H-K----A-HQKHHIIA-DI-A---IGG---CIIIGR---C-I--GC--L------------I-D---------I-H---------H-K---I-H-KQIIIHQKI-AAA-I--I--D-----L-R----C--A----ADA-I--EI-ADHKH-DA-----I--RGRLD-L---R-E--AIIG-G-I-AL-C-R----------G--D----D-I----G--LRA--L--II--I-I---R---CGGLE-D-LL-AC--R-A-I-CLC--DIDIAICLCLGG----IA------G--C----I------I---H--K--D----I-------Q--K-H---HNHII-I-D-AI-I--HHHE--I--K-H--AN--N-EI--D--A-N--Q---K-HK-I-I-E-E-N-A-K-N-Q-I--A------QK------H--N-D-IAKK--A-II-A-K-N-E--NKQKR--G--C----III---C----R--G----------------------I------I----I--A-------------I---Q---KH-I--I-AHQ-KA-IDDA-----

>MC3007

--D-DD-A-D-LCR-GCGCLR--G----------L-C-L------G--C---I-------D----------------I------H----------K-N--H--N-----Q--K-N-----H-K----A-HQKHHIIA-DI-A---IGG---CIIIGR---C-I--GC--L------------I-D---------I-H---------H-K---I-H-KQIIIHQKI-AAA-I--I--D-----L-R----C--A----ADA-I--EI-ADHKH-DA-----I--RGRLD-L---R-E--AIIG-G-I-AL-C-R----------G--D----D-I----G--LRA--L--II--I-I---R---CGGLE-D-LL-AC--R-A-I-CLC--DIDIAICLCLGG----IA------G--C----I------I---H--K--D----I-------Q--K-H---HNHII-I-D-AI-I--HHHE--I--K-H--AN--N-EI--D--A-N--Q---K-HK-I-I-E-E-N-A-K-N-Q-I--A------QK------H--N-D-IAKK--A-II-V-K-N-E--NKQKR--G--C----III---C----R--G----------------------I------I----I--A-------------I---Q---KH-I--I-AHQ-KA-IDDA-----

>MC3011

--D-DD-A-D-LCG-GCGCLR--G---IR--LLGL----------C------I--IEEDAA----------------D------I-I--H-----N-K--H--N-----K--QKH-----H-K----A-HQQHHIIA-DI-I---IGG---CIIIGR---L----EG--L-----GCRD---I-I--E------K-H----K----H-Q---I-H-KQIIDHQKE-EAA-I--L--C-----L-G----D--A--G--LI-I--ED-AIIIH-QA-----K--NHNIDDI-G-R-G-----RAE-I-DL-C-L----------G--R------LD---G--L-DCCI--E---I-G---R---CGGCC-I-CG-GC--R-R-I-CLC--ALCIAICLCLGG----IAE----DR--G-G--E------I-A-A----QQ----KD-EA--IQ--K-H---HNHII-I-H-NA----HNHD--IK-K-H--KK--H--I--I--H-H--K---K-HK-K-I-A-I-H-H-I-H-Q-AI-H----K-KH------H--N-I-EIDA--K-QK-Q-D-I-E--EIDLR--GCII----G-----C----R--G-------G--------G---DDI------V----E--E------D----K-N---Q---KH-Q--D-IHQ-KNIDD-A-----

>MC3012

--D-DD-A-D-LCG-GCGCLR--G---IR--LLGL----------C------I--IEEDAA----------------D------I-I--H-----N-K--H--N-----K--QKH-----H-K----A-HQQHHIIA-DI-I---IGG---CIIIGR---L----EG--L-----GCRD---I-I--E------K-H----Q----H-Q---I-H-KQIIDHQKE-EAA-I--L--C-----L-G----D--A--G--LI-I--ED-AIIIH-QA-----K--NHNIDDI-G-R-G-----RAE-I-DL-C-L----------G--R------LD---G--L-DCCL--E---I-G---R---CGGCC-I-CG-GC--R-R-I-CLC--ALCIAICLCLGG----IAE----DR--G-G--E------I-A-A----QQ----KD-EA--IQ--K-H---HNHII-I-H-NA----HNHD--IK-K-H--KK--H--I--I--H-H--K---K-HK-K-I-A-N-H-H-I-H-Q-AI-H----K-KH------H--N-I-EIDA--K-QK-Q-D-I-E--EIDLR--GCII----G-----C----R--G-------G--------G---DDI------V----E--E------D----K-N---Q---KH-Q--D-IHQ-KNIDD-A-----

>PnSL05

--D-II-E-I-----AC-LR---G---I--IAC-L-R-G------L-AD---I-------A----------------I------E----E-----I-E--A--H-----Q--K-H-----H-D----A-EQKHHIIA-DI-I---IGG---CIIIGR---R----GR--R------CE----I-I--I------I-H---------K-K---K-K-KQIIDHQKI-AAA-I--L--C-----L-R----D--A--L-RCA-I--EI-ADHKH-AA-----K--NHNID-L-GGR-G----IR-G-I-DL-L-G-I--------G--L------LI---G--L-DC-L--LI----GG--R---CGGCA-I-CR-GC--R-R-I-CLC--AICIAICLCLGG----LA------E--A-E--D-----EIDI-A--E-DE----N-------Q--K-H---HNHII-I-H-AD-D--HNHI--IK-K-H--KK--H--I--I--A-H--Q---K-HK-K-K-I-EHN-H-IHK-D-N--H------QQ------H--N-I-IQKI--Q-KK-Q-I-N-EI-EAA-G--GE-C---LR-G---L----R--G---L-RGL--------R---D-I------I----E--I------K----H-QKNIQ---KH-K--K-NHK-QA-ADDA-----

>DS2

--D-DD-A-D-LCR-GCGCLG--G----------L-G-I------L--R---I-------I--------E-------I------K----N-----I----Q--N-----K--Q-H-----H-I----K-HQKHHIIE-DI-I---IGG---CIIIGRCGGL----GG--LI-GCLGCL----D-D-AE------INHDQNHQDHQKH-Q---K-H-KQIIIHQKI-AAA-I--L--C-----L-R----D--AI-C-RC--I--EI-ADHKH-DA-----K--NHNID-L---R-GR-A-AL-G-IDAL-GGI-I----------------D-L----G--L-D--L--GA--A-IG--R---CGGCE-I-LG-RC--R-R-I-CLC--RIDIAICLCLGG----IR------G--D----I------I---E--I--Q----K-------Q--K-H---HNHII-I-A-AA-I-KHNHI--K--K-H--KK--H--I--I--A-H--Q---K-HK-K-I-E-D-Q-N-A-N-QHI--I----D-QQ------H--E-I-QNEI--E-K--Q-K-NDE--AIDLG--GG-L----R-G---L-------G-------G--------------IIGLGGIE---IDIID------I----KQN---QIIQQN-Q--K-NKQ-KN-DADA-----

>AL112

--D-DD-A-D-LCR-GCGCLG--G----------L-G-I------L--R---I-------I--------E-------I------K----N-----I----Q--N-----K--Q-H-----H-I----K-HQKHHIIE-DI-I---IGG---CIIIGRCGGL----GG--LI-GCLGCL----D-D-AE------INHDQNHQDHQKH-Q---K-H-KQIIIHQKI-AAA-I--L--C-----L-R----D--AI-C-RC--I--EI-ADHKH-DA-----K--NHNID-L---R-GR-A-AL-G-IDAL-GGI-I----------------D-L----G--L-D--L--GA--A-IG--R---CGGCE-I-LG-RC--R-R-I-CLC--RIDIAICLCLGG----IR------G--D----I------I---E--I--Q----K-------Q--K-H---HNHII-I-A-AA-I-KHNHI--K--K-H--KK--H--I--I--A-H--Q---K-HK-K-I-E-D-Q-N-A-N-QHI--I----D-QQ------H--E-I-QNEI--E-K--Q-K-NDE--AIDLG--GG-L----R-G---L-------G-------G--------------IIGLGGIE---IDIID------I----KQN---QIIQQN-Q--K-NKQ-KN-DADA-----

>PnSb58

--I-DD-A-A-LLRGEG-LLG--G---L----L-L-G-C------CCEI---I-------ADI--------------I------EI---AD----I-E--H--HHQHHHQ--K-H-----H-Q----A-EQKHHIIA-DI-I---IGG---CIIIGR---G----GG--R------CR----I-I-EI---D--IKH---------K-Q---Q-K-KQIIIHQKI-EAA-I--L--C-----L-R----D--A--L-RCA-I--EI-ADHKH-AA-----K--NHNID-L-GRR-G----IRIG-I-DL-L-G-I--------G--L------LL---G--L-DC-L--LL----GG--R---CGGCA-I-CR-GC--R-R-L-CLC--ALCIAICLCLGG----LA------E--A-E--D-----EIDI-A--E-DE----N-------Q--K-H---HNHII-I-H-ND-D--HNHH--IK-K-H--KK--H--I--I--A-H--Q---K-HK-E-K-K-HHN-H-IHK-H-N--N------QQ------H--N-I-IQIK--D-KK-K-Q-N-EI-EAA-G--G--GD--LR-G---L----R--GIAGL-RDL--------R---A-A-----AD----E--E------K----H-DKNKQ---KH-K--K-HHK-QA-ADDA-----

>AL17

AID-DD-A-D-LCR-GCRCLG--G----------L-G--------RL-----I-AD----I----------------I------I-I--N-----K----K--H-----Q--K-N-----H-K----A-HQKHHIIA-DI-I---IGG---CIIIGR---C----GG--L------C-----I-E-AA---D--I-H---------N-Q---K-H-KQIIIHQKI-AAA-I--L--C-----L-R----D--AIIC-RL-----EI-ADHKH-DA-----K--NHNID-L---R-G--A-RRCG----L-C-I-I--------GG-C----D-D----G--L----L--GR--R-IA--R---CGGLE-D-LG-GC--R---ILCLC--RLDLAICLCIGG----IR------G--C-L--I------I-A-E--H-HQ----K-------I--KQI-I-HNHII-I-ANIA-H-KHNHN--I--K-H--KK--N-----A--A-HQ-Q---I-HK-D-I-K-K-K-H---H-QAA--H------QQ---------I-D-HNQI--H-KKEQ-K-N-I--AIDIR--GC-L----RIG--CL-------G---C---L--------RR--I-I------I--AKK--N-----------H-I---Q---HH-Q--K-NHQ-KA-IDDAA----

>NWFSC189

-ID-DD-A-D-LCR-GCRCLG--G----------L-G--------R------I-------I----------------I------D----------K----Q--N-----Q--K-N-----H-K----A-HQKHHIIA-DI-I---IGG---CIIIGR---C----GG--L------C-----I-E-AA---D--I-H---------N-Q---K-H-KQIIIHQKI-AAA-I--L--C-----L-R----D--AIIC-RL-----EI-ADHKH-DA-----K--NHNID-L---R-G--A-RRCG----L-C-I-I--------GG-C----D-D----G--L----L--GR--R-IA--R---CGGLE-D-LG-RC--R---ILCLC--GLDLAICLCIGG----IR------G--C-L--I------I-A-D--H-HQ----K-------I--KQI-I-HNHII-I-ANIE-H-KHNHN--I--K-H--KK--N-----A--A-HQ-Q---I-HK-D-I-K-K-K-H---H-QAA--H------QQ---------I-I-HNKI--H-KKEQ-K-N-A--EIDIR--GC-L----RIG--CL-------G---L---L--------R---A-I------I----K--N-----------N-I---Q---HH-Q--K-NHQ-KA-IDDAA----

>PnSg10

-ID-DD-A-D-LCR-GCRCLG--G----------L-G--------R------I--I----I----------------I------A-I--A-----K----K--H-----Q--K-N-----H-K----A-HQKHHIIA-DI-I---IGG---CIIIGR---C----GG--L------C-----I-E-AA---D--I-H---------N-Q---K-H-KQIIIHQKI-AAA-I--L--C-----L-R----D--AIIC-RL-----EI-ADHKH-DA-----K--NHNID-L---R-G--A-RRCG----L-C-I-I--------GG-C----D-D----G--L----L--GR--R-IA--R---CGGLE-D-LG-GC--R---ILCLC--GLDLAICLCIGG----IR------G--C-L--I------I-A-E--H-HQ----K-------I--KQI-I-HNHII-I-ANIE-H-KHNHN--I--K-H--KK--N-----A--A-HQ-Q---I-HK-D-I-K-K-K-H---H-QAA--H------QQ---------I-I-HNKI--H-KKEQ-K-N-A--AIDIR--GC-L----RIG--CL----E--G---C---L--------R---A-I------D----K--N-----------H-I---Q---HH-Q--K-NHQ-KA-IDDAA----

>Sydney1

-ID-DD-A-D-LCR-GCRCLG--G----------L-G--------RL-----I-AD----I----------------I------I-I--N-----K----K--H-----Q--K-N-----H-K----A-HQKHHIIA-DI-I---IGG---CIIIGR---C----GG--L------C-----I-E-AA---D--I-H---------N-Q---K-H-KQIIIHQKI-AAA-I--L--C-----L-R----D--AIIC-RL-----EI-ADHKH-DA-----K--NHNID-L---R-G--A-RRCG----L-C-I-I--------GG-C----D-D----G--L----L--GR--R-IA--R---CGGLE-D-LG-GC--R---ILCLC--RLDLAICLCIGG----IR------G--C-L--I------I-A-E--H-HQ----K-------I--KQI-I-HNHII-I-ANIA-H-KHNHN--I--K-H--KK--N-----A--A-HQ-Q---I-HK-D-I-K-K-K-H---H-QAA--H------QQ---------I-D-HNQI--H-KKEQ-K-N-I--AIDIR--GC-L----RDG--CL-------G---C---L--------RR--I-I------I--AKK--N-----------H-I---Q---HH-Q--K-NHQ-KA-IDDAA----

>Tenerife8

-ID-DD-A-D-LCR-GCRCLG--G----------L-G--------R------I-------I----------------I------D----------K----Q--N-----Q--K-N-----H-K----A-HQKHHIIA-DI-I---IGG---CIIIGR---C----GG--L------C-----I-E-AA---D--I-H---------N-Q---K-H-KQIIIHQKI-AAA-I--L--C-----L-R----D--AIIC-RL-----EI-ADHKH-DA-----K--NHNID-L---R-G--A-RRCG----L-C-I-I--------GG-C----D-D----G--L----L--GR--R-IA--R---CGGLE-D-LG-RC--R---ILCLC--GLDLAICLCIGG----IR------G--C-L--I------I-A-D--H-HQ----K-------I--KQI-I-HNHII-I-ANIE-H-KHNHN--I--K-H--KK--N-----A--A-HQ-Q---I-HK-D-I-K-K-K-H---H-QAA--H------QQ---------I-I-HNKI--H-KKEQ-K-N-A--EIDIR--GC-L----RIG--CL-------G---L---L--------R---A-I------I----K--N-----------N-I---Q---HH-Q--K-NHQ-KA-IDDAA----

>Mex12

-ID-DD-A-D-LCR-GCRCLG--G----------L-G--------R------I--I----I----------------I------A-I--A-----K----K--H-----Q--K-N-----H-K----A-HQKHHIIA-DI-I---IGG---CIIIGR---C----GG--L------C-----I-E-AA---D--I-H---------N-Q---K-H-KQIIIHQKI-AAA-I--L--C-----L-R----D--AIIC-RL-----EI-ADHKH-DA-----K--NHNID-L---R-G--A-ARCG----L-C-I-I--------GG-C----D-D----G--L----L--GR--R-IA--R---CGGLE-D-LG-GC--R---ILCLC--GLDLAICLCIGG----IR------G--C-L--I------I-A-E--H-HQ----K-------I--KQI-I-HNHII-I-ANIE-H-KHNHN--I--K-H--KK--N-----A--A-HQ-Q---I-HK-D-I-K-K-K-H---H-QAA--H------QQ---------I-I-HNKI--H-KAEQ-K-N-A---IDIR--GC-L----RIG--CL----E--G---C---L--------R---A-I------D----K--N-----------H-I---Q---NH-Q--K-NHQ-KA-IDDAA----

>GranCan41

--D-DD-D-D-LCR-GCRCLR--G---------------------R--E---I-------A----------------I-----------------K-------------Q--K-N-----H-K----A-HQKHHIIE-DI-I---IGG---CIIIGR---C-----CG-L------D-----D-I--A---E--I-E---------H-K---H-H-KQIDIHQKI-AAA-I--I--D-----L-R----C--A--LEA---D--EI-NDHKH-DA-----I--RGRLD-L---R-GR-A-AR-G-L-DL-L-G----------G--D----D-I----G--L----C-IEA--A-RG--R---CGGCE-I-LG-RL--R-I-I-CLC--RIDIAICLCLGG----IR------G--D----I------I---E--I--Q----K-------Q--K-H---HNHII-D-A--D-K--HNHI--I--K-H--KQ--N--D--I--A-H--Q---K-HK-K-K-E-A-AIH---N-Q-I--A------QK------N--N-A-NQKE--I-DK-Q-K-N-A--HKQKR--G--C----IIG---C----R--G--------------------D-I------I----E--I-----------------Q---KH-Q--I-DHQ-KA-IDDA-----

>Mex13

--D-DD-D-D-LCR-GCRCLR--G---------------------R--E---I-------A----------------I-----------------K-------------Q--K-N-----H-K----A-HQKHHIIE-DI-I---IGG---CIIIGR---C-----CG-L------D-----D-I--A---E--I-E---------H-K---H-H-KQIDIHQKI-AAA-I--I--D-----L-R----C--A--LEA---D--EI-NDHKH-DA-----I--RGRLD-L---R-GR-A-AR-G-L-DL-L-G----------G--D----D-I----G--L----C-IEA--A-RG--R---CGGCE-I-LG-RC--R-I-I-CLC--RIDIAICLCLGG----IR------G--D----I------I---E--I--Q----K-------Q--K-H---HNHII-D-A--D-K--HNHA--I--K-H--KK--N--D--I--A-H--Q---K-HK-K-K-E-A-AIH---N-Q-I--A------QK------N--N-A-NQKE--I-DK-Q-K-N-A--HKQKR--G--C----IIG---C----R--G--------------------D-I------I----E--I-----------------Q---KH-Q--I-DHQ-KA-IDDA-----

>PnKk38

--D-DD-D-D-LCR-GCRCLR--G---------------------R--E---I-------A----------------I-----------------K-------------Q--K-N-----H-K----A-HQKHHIIE-DI-I---IGG---CIIIGR---C-----CG-L------D-----D-I--A---E--I-E---------H-K---H-H-KQIDIHQKI-AAA-I--I--D-----L-R----C--A--LEA---D--EI-NDHKH-DA-----I--RGRLD-L---R-GR-A-AR-G-L-DL-L-G----------G--D----D-I----G--L----C-IEA--A-RG--R---CGGCE-I-LG-RC--R-I-I-CLC--RIDIAICLCLGG----IR------G--D----I------I---E--I--Q----K-------Q--K-H---HNHII-D-A--D-K--HNHA--I--K-H--KK--N--D--I--A-H--Q---K-HK-K-K-E-A-AIH---N-Q-I--A------QK------N--N-A-NQKE--I-DK-Q-K-N-A--HKQKR--G--C----IIG---C----R--G--------------------D-I------I----E--I-----------------Q---KH-Q--I-DHQ-KA-IDDA-----

>Laeso2

--D-DD-A-D-LCR-GCRCLG--G----------L-C-L------R--L---I-------D----------------I------H----------K-N--H--H-----Q--K-N-----H-K----D-HQKHHIIE-DI-I---IGG---CDIIGR---C-I--GC--C------I-----D-I-----------H---------H-Q---I-H-KQIDIHQKI-AAA-I--I--D-----L-R----C--A--ADA---D--EI-ADHKH-DA-----I--RGRLD-L---R-GA-A-AC-G-I-CL-C-R----------G--D----D-D----G--L----L-CLA--A-DI--R---CGGCE-I-LG-RC--R-I-I-CLC--RIDIAICLCLGG----IA------G--C----I------I-IIH--K--D----I-------Q--K-H---HNHII-D-A-AI-K--HNHI--I--K-H--KK--N--I--D--A-H--Q---K-HK-I-E-E-A-NHH---N-Q-I--A------QK------H--N-H-IDKH--A-DA-Q-K-N-A--HKQKR--G--C----IIG---C----R--G--------------------I-I------I-------I-----------------Q---KH-Q--I-DHQ-KA-IDIA-----

>OFPd972

--D-DD-A-D-LCR-GCRCLG--G----------I-C-L------R--L---I-------D----------------IIIADEAH----------K-N--H--I-----Q--K-N-----H-K----A-HQKHHIIE-DI-I---IGG---CDIIGR---C-I--GC--C------IAIIAID-I-----------H---------H-Q---I-H-KQIDIHQKI-AAA-I--I--D-----L-R----C--A--ADA---D--EI-ADHKH-DA-----I--RGRLD-L---R-GR-A-ED-G-L-CI-C-R----------G--D----D-D----G--L----I-CLA--A-CG--R---DGGCE-I-LG-RC--R-I-I-CLC--RIDIAICLCLGG----IA------G--C----I------I---H--K--D----I-------Q--K-H---HNHII-D-A-AI-K--HNHI--I--K-H--KK--N--I--D--A-H--Q---K-AK-Q-H-E-A-NHI---N-Q-I--A------QK------H--A-E-IDHN--K-DK-Q-K-N-A--HKQKR--G--C----IIG---C----R--G--------------------I-I------I-------A-----------------Q---KH-Q--I-DHQ-KA-IDIA-----

>NWFSC090

--D-DD-A-D-LCR-GCRCLG--G----------L-C-L------R--L---I-------D----------------I------H----------K-N--H--H-----Q--K-N-----H-K----D-HQKHHIIE-DI-I---IGG---CDIIGR---C-I--GC--C------I-----D-I-----------H---------H-Q---I-H-KQIDIHQKI-AAA-I--I--D-----I-A----D--A--ADA---D--EI-AIE-E-DA-----I--RGRLD-L---R-GA-A-AC-G-I-CL-C-R----------G--D----D-D----G--L----L-CLA--A-DI--R---CGGCE-I-LG-RC--R-I-I-CLC--RIDIAICLCLGG----IA------G--C----I------I-IIH--K--D----I-------Q--K-H---HNHII-D-A-AI-K--HNHI--I--K-H--KK--N--I--D--A-H--Q---K-HK-I-E-E-A-NHH---N-Q-I--A------QK------H--N-H-IDKH--A-DA-Q-K-N-A--HKQKR--G--C----IIG---C----R--G--------------------I-I------I-------I-----------------Q---KH-Q--I-DHQ-KA-IDIA-----

>AL59

--D-DD-D-D-LCR-GCGCLG--G----------L-L-L------R--D---I-------D----------------I------E----------K-N--N--H-----Q--K-H-----H-Q----A-HQKHHIIE-DI-I---IGG---CIDIGR---C-----CG-L------A-----D-I--A---E--D-E---------N-Q---H-H-KQIDIHQKI-AAA-I--L--C-----L-R----D--A----ACR-C--EI-ADHKH-DA-----K--NHNII-I---G-GA-A-AR-G-L-DI-L-G----------G--D----D-I----G--L----C-LLE--A-AG--R---CGGCE-L-CG-RC--R-I-I-CLC--RIDIAICLCIGG----LR------G--D----I------I---E--I--Q----K-------N--K-K-IIHNHII-D-I-II-K--HNHI--I--K-H--KK-HH--I--I--A-H--Q---K-HK-K-D-E-A-NHH---N-KIA--A------QQ------N--I-A-NQKE--Q-KI-D-I---I--GLDIA--G--C----ILG---D----R--G--------------------D-E------D----IA-IAQKAKNHKEIAH-I-EIQ---IE-D--I-IED-IA-IDDA-----

>Calif1

--D-DD-D-D-LCR-GCGCLG--G----------L-L-L------R--D---I-------D----------------I------E----------K-N--N--H-----Q--K-H-----H-Q----A-HQKHHIIE-DI-I---IGG---CIDIGR---C-----CG-L------A-----D-I--A---E--D-E---------N-Q---H-H-KQIDIHQKI-AAA-I--L--C-----L-R----D--A----ACR-C--EI-ADHKH-DA-----K--NHNII-I---G-GA-A-AR-G-L-DI-L-G----------G--D----D-I----G--L----C-LLE--A-AG--R---CGGCE-L-CG-RC--R-I-I-CLC--RIDIAICLCIGG----LR------G--D----I------I---E--I--Q----K-------N--K-K-IIHNHII-D-I-II-K--HNHI--I--K-H--KK-HH--I--I--A-H--Q---K-HK-K-D-E-A-NHH---N-KIA--A------QQ------N--I-A-NQKE--Q-KI-D-I---I--GLDIA--G--C----ILG---D----R--G--------------------D-E------D----IA-IAQKAKNHKEIAH-I-EIQ---IE-D--I-IED-IA-IDDA-----

>AR3

--D-DD-A-D-LCR-GCGCLG--G----------L-C-L------R--C---I-------D----------------I------H----------K-N--HH-D-----Q--K-N-----H-I----K-HQKHHIIE-DI-A---IGG---CIDIGR---C-C--GC--L------------D-I--A--------E---IN----H-Q---H-H-KQIDIHQKI-AAA-I--L--C-----L-R----D--A----ACRLI--EI-ADHKH-DA-----K--NHNID-L---R-GR-R-RR-G-I-CL-L-G----------I--E----I-I----G--L-D--L--GA--A-IG--R---CGGCE-I-IG-RC--G-C-L-CLL--GLDIAICLCLGG----IR------G--D----ILGLRLEA---AANK-HQN---I------IQKQK-H---HNHII-I-A-NQ-I--NHHH--H--K-H--KQ-IA--I--I--A-H--Q---K-HK-Q-I-E-D-QNA---N-Q-I--A------IQ------H--N-H-KKKK--I-DK-Q-K-H-E--EIDLG--G--CC---RDG---C----R--G--CL-G-L---------E--I-A------A----A--AHQ----N----H-I---Q---KH-Q--K-HHQ-KN-IDDA-----

>F10

--D-DD-A-D-LCR-GCGCLG--G----------L-C-L------R--C---I-------D----------------I------H----------K-N--HH-D-----Q--Q-N-----H-I----K-HQKHHIIE-DI-A---IGG---CIDIGR---C-C--GC--L------------D-I--A--------E---IN----H-Q---H-H-KQIDIHQKI-AAA-I--L--C-----L-R----D--A----ACRLI--EI-ADHKH-DA-----K--NHNID-L---R-GR-R-RR-G-I-CL-L-G----------I--E----I-I----G--L-D--L--GA--A-IG--R---CGGCE-I-IG-RC--G-C-L-CLL--GLDIAICLCLGG----IR------G--D----ILGLRLEA---AANK-HQN---I------IQKQK-H---HNHII-I-A-NQ-I--NHHH--H--K-H--KQ-IA--I--I--A-H--Q---K-HK-Q-I-E-D-QNA---N-Q-I--A------IQ------H--N-H-KKKK--I-DK-Q-K-H-E--EIDLG--G--CC---RDG---C----R--G--CL-G-L---------E--I-A------A----A--AHQ----N----H-I---Q---KH-Q--K-HHQ-KN-IDDA-----

>Limens1

--D-DD-A-D-LCR-GCGCLG--G----------L-C-L------R--C---I-------D----------------I------H----------K-N--HH-D-----Q--K-N-----H-I----K-HQKHHIIE-DI-A---IGG---CIDIGR---C-C--GC--L------------D-I--A--------E---IN----H-Q---H-H-KQIDIHQKI-AAA-I--L--C-----L-R----D--A----ACRLI--EI-ADHKH-DA-----K--NHNID-L---R-GR-R-RR-G-I-CL-L-G----------I--E----I-I----G--L-D--L--GA--A-IG--R---CGGCE-I-IG-RC--G-C-L-CLL--GLDIAICLCLGG----IR------G--D----ILGLRLEA---AANK-HQN---I------IQKQK-H---HNHII-I-A-NQ-I--NHHH--H--K-H--KQ-IA--I--I--A-H--Q---K-HK-Q-I-E-D-QNA---N-Q-I--A------IQ------H--N-H-KKKK--I-DK-Q-K-H-E--EIDLG--G--CC---RDG---C----R--G--CL-G-L---------E--I-A------A----A--AHQ----N----H-I---Q---KH-Q--K-HHQ-KN-IDDA-----

>NWFSC241

--D-DD-A-D-LCR-GCRCLG--G----------L-C-L------R--L---I-------D----------------I------H----------K-N--H--N-----Q--Q-H-----H-I----K-HQKHHIIA-AI-D---IGG---CDIIGR---C-I--GC--C------I-----I-I-----------H---------H-Q---I-H-KQIIIHQKI-AAA-I--L--L-----L-RD---L--I--L-R---L--EI-ADHKH-DN-----K--NHNID-L---R-GR-A-AR-G-I-CL-C-G----------E--D----D-I----G--L-D--L--GA--A-RG--R---CGGCE-I-LG-RC--R-R-I-CLC--RIDIAICLCLGG----IA------R--C----I------I-I-A--I--IH---KA-----IQ--K-H---HNHII-D-A-AD-K--HNHI--K--K-H--KK--N--I--D--A-H--Q---K-HK-K-K-E-A-QNI---N-Q-I--A------AQ------H--N-H-IQKE--I-DK-Q-K-N-A--EIVIE--G--C----IIG---C----R--G---L-GGLG-------RRC-A-I------I----AHKK-K----N----K-Q-NDQ---KH-Q--I-DHQ-IA-ID-A-----

>NWFSC242

--D-DD-A-D-LCR-GCRCLG--G----------L-C-L------R--L---I-------D----------------I------H----------K-N--H--N-----Q--Q-H-----H-I----K-HQKHHIIA-AI-D---IGG---CDIIGR---C-I--GC--C------I-----I-I-----------H---------H-Q---I-H-KQIIIHQKI-AAA-I--L--L-----L-RD---L--I--L-R---L--EI-ADHKH-DN-----K--NHNID-L---R-GR-A-AR-G-I-CL-C-G----------E--D----D-I----G--L-D--L--GA--A-RG--R---CGGCE-I-LG-RC--R-R-I-CLC--RIDIAICLCLGG----IA------R--C----I------I-I-A--I--IH---KA-----IQ--K-H---HNHII-D-A-AD-K--HNHI--K--K-H--KK--N--I--D--A-H--Q---K-HK-K-K-E-A-QNI---N-Q-I--A------AQ------H--N-H-IQKE--I-DK-Q-K-N-A--EIDIE--G--C----IIG---C----R--G---L-GGLG-------RRC-A-I------I----AHKK-K----N----K-Q-NDQ---KH-Q--I-DHQ-IA-ID-A-----

>PnKk36

-DD-DD-A-D-LCR-GCGCLG--G----------L-G--------R------D-------I----------------D------E----------K----Q--H-----Q--Q-H-----H-K----A-HQKHHIIE-DI-I---IGG---CIIIGR---C----GG--L------C-----I-E-AA---D--I-H---------H-Q---K-H-KQIIIHQKI-AAA-I--L--C-----L-R----D--AIIC-RL-----EI-ADHKH-DA-----K--NHNID-L---R-G--R-RRCG----L-C-L-I--------GG-C----D-D----G--L----L--GR--R-LA--G---CGGCE-D-LG-GC--R---LLCLC--GLCLRICLCLGG----IR------G--C-L--D------I-D-E--H-HQ----K-------I--KQH-I-HNHII-I-KNDH-H-KHNHN--N--K-H--QK--N-----A--A-HQ-Q---I-HQ-D-N-K-K-K-H---H-QAA--H------QQ---------I-H-HNKE--H-KKKQ-K-N-A--EIDIR--GC-L----RDG--CC-------G--EL---LCG------GC--D-IDEH---I---QKH-N-----------N-A---Q---HH-K--K-NHQ-KA-IDDAA----

>PnMi158

-DD-DD-A-D-LCR-GCGCLG--G----------L-G--------R------D-------I----------------D------E----------K----Q--H-----Q--Q-H-----H-K----A-HQKHHIIE-DI-I---IGG---CIIIGR---C----GG--L------C-----I-E-AA---D--I-H---------H-Q---K-H-KQIIIHQKI-AAA-I--L--C-----L-R----D--AIIC-RL-----EI-ADHKH-DA-----K--NHNID-L---R-G--R-RRCG----L-C-L-I--------GG-C----D-D----G--L----L--GR--R-LA--G---CGGCE-D-LG-GC--R---LLCLC--GLCLRICLCLGG----IR------G--C-L--D------I-D-E--H-HQ----K-------I--KQH-I-HNHII-I-KNDH-H-KHNHN--N--K-H--QK--N-----A--A-HQ-Q---I-HQ-D-N-K-K-K-H---H-QAA--H------QQ---------I-H-HNKE--H-KKKQ-K-N-A--EIDIR--GC-L----RDG--CC-------G--EL---LCG------GC--D-IDEH---I---QKH-N-----------N-A---Q---HH-K--K-NHQ-KA-IDDAA----

>PnTb25

-DD-DD-A-D-LCR-GCGCLG--G----------L-G--------R------D-------I----------------D------E----------K----Q--H-----Q--Q-H-----H-K----A-HQKHHIIE-DI-I---IGG---CIIIGR---C----GG--L------C-----I-E-AA---D--I-H---------H-Q---K-H-KQIIIHQKI-AAA-I--L--C-----L-R----D--AIIC-RL-----EI-ADHKH-DA-----K--NHNID-L---R-G--R-RRCG----L-C-L-I--------GG-C----D-D----G--L----L--GR--R-LA--G---CGGCE-D-LG-GC--R---LLCLC--GLCLRICLCLGG----IR------G--C-L--D------D-D-E--H-HQ----K-------I--KQH-I-HNHII-I-KNDH-H-KHNHN--N--K-H--QK--N-----A--A-HQ-Q---I-HQ-D-N-K-K-K-H---H-QAA--H------QQ---------I-H-HNKE--H-KKKQ-K-N-A--EIDIR--GC-L----RDG--CC-------G--EL---LCG------GC--D-IDEH---I---QKH-N-----------N-A---Q---HH-K--K-NHQ-KA-IDDAA----

>PnTb31

-DD-DD-A-D-LCR-GCGCLG--G----------L-G--------R------D-------I----------------D------E----------K----Q--H-----Q--Q-H-----H-K----A-HQKHHIIE-DI-I---IGG---CIIIGR---C----GG--L------C-----I-E-AA---D--I-H---------H-Q---Q-H-KQIIIHQKI-AAA-I--L--C-----L-R----D--AIIC-RL-----EI-ADHKH-DA-----K--NHNID-L---R-G--R-RRCG----L-C-L-I--------GG-C----D-D----G--L----L--GR--R-LA--G---CGGCE-D-LG-GC--R---LLCLC--GLCLRICLCLGG----IR------G--C-L--D------I-D-E--H-HQ----K-------I--KQH-I-HNHII-I-KNDH-H-KHNHN--N--K-H--QK--N-----A--A-HQ-Q---I-HQ-D-N-K-K-K-H---H-QAA--H------QQ---------I-H-HNKE--H-KKKQ-K-N-A--EIDIR--GC-L----RDG--CC-------G--EL---LCG------GC--D-IDEH---I---QKH-N-----------N-A---Q---HH-K--K-NHQ-KA-IDDAA----

>ICMB173

--D-DD-D-D-LCR-GCRCLR--G----------L-G-L------R--I---I-------D----------------------------------K-N--K--H-----Q--K-N-----H-K----I-HQKHHIIE-DI-I---IGG---CIIIGR---C-----C--I-----ID-----D-I--I---E--D-E---------E-D---H-H-KQIDIHQKI-AAA-I--I--D-----L-R----C--A--LEA---D--EI-NDHKH-DA-----I--RGRLD-L---R-GR-A-AR-G-I-DL-L-G----------G--D----I-I----G--L----C-DEA--A-GG--R---CGGCEDI--G-RC--R-L-L-CLC--RIDIAICLCLGG----IA------G--C----I------I---H--K--D----I-------Q--K-H---HNHII-D-A--D-K--HNHINND--K-H--KQ--I--I--I--A-H--Q---K-HK-K-Q-E-A-ADH---N-Q-I--A------QK------N--N-I-IQKE--I-DK-Q-K-N-A--HKQKR--G--C----DIG---C----R--G---C---C------------D-I------I----E--I-I----H----H-D---Q---KH-Q--I-DHQ-KA-IDDA-----

>104A3

--D-DD-D-D-LCR-GCRCLR--G----------L-G-L------R--I---I-------D----------------------------------K-N--K--H-----Q--K-N-----H-K----I-HQKHHIIE-DI-I---IGG---CIIIGR---C-----C--I-----ID-----D-I--I---E--D-E---------E-D---H-H-KQIDIHQKI-AAA-I--I--D-----L-R----C--A--LEA---D--EI-NDHKH-DA-----I--RGRLD-L---R-GR-A-AR-G-I-DL-L-G----------G--D----I-I----G--L----C-DEA--A-GG--R---CGGCEDI--G-RC--R-L-L-CLC--RIDIAICLCLGG----IA------G--C----I------I---H--K--D----I-------Q--K-H---HNHII-D-A--D-K--HNHINND--K-H--KQ--I--I--I--A-H--Q---K-HK-K-Q-E-A-ADH---N-Q-I--A------QK------N--N-I-IQKE--I-DK-Q-K-N-A--HKQKR--G--C----DIG---C----R--G---C---C------------D-I------I----E--I-I----H----H-D---Q---KH-Q--I-DHQ-KA-IDDA-----

>Sydney4

--D-DD-D-D-LCR-GCRCLG--G----------L-G-L------R--L---I-------D----------------I------H----------K-N--Q--H-----Q--Q-N-----H-K----A-HQKHHIIE-DI-I---IGG---CIIIGR---C-----CE-I-----DD-----D-I--I---E--I-E---------E-I---H-H-KQIDIHQKI-AAA-I--I--D-----L-R----C--A--LEA---D--EI-NDHKH-DA-----I--RGRLD-L---R-GR-A-AR-G-I-DL-L-G----------G--D----I-I----G--L----C-DEA--A-GG--R---CGGCADI--G-RC--R-I-L-CLC--RIDIAICLCLGG----IA------G--C----I------I---H--K--D----I-------Q--K-H---HNHII-D-A--D-K--HNHINII--K-H--KQ--D--I--I--A-H--Q---K-HK-K-K-E-A-ADH---N-Q-I--A------QK------N--N-I-IQKE--I-DK-Q-K-N-A--HKQKR--G--C----IIG---C----R--G---C---L------------V-V------I----A--I------N----H-I---Q---KH-Q--I-DHQ-KA-IDDA-----

>Mex23

--D-DD-D-D-LCR-GCRCLR--G----------L-G-L------R--I---I-------D----------------I------AIAA-------K-N--K--H-----Q--K-N-----H-K----A-HQKHHIIE-DI-I---IGG---CIIIGR---C-----C--I-----ID-----D-I--I---E--I-E---------I-D---H-H-KQIDIHQKI-AAA-I--I--D-----L-R----C--A--LEA---D--EI-NDHKH-DA-----I--RGRLD-L---R-GR-A-AR-G-I-DL-L-G----------G--D----I-I----G--L----C-DEA--A-GG--R---CGGCEDI--G-RC--R-L-L-CLC--RIDIAICLCLGG----IA------G--C----I------I-IIH--K--D----I-------Q--K-H---HNHII-D-A--D-K--HNHINND--K-H--KQ--I--I--I--A-H--Q---K-HK-K-K-E-A-ADH---N-Q-I--A------QK------N--N-I-IQKE--I-DK-Q-K-N-A--HKQKR--G--C----DIG---C----R--G---C---C--------AEDID-I------I----E--I-I----H----H-D---Q---KH-Q--I-DHQ-KA-IDDA-----

>UBC100

--D-DD-A-D-LCR-GCGCLR--G----------L-C-L------R--I---I-------D----------------I------E----------K-N--H--N-----Q--K-H-----H-D----K-HQKHHIIE-AI-I---IGG---CIIIGR---C-I---G--C------------I-I--D------I-E---------H-Q---I-H-KQIDDHQKI-AAE-I--L--C-----L-R----D--A----ACA-D--EI-ADE-H-DA-----K--NHNID-I---R-GR-R-RR-G-I-DA-C-G----------I--D----D-L----G--L------D-EGRA-GR--R---AGGCA-I-LG-RC--R-I-I-CLC--RIDIAICLCLEG----LR------G--D----I------I---E--I--Q----K-------N--K-A-AHHNHII-E-A-AD-K--HNHI--I--K-H--KK--N--I--I--A-H--Q---Q-EK-K-Q-D-K-QAA---N-Q-ND-I------QH------A--E-I-QKKK--D-K--Q-K-D-I--AIDLR--G--C----IIG---C----R--G--------------------D-I------D-------------------E-I---Q---KH-Q--I-IHQ-KN-IAIA-----

>HAWK31

--D-DD-A-D-LCR-GCGCLG--G----------------------------I-------I----------------I------A---------------Q-----------Q-N-----H-I----K-HQKHHIIE-DI-I---IGG---CIIIGR---L----RG---------CL----D-I---------I-H---------H-Q---K-H-KQIIDHQKI-AAA-I--L--C-----L-R----D--AI-C-RC--I--EI-ADHKH-DA-----K--NHNID-L---R-G--I-AG-G---GL-C-I-I--------GG-L----D-I----G--L-D--L--GA--A-IG--R---CGGCE-I-LG-RC--R-D-I-CLC--RIDIAICLCLGG----IR------G--D----I------I---E--I--Q----K-------Q--K-H---HNHII-I-A-AA-I-KHNHI--I--K-H--KK--H--I--I--A-H--Q---K-HK-K-I-E-D-Q-N-I-N-Q-I--H------QQ------H--N-KKQAII--E-I--Q-K-NIE--AIDLR--GG-L----R-L---L-------G-------G--------R-----I-----------I--A-------------K---Q---QH-N--K-NKQ-KN-DADA-----

>NWFSC186

--D-DD-A-D-LCR-GCGCLG--G----------------------------I-------I----------------I------A---------------Q-----------Q-N-----H-I----K-HQKHHIIE-DI-I---IGG---CIIIGR---L----RG---------CL----D-I---------I-H---------H-Q---K-H-KQIIDHQKI-AAA-I--L--C-----L-R----D--AI-C-RC--I--EI-ADHKH-DA-----K--NHNID-L---R-G--I-AG-G---GL-C-I-I--------GG-L----D-I----G--L-D--L--GA--A-IG--R---CGGCE-I-LG-RC--R-D-I-CLC--RIDIAICLCLGG----IR------G--D----I------I---E--I--Q----K-------Q--K-H---HNHII-I-A-AA-I-KHNHI--I--K-H--KK--H--I--I--A-H--Q---K-HK-K-I-E-D-Q-N-I-N-Q-I--H------QQ------H--N-KKQAII--E-I--Q-K-NIE--AIDLR--GG-L----R-L---L-------G-------G--------R-----I-----------I--A-------------K---Q---QH-N--K-NKQ-KN-DADA-----

>NWFSC252

--D-DD-A-D-LCR-GCGCLG--G----------------------------I-------I----------------I------A---------------Q-----------Q-N-----H-I----K-HQKHHIIE-DI-I---IGG---CIIIGR---L----RG---------CL----D-I---------I-H---------H-Q---K-H-KQIIDHQKI-AAA-I--L--C-----L-R----D--AI-C-RC--I--EI-ADHKH-DA-----K--NHNID-L---R-G--I-AG-G---GL-C-I-I--------GG-L----D-I----G--L-D--L--GA--A-IG--R---CGGCE-I-LG-RC--R-D-I-CLC--RIDIAICLCLGG----IR------G--D----I------I---E--I--Q----K-------Q--K-H---HNHII-I-A-AA-I-KHNHI--I--K-H--KK--H--I--I--A-H--Q---K-HK-K-I-E-D-Q-N-I-N-Q-I--H------QQ------H--N-KKQAII--E-I--Q-K-NIE--AIDLR--GG-L----R-L---L-------G-------G--------R-----I-----------I--A-------------K---Q---QH-N--K-NKQ-KN-DADA-----

>No7

--D-DD-A-D-LCR-GCGCLR--R----------L-C-----------I---E-------D----------------I------I----------I----H--N-----K--K-H-----H-I----K-HQKHHIIE-AI-D---IGG---CIIIGR---C-I---G--C------------I-I--D------I-E---------H-Q---I-H-KQIDIHQKI-AAA-I--L--C-----L-R----D--A----ACR-C--EI-ADHKH-DA-----K--NHNID-L---R-GR-R-RG-G-I-CL-L-G-------------D----L-L----G--L------DIE-AA-GG--R---CGGCR-L-LG-RC--R-I-I-CLC--RIDIAICLCLGG----IR------G--D----I------I---E--I--Q----K-------Q--K-H---HNHII-A-A-AD-K--HHHI--I--K-H--KK--H--H--K--A-H--Q---QIHK-K-KED-E-AII---N-Q-N--N------QN------N--H-I-QIQK--D-KK-Q-KNI-I--AIDLG--G--CV---II----C----R--G-------I------------D-I------D-------------------A-----Q---KH-I--I-IHQ-QN-IDIA-----

>PnPd26

--D-DD-A-D-LCR-GCGCLG--R----------------------------I-------I----------------I------E----------I----K-----------Q-N-----H-D----K-HQKHHIIE-DI-D---IGG---CIDIGR---C----GG---------CL----I-D---------I-H---------H-Q---K-H-KQIDIHQKI-AAA-I--L--C-----L-R----D--AI-E-RC--I--EI-ADHKE-DA-----K--NHNID-L---R-G--A-AG-R---EL-C-I-I--------GG-L----D-I----G--L-D--L--GA--A-IG--R---CGGCE-I-LG-RC--R-I-I-CLC--RIDIAICLCLGG----IR------G--D----I------I---E--I--Q----K-------Q--K-H---HNHII-E-I-IA-D-KHNHI--I--K-H--KK--H--D--I--A-H--Q---K-HK-Q-I-E-D-Q-N-I-N-Q-I--N------QK------H--NAK-QIDD--I-I--Q-K-HEI--AIDLR--GE-L----R-G---L-------G-------G--------D-----I------D----I--E-------------I---Q---QN-Q--K-NEQ-KN-IIDA-----

>PnPd31

--D-DD-A-D-LCR-GCGCLG--R----------------------------I-------I----------------I------E----------I----K-----------Q-N-----H-D----K-HQKHHIIE-DI-D---IGG---CIDIGR---C----GG---------CL----I-D---------I-H---------H-Q---K-H-KQIDIHQKI-AAA-I--L--C-----L-R----D--AI-E-RC--I--EI-ADHKE-DA-----K--NHNID-L---R-G--A-AG-R---EL-C-I-I--------GG-L----D-I----G--L-D--L--GA--A-IG--R---CGGCE-I-LG-RC--R-I-I-CLC--RIDIAICLCLGG----IR------G--D----I------I---E--I--Q----K-------Q--K-H---HNHII-E-I-IA-D-KHNHI--I--K-H--KK--H--D--I--A-H--Q---K-HK-Q-I-E-D-Q-N-I-N-Q-I--N------QK------H--NAK-QIDD--I-I--Q-K-HEI--AIDLR--GE-L----R-G---L-------G-------G--------D-----I------D----I--E-------------I---Q---QN-Q--K-NEQ-KN-IIDA-----

>OFP41014

--D-DD-A-D-LCR-GCGCLG--G----------------------------I-------I----------------I------A---------------Q-----------Q-N-----H-I----K-HQKHHIIE-DI-I---IGG---CIIIGR---L----RG---------CL----D-I---------I-H---------H-Q---K-H-KQIIDHQKI-AAA-I--L--C-----L-R----D--AI-D-RC--I--EI-ADHKE-DA-----K--NHNID-L---R-G--I-AG-G---EL-C-I-I--------GG-L----D-I----G--L-D--L--GA--A-IG--R---CGGCE-I-LG-RC--R-D-I-CLC--RIDIAICLCLGG----IR------G--D----I------I---E--I--Q----K-------Q--K-H---HNHII-I-A-AA-I-KHNHI--I--K-H--KK--H--I--I--A-H--Q---K-HK-K-I-E-D-Q-N-I-N-Q-I--H------QQ------H--N-IKQAI---E-I--Q-K-NIE--AIDLR--GE-L----R-L---L-------G-------G--------A-----I-----------I--A-------------I---Q---QH-N--K-NIQ-KN-DADA-----

>PnMi06

--D-DD-A-D-LDR-GCGDLGIIG--------------D------L--AG--I-------E--------A-------K------E----N-----A----K--I-----I--Q-N-----I-I----IQHQKAHIAE-DEEIRGCIGGLIECIIIG----------G---------CL----D-I-EA------INH-DKKDIH--H-Q---K-E-IDIDIHQKI-AAA-I--L--C-----L-R----D--AI-E-RC--I--EI-ADHKE-DA-----K--NHNID-L---R-GR-AEA--G-IDILEG-I------------------D-I----G--L-D--L--GA--A-IG--R---CGGCE-I-LG-RC--RCI-I--LC--RIDIAICLCIGG----IRER----G--I-AAAI------I---A--I-IQ----KA------K--K-Q-D-HNHII-I-E-IA-IIKHN-I--IH-K-H--KK--H--D--I--A-H--Q---K-HK-K-I-E-D-Q-N-I-N-Q-I--E----D-QE------N--E-I-QADA--D-K-EQ-K-NDE--EIDLR--GG-L----R-G---L-------GRIGL---G--------R-----I------I----I--A------I----K-KH--QK--QN-Q--K-NKQ-KN-DIDA-----

>PnMt45

--D-DD-A-D-LCR-GCGCLG--G----------C-----------------I------------------------I------D----------E----I--H-----Q--K-H-----H-Q----I-HQKHHI-A-II-I---IGG---CCGIGR---C-L--G---R------R-----I-I--I---E--K-K-----------K---N-H-KQIQHHQKI-AAE-I--I--I-----L-R----C-GL----IA--A--EI-NQHKH-DA-----I--RGRLC-L-RER-R----A--G-C--L-C-I----------ED-CC--GI-ILR--G--L----L---AA-R-RRD-R---CGGCE-I-IG-RC--R-D-I-CLL--GLDIAICLCLGG----L--A----G----G--I------I-I-A--I--K-QA--N------Q--K-H---HNHII-I-E--D-I--NQHN--HI-K-H--K---K--IA-I--A-H--Q---K-HK-K-K-K-E-A-N---N-Q----KN---D-KH------H-AA-I-HNHK--E-DK-KEK-N-H--NKQKR--R--C---LR-----G----C-LG----------------------E------A----A--A-------------Q-------NH----KKNHK-KA-IADA-----

>PnSb44

--D-DD-A-D-LCR-GCGCLG--G----------C-----------------I------------------------I------D----------E----I--H-----Q--K-H-----H-Q----I-HQKHHI-A-II-I---IGG---CCGIGR---C-L--G---R------R-----I-I--I---E--K-K-----------K---N-H-KQIQHHQKI-AAE-I--I--I-----L-R----C-GL----IA--A--EI-NQHKH-DA-----I--RGRLC-L-RER-R----A--G-C--L-C-I----------ED-CC--GI-ILR--G--L----L---AA-R-RRD-R---CGGCE-I-IG-RC--R-D-I-CLL--GLDIAICLCLGG----L--A----G----G--I------I-I-A--I--K-QA--N------Q--K-H---HNHII-I-E--D-I--NQHN--HI-K-H--K---K--IA-I--A-H--Q---K-HK-K-K-K-E-A-N---N-Q----KN---D-KH------H-AA-I-HNHK--E-DK-KEK-N-H--NKQKR--R--C---LR-----G----C-LG----------------------E------A----A--A-------------Q-------NH----KKNHK-KA-IADA-----

>PnPd57

--D-DD-D-D-LCR-GCRCLR--G---------------------R--E---I-------A----------------I-----------------K-------------Q--K-N-----H-K----A-HQKHHIIE-DI-I---IGG---CIIIGR---C-----CG-L------D-----D-I--I---E--I-E---------H-K---H-H-KQIDIHQKI-AAA-I--I--D-----L-R----C--A--LEA---D--EI-NDHKH-DA-----I--RGRLD-L---R-GR-A-AR-G-L-DL-L-R----------G--D----I-I----G--L----C-IEA--A-AG--R---CGGCE-I-LG-RC--R-I-I-CLC--RIDIAICLCLGG----IR------G--D----I------I---E--I--Q----K-------Q--K-H---HNHII-D-A--D-K--HNHI--I--K-H--KK--N--D--I--A-H--Q---K-HK-K-D-E-A-ADH---N-K-I--A------QK------N--N-A-NQKE--I-DK-Q-K-N-A--HKQKR--G--C----IIG---C----R--G--------------------D-I------I----E--I-----------------Q---KH-Q--I-DHQ-KA-IDDA-----

>PnPd68

--D-DD-D-D-LCR-GCRCLR--G---------------------R--E---I-------A----------------I-----------------K-------------Q--K-N-----H-K----A-HQKHHIIE-DI-I---IGG---CIIIGR---C-----CG-L------D-----D-I--A---E--I-E---------H-K---H-H-KQIDIHQKI-AAA-I--I--D-----L-R----C--A--LEA---D--EI-NDHKH-DA-----I--RGRLD-L---R-GR-A-AR-G-L-DL-L-R----------G--D----I-I----G--L----C-IEA--A-AG--R---CGGCE-I-LG-RC--R-I-I-CLC--RIDIAICLCLGG----IR------G--D----I------I---E--I--Q----K-------Q--K-H---HNHII-D-A--D-K--HNHI--I--K-H--KK--N--D--I--A-H--Q---K-HK-K-D-E-A-ADH---N-K-I--A------QK------N--N-A-NQKE--I-DK-Q-K-N-A--HKQKR--G--C----IIG---C----R--G--------------------D-I------I----E--I-----------------Q---KH-Q--I-DHQ-KA-IDDA-----

>Ps102

--D-DD-D-D-LCR-GCRCLR--G---------------------R--E---I-------A----------------I-----------------K-------------Q--K-N-----H-K----A-HQKHHIIE-DI-I---IGG---CIIIGR---C-----CG-L------D-----D-I--I---E--I-E---------H-K---H-H-KQIDIHQKI-AAA-I--I--D-----L-R----C--A--LEA---D--EI-NDHKH-DA-----I--RGRLD-L---R-GR-A-AR-G-L-DL-L-R----------G--D----I-I----G--L----C-IEA--A-AG--R---CGGCE-I-LG-RC--R-I-I-CLC--RIDIAICLCLGG----IR------G--D----I------I---E--I--Q----K-------Q--K-H---HNHII-D-A--D-K--HNHI--I--K-H--KK--N--D--I--A-H--Q---K-HK-K-D-E-A-ADH---N-K-I--A------QK------N--N-A-NQKE--I-AK-Q-K-N-A--HKQKR--G--C----IIG---C----R--G--------------------D-I------I----E--I-----------------Q---KH-Q--I-IHQ-KA-IDDA-----

>Ps147

--D-DD-D-D-LCR-GCRCLR--G---------------------R--E---I-------A----------------I-----------------K-------------Q--K-N-----H-K----A-HQKHHIIE-DI-I---IGG---CIIIGR---C-----CG-L------D-----D-I--I---E--I-E---------H-K---H-H-KQIDIHQKI-AAA-I--I--D-----L-R----C--A--LEA---D--EI-NDHKH-DA-----I--RGRLD-L---R-GR-A-AR-G-L-DL-L-R----------G--D----I-I----G--L----C-IEA--A-AG--R---CGGCE-I-LG-RC--R-I-I-CLC--RIDIAICLCLGG----IR------G--D----I------I---E--I--Q----K-------Q--K-H---HNHII-D-A--D-K--HNHI--I--K-H--KK--N--D--I--A-H--Q---K-HK-K-D-E-A-ADH---N-K-I--A------QK------N--N-A-NQKE--I-DK-Q-K-N-A--HKQKR--G--C----IIG---C----R--G--------------------D-I------I----E--I-----------------Q---KH-Q--I-DHQ-KA-IDDA-----

>Hobart5

-ID-DA-A-D-LCR-GCRCLG--G----------L-G--------RL-IADIILRL----I---ILRIIIIAIIIKNI------INK--NIIIINK----QHIADI---Q--K-N-----H-K----A-HQKHHIIE-DI-I---IGG---CIVIGR---C----GG--L------C-----I-E-AA---D--I-H---------N-Q---K-H-KQIIIHQKI-AAA-I--L--C-----L-R----D--AIIC-RL-----EI-ADHKH-DA-----K--NHNID-L---R-G--A-ARCG----L-C-I-I--------GG-C----D-D----G--L----L--GR--R-IA--R---CGGLE-D-LG-GC--R---ILCLC--GLDLAICLCIGG----IR------G--C-L--I------I-A-E--H-HQ----K-------I--KQI-I-HNHII-I-ENIE-H-KHNHN--I--K-H--KK--N-----A--A-HQ-Q---I-HK-D-I-K-K-K-H---H-QAA--H------QQ---------I-I-HNKI--H-KEEQ-K-N-A--AIDIR--GC-L----RIE--AL-------G---L---L-R------RI--E-I------IAAAKK--N-----------N-I---Q---HE----K-NHQ-KA-IDAAI----

>PnMi16

--D-DD-A-D-LDR-GCGDLGIIG--------------D------L--AG--I-------E--------A-------K------E----N-----A----K--I-----I--Q-N-----I-I----IQHQKAHIAE-DEEIRGCIGGLIECIIIG----------G---------CL----D-I-EA------INH-DKKDIH--H-Q---K-E-IDIDIHQKI-AAA-I--L--C-----L-R----D--AI-E-RC--I--EI-ADHKE-DA-----K--NHNID-L---R-GR-AEA--G-IDILEG-I------------------D-I----G--L-D--L--GA--A-IG--R---CGGCE-I-LG-RC--RCI-I--LC--RIDIAICLCIGG----IRER----G--I-AAAI------I---A--I-IQ----KA------K--K-Q-D-HNHII-I-E-IA-IIKHN-I--IH-K-H--KK--H--D--I--A-H--Q---K-HK-K-I-E-D-Q-N-I-N-Q-I--E----D-QE------N--E-I-QADA--D-K-EQ-K-NDE--EIDLR--GG-L----R-G---L-------GRIGL---G--------R-----I------I----I--A------I----K-KH--QK--QN-Q--K-NKQ-KN-DIDA-----

>MLR2013

--D-DD-A-D-LCR-GCRCLG--G----------L-G--------R------I-------IADIIIAIIIIAIIIIAI------DEI--AD----K----Q--N-----Q--K-N-----H-K----A-HQKHHIIE-DI-I---IGG---CIIIGR---C----GG--L------C-----I-E-AA---D--I-H---------N-Q---K-H-KQIIIHQKI-AAA-I--L--C-----L-R----D--AIIC-RL-----EI-ADHKH-DA-----K--NHNID-L---R-G--A-ARCR----L-C-I-I--------GG-C----D-D----G--L----L--GR--R-IA--R---CGGLE-D-LG-GC--R---ILCLC--GLDLAICLCIGG----IR------G--C-L--I------I-A-E--H-HQ----K-------I--KQI-I-HNHII-I-ENIE-H-KHNHN--I--K-H--KK--N-----A--A-HQ-Q---I-HK-D-I-K-K-K-H---H-QAA--H------QQ---------I-I-HNKI--H-KEEQ-K-N-A--AIDIR--GC-L----RIG--CL-------G---L---L-AAIEIIAR---A-I-----------K--N-----------N-D---Q---HH-Q--K-NHQ-KA-IDDA-----

>NWFSC188

--D-DD-A-D-LCR-GCACLG--R----------L-C-L------R------D-------I----------------I------E----------K-N--H--N-----K--K-H-----H-I----A-HQKHHIIA-AI-A---IGG---CIIIGR---C-I--GC--I------------I-D---------I-E---------H-Q---A-H-KQIIIHQKI-AAE-I--L--C-----L-R----D--A----ACA-D--EI-ADE-H-DA-----K--NHNID-L---R-G--RLLR-GLI--L-L-G----------G--D------I----G--LRA--L--LLL-I-I---R---CGGLG-C-LL-AC--R-R-L-CLC--DIDIAICLCLGG----IR------G--D----I------I---E--Q--K----I-------Q--K-N---HNHII-I-A-II-I--HNHH--K--K-H--AN--N-HK--I--A-N--Q---K-HK-I-I-H-N-N-NDK-N-Q-I--A------QK------N--N---INKK--H-HK-K-K-N-E--AIDLR--G--C----III---C----R--G----------------------I------I----I--A-------------I---Q---KH-I--I-IHQ-KN-IDDA-----

>T5

--D-DD-A-D-LCR-GCGCLG--G----------L-----------------I------------------------I------A---------------E--H-----Q--Q-H-----H-K----D-HQKHHIIE-II-I---IGG---CDIIGR---C-I--GC--L------R-----D-I--I---E--K-N---------H-Q---I-H-KQIDIHQKI-AAE-I--I--D-----L-R----C-AE--D-AI--I--EI-ADHKH-DA-----I--RGGLI-L---R-G----AR-R-C-CL-C-R----------RDLC---GLDI-I--G--L-D--A------R--G--R---CGGCE-I-LG-RC--R-I-L-CLL--GIDIAICLCLGG----LRED--LLG--L----I------I-I-AI-I-NQNNAAKN-----KQ--I-H---HNHII-I-E--D-K--NNHN--I--K-H--KQ--N--IE-IIDA-H--Q---Q-HK-K-K---E-A-A---N-Q-I--A----I-NQ------H-NK-K-HHHI--HKKA-Q-K-N-D--NKQKR--G--C-D-I--G--CL----R-LR-------C--------------I------A----D--I------AA---H-K---NIK-NH-Q--I-AHQ-KA-IDDA-----

>PnMi01

--D-DD-A-D-LCR-GCGCCG--G----------L----------R------I-------IG-LLLRLLD-------I------A-N--N-----K-HNHQDKH-----Q--Q-H-----H-I----K-HQKHHIIA-DI-E---IGG---CIIIGR---E----GG--L------C-----I-E-AA---D--I-H---------N-Q---Q-E-KQIIIHQKI-AAA-I--L--C-----L-R----D--AA-C-RC--I--EI-ADHKH-DA-----K--NHNID-L---R-G--A-RRCG----L-D-I-I--------GG-C----D-D----G--L----I--EA--A-IR--R---CGGCG-C-LG-GC--R---IICLC--GICLRLCLCLEG----LR------G--D----I------I-A-AQ-K-HQ----A-------A--A-H---HNHII-IIANKN-H-KHNHI--I--K-H--KK--A-----A--N-HKHQ---K-HK-K-I-E-D-E-A-I-N-QAA--H------QQ---------I-I-INK---H-KKEQ-K-NDE--EIDLR--GC-L----RIG--CG-------G---R---L------------E-E-----------A--A-----------N-K---Q---KH-Q--K-NHQ-KN-IDDA-----

>Nissum3

--D-DD-A-D-LCR-GCGCLG--G----------L-----------------I------------------------I------A---------------E--H-----Q--Q-H-----H-K----D-HQKHHIIE-II-I---IGG---CDIIGR---C-R--GC--L------R-----D-I--A---E--K-N---------H-Q---K-H-KQIDIHQKV-AAE-I--I--D-----L-R----C-GE--I-AI--D--EI-AQHKH-DA-----I--RGRLL-L---R-GL---I--R-C-CL-C-R----------RD-C----IEI-D--L--L-E--L---C--R-AG--R---CGGCE-I-LG-RC--R-D-L-CLL--GIDIAICLCLGG----IAED--LLG--L-R--I------I-I-AI-K-NQNNA-IA-----IQ--K-H---HNHII-I-E--I-K--NNHN--I--K-H--KQ--N--IE-IIDA-H--Q---K-HK-K-K---H-N-N---N-D-I--I----I-ID------H-AK-K-HNHI--HKDN-Q-K-N-H--NKQKR--G--C---LR-G--CL----R-LR-------C------------I-I------I----D--D------I----H-K---NIK-NH-Q--K-NHQ-KA-IDDA-----

>PnMi28

--D-DD-A-D-LCR-GCGCCG--G----------L----------R------I-------IG-LLLRLLD-------I------A-N--N-----K-HNHQDKH-----Q--Q-H-----H-I----K-HQKHHIIA-DI-E---IGG---CIIIGR---E----GG--L------C-----I-E-AA---D--I-H---------N-Q---Q-E-KQIIIHQKI-AAA-I--L--C-----L-R----D--AA-C-RC--I--EI-ADHKH-DA-----K--NHNID-L---R-G--A-RRCG----L-D-I-I--------GG-C----D-D----G--L----I--EA--A-IR--R---CGGCG-C-LG-GC--R---IICLC--GICLRLCLCLEG----LR------G--D----I------I-A-AQ-K-HQ----A-------A--A-H---HNHII-IIANKN-H-KHNHI--I--K-H--KK--A-----A--N-HKHQ---K-HK-K-I-E-D-E-A-I-N-QAA--H------QQ---------I-I-INK---H-KKEQ-K-NDE--EIDLR--GC-L----RIG--CG-------G---R---L------------E-E-----------A--A-----------N-K---Q---KH-Q--K-NHQ-KN-IDDA-----

>PnTb10

--D-DD-A-D-LCR-GCGCCG--G----------L----------R------I-------IG-LLLRLLD-------I------A-N--N-----K-HNHQDKH-----Q--Q-H-----H-I----K-HQKHHIIA-DI-E---IGG---CIIIGR---E----GG--L------C-----I-E-AA---D--I-H---------N-Q---Q-E-KQIIIHQKI-AAA-I--L--C-----L-R----D--AA-C-RC--I--EI-ADHKH-DA-----K--NHNID-L---R-G--A-RRCG----L-D-I-I--------GG-C----D-D----G--L----I--EA--A-IR--R---CGGCG-C-LG-GC--R---IICLC--GICLRLCLCLEG----LR------G--D----I------I-A-AQ-K-HQ----A-------A--A-H---HNHII-IIANKN-H-KHNHI--I--K-H--KK--A-----A--N-HKHQ---K-HK-K-I-E-D-E-A-I-N-QAA--H------QQ---------I-I-INK---H-KKEQ-K-NDE--EIDLR--GC-L----RIG--CG-------G---R---L------------E-E-----------A--A-----------N-K---Q---KH-Q--K-NHQ-KN-IDDA-----

>AL101

--D-DD-A-D-LCR-GCGCLG--G----------L-G-R------LE-A---I--D----I--------A-------A------AAAAAN-----K----Q--N-----Q--Q-H-----H-I----K-HQKHHIIE-DI-I---IGG---CIIIGRCGGL----GG--LIAGLLGCL----D-I-AEIDID--INH-KNHQDHQKH-Q---K-H-KQIIIHQKI-AAA-I--L--C-----L-R----D--AI-C-RC--I--EI-ADHKH-DA-----K--NHNID-L---R-GR-A-AL-G-IDAL-GGI-I--------EI-I----D-L----G--L-D--L--GA--A-IG--R---CGGCE-I-LG-AC--R-D-I-CLC--RIDIAICLCLGG----IR------G--D----I------I---E--I--Q----K-------Q--K-H---HNHII-E-A-AA-D-KHNHI--I--K-H--DK--H--I--I--A-H--Q---K-HK-K-I-E-D-Q-N-A-N-QHI--I----D-QQ------H--E-I-QNEI--E-K--Q-K-NDE--AIDLG--GG-L----R-G---L-------G-------G--------------I------E----D--D-------------A---Q---QN-Q--K-NKQ-KN-DADA-----

>CBA56

--D-DD-A-D-LCR-GCGCLG--G----------L-G-R------LE-A---I--D----I--------A-------A------AAAAAN-----K----Q--N-----Q--Q-H-----H-I----K-HQKHHIIE-DI-I---IGG---CIIIGRCGGL----GG--LIAGLLGCL----D-I-AEIDID--INH-KNHQDHQKH-Q---K-H-KQIIIHQKI-AAA-I--L--C-----L-R----D--AI-C-RC--I--EI-ADHKH-DA-----K--NHNID-L---R-GR-A-AL-G-IDAL-GGI-I--------EI-I----D-L----G--L-D--L--GA--A-IG--R---CGGCE-I-LG-AC--R-D-I-CLC--RIDIAICLCLGG----IR------G--D----I------I---E--I--Q----K-------Q--K-H---HNHII-E-A-AA-D-KHNHI--I--K-H--DK--H--I--I--A-H--Q---K-HK-K-I-E-D-Q-N-A-N-QHI--I----D-QQ------H--E-I-QNEI--E-K--Q-K-NDE--AIDLG--GG-L----R-G---L-------G-------G--------------I------E----D--D-------------A---Q---QN-Q--K-NKQ-KN-DADA-----

>CBA60

--D-DD-A-D-LCR-GCGCLG--G----------L-G-R------LE-A---I--D----I--------A-------A------AAAAAN-----K----Q--N-----Q--Q-H-----H-I----K-HQKHHIIE-DI-I---IGG---CIIIGRCGGL----GG--LIAGLLGCL----D-I-AEIDID--INH-KNHQDHQKH-Q---K-H-KQIIIHQKI-AAA-I--L--C-----L-R----D--AI-C-RC--I--EI-ADHKH-DA-----K--NHNID-L---R-GR-A-AL-G-IDAL-GGI-I--------EI-I----D-L----G--L-D--L--GA--A-IG--R---CGGCE-I-LG-AC--R-D-I-CLC--RIDIAICLCLGG----IR------G--D----I------I---E--I--Q----K-------Q--K-H---HNHII-E-A-AA-D-KHNHI--I--K-H--DK--H--I--I--A-H--Q---K-HK-K-I-E-D-Q-N-A-N-QHI--I----D-QQ------H--E-I-QNEI--E-K--Q-K-NDE--AIDLG--GG-L----R-G---L-------G-------G--------------I------E----D--D-------------A---Q---QN-Q--K-NKQ-KN-DADA-----
